# Supplementary material for: Evaluation of a personalised adherence intervention to improve photoprotection in adults with Xeroderma Pigmentosum (XP): protocol for the trial of XPAND
Source: BMJ Open. 2019 Jul 17;9(7):e028577. doi: 10.1136/bmjopen-2018-028577 (PMC6661555; doi:10.1136/bmjopen-2018-028577)
Supplement: Supplementary data [file bmjopen-2018-028577supp001.pdf]

XPAND

Your daily UVR diary

Study ID

« 21 DAYS
« 14 DAYS
« 7 DAYS

Your daily UVR diary

People with XP tell us that it can be hard to protect against UVR all the time and we are interested in what works for you.

This booklet has your daily diary sheets to record when you were outside and whether you did anything to protect against UVR each day.

- ★ You only need to record the time you spend outside if it was for **10 minutes or longer**
- ★ "OUTSIDE" is any amount of time longer than 10 minutes you **did not spend in a building**
  "OUTSIDE" includes time spent travelling in cars, buses or trains
- ★ Our grid is split into 15 minute blocks, start your line at the nearest block

We are interested in your views about your UVR protection each day and your general mood. There are four short questions to complete.
Don't take too long thinking about your responses. Your immediate reaction to each question will probably be more accurate than a long thought out response. Please circle the number that best corresponds to your views on that day.

- ★ **If you can't remember exactly or you are unsure give us your best guess!**
- ★ Try to fill out the diary at the end of each day. It will be easier to do when it is fresh in your mind. If you do forget, don't worry! Fill it out as soon as you remember.

? **If you are unsure or have any questions contact Lesley on 07775111823 or email her at Lesley.Foster@gstt.nhs.uk**

Thank you very much for taking the time to complete the daily diary.

King's College London

Guy's and St Thomas' NHS
NHS Foundation Trust

Daily diary example overleaf

Your daily UVR diary

Example:

1. **Date (day of week)** 14 May (Saturday)
2. **Did you go outside today?** (Please tick) **YES** ✓ **NO** ☐ If no, skip to question 4
3. **Let us know when you were outside by marking a line on the diary**

	6am	7am	8am	9am	10am	11am	12pm	1pm	2pm	3pm	4pm	5pm	6pm	7pm	8pm	9pm	
I was outside																	

4. **Let us know if you wore any of these things today by marking a line on the diary**

	6am	7am	8am	9am	10am	11am	12pm	1pm	2pm	3pm	4pm	5pm	6pm	7pm	8pm	9pm	
Hat																	
Glasses																	
Scarf or facebuff																	
Hoodie worn up																	
Face visor																	

5. **If you put sunscreen on your face or used lip sun block tell us each time you did this by ticking the diary**

	6am	7am	8am	9am	10am	11am	12pm	1pm	2pm	3pm	4pm	5pm	6pm	7pm	8pm	9pm	
Sun screen	✓				✓				✓								
Lip sunblock	✓																

How would you describe your mood today?

0 1 2 3 4 5 6 7 8 9 (10)

Very negative — Very positive

How important was UVR protection of your face today compared to other things you wanted/needed to do?

0 1 2 3 (4) 5 6 7 8 9 10

Not at all — Very important

How much do you agree that UVR protection of your face today was something you did automatically without thinking?

0 1 2 3 4 5 6 (7) 8 9 10

Strongly disagree — Strongly agree

How confident are you that you can protect your face well tomorrow, even if other things get in the way?

0 1 2 3 4 (5) 6 7 8 9 10

Not at all — Very much so
