# Supplementary material for: Evaluation of a personalised adherence intervention to improve photoprotection in adults with Xeroderma Pigmentosum (XP): protocol for the trial of XPAND
Source: BMJ Open. 2019 Jul 17;9(7):e028577. doi: 10.1136/bmjopen-2018-028577 (PMC6661555; doi:10.1136/bmjopen-2018-028577)
Supplement: Supplementary data [file bmjopen-2018-028577supp002.pdf]

Participant study code________________

XPAND
Enhancing Photoprotection Activities - New Directions

Xeroderma Pigmentosum National Service
2nd Floor, South Wing
St Thomas' Hospital
Westminster Bridge Road
London SE1 7EH

KING'S College LONDON

Guy's and St Thomas' NHS
NHS Foundation Trust

We are interested in your own personal views about XP and ultraviolet (UVR) protection. There are no right or wrong answers.

Please circle the number that best corresponds to your views:

1. How confident are you that you can lower your overall exposure to daylight by shifting when or how long you do outdoor daily activities?

0 1 2 3 4 5 6 7 8 9 10
not at all *very confident*

2. How confident are you that you can wear all the protective clothing you want, to protect yourself when outdoors?

0 1 2 3 4 5 6 7 8 9 10
not at all *very confident*

3. How confident are you that you can correctly apply sunscreen so that you have best protection from UVR?

0 1 2 3 4 5 6 7 8 9 10
not at all *very confident*

4. Thinking about using clothing (e.g., hat, glasses, scarf or buff, hoodie) to achieve a high level of photoprotection of your face from UVR over the next 4 weeks, how confident are you that you can photoprotect even if/when………

A. unexpected things get in the way

0 1 2 3 4 5 6 7 8 9 10

not at all *very confident*

B. you are somewhere new

0 1 2 3 4 5 6 7 8 9 10

not at all *very confident*

C. you are in a rush

0 1 2 3 4 5 6 7 8 9 10

not at all *very confident*

D. you are with new people

0 1 2 3 4 5 6 7 8 9 10

not at all *very confident*

E. you don't want to look different

0 1 2 3 4 5 6 7 8 9 10

not at all *very confident*

F. you are feeling low

0 1 2 3 4 5 6 7 8 9 10

not at all *very confident*

G. you are feeling happy

0 1 2 3 4 5 6 7 8 9 10

not at all *very confident*

H. you are pressured not to protect by family or friends

0 1 2 3 4 5 6 7 8 9 10

not at all *very confident*

I. It might be physically uncomfortable (e.g., too hot)

0 1 2 3 4 5 6 7 8 9 10

not at all *very confident*

5. How confident you are that you will correctly apply sunscreen to your face over the next 4 weeks even if/when:

A. unexpected things get in the way

0 1 2 3 4 5 6 7 8 9 10

not at all *very confident*

B. you are somewhere new

0 1 2 3 4 5 6 7 8 9 10

not at all *very confident*

C. you are in a rush

0 1 2 3 4 5 6 7 8 9 10

not at all *very confident*

D. you are with new people

0 1 2 3 4 5 6 7 8 9 10

not at all *very confident*

E. you don't want to look different

0 1 2 3 4 5 6 7 8 9 10

not at all *very confident*

F. you are feeling low

0 1 2 3 4 5 6 7 8 9 10

not at all *very confident*

G. you are feeling happy

0 1 2 3 4 5 6 7 8 9 10

not at all *very confident*

H. you are pressured not to protect by family or friends

0 1 2 3 4 5 6 7 8 9 10

not at all *very confident*

I. It might be physically uncomfortable (e.g., sticky)

0 1 2 3 4 5 6 7 8 9 10

not at all *very confident*
