# Supplementary material for: Evaluation of a personalised adherence intervention to improve photoprotection in adults with Xeroderma Pigmentosum (XP): protocol for the trial of XPAND
Source: BMJ Open. 2019 Jul 17;9(7):e028577. doi: 10.1136/bmjopen-2018-028577 (PMC6661555; doi:10.1136/bmjopen-2018-028577)
Supplement: Supplementary data [file bmjopen-2018-028577supp003.pdf]

XPAND
Enhancing Photoprotection Activities - New Directions

Xeroderma Pigmentosum National Service
2nd Floor, South Wing
St Thomas' Hospital
Westminster Bridge Road
London SE1 7EH

We know people adjust ultraviolet radiation (UVR) protection to fit in with their everyday life. We are interested in how *__you__* protected against UVR in the last week.

Please circle the number that best corresponds to your views:

Thinking about your UVR protection over the last week

1. When you went outside, how often did you protect your face against UVR using protective clothing?

0	1	2	3	4	5	6	7	8	9	10
never										*all the time*

2. How many days did you apply sunscreen on your face when getting ready in the morning?

0	1	2	3	4	5	6	7
none							*every day of the week*

3. When you went outside for longer periods, how often did you reapply sunscreen on your face?

0	1	2	3	4	5	6	7	8	9	10
not at all										*always*

4. On average, how many hours did you spend outside per day between 7am and 7pm?

a) *Never*

b) *30min or less*

c) *31 min to 1 hour*

d) *2 hours*

e) *3 hours*

f) *4 hours*

g) *5 hours*

h) *6 hours*

i) *7 hours*

j) *8 hour or more*

5. On average, how many hours did you spend outside per day between 11am and 3pm?

   a) *Never*

   b) *30min or less*

   c) *31 min to 1 hour*

   d) *2 hours*

   e) *3 hours*

   f) *4 hours*

THANK YOU VERY MUCH
