# Supplementary material for: Evaluation of a personalised adherence intervention to improve photoprotection in adults with Xeroderma Pigmentosum (XP): protocol for the trial of XPAND
Source: BMJ Open. 2019 Jul 17;9(7):e028577. doi: 10.1136/bmjopen-2018-028577 (PMC6661555; doi:10.1136/bmjopen-2018-028577)
Supplement: Supplementary data [file bmjopen-2018-028577supp004.pdf]

IRAS 236877

XPAND
Enhancing Photoprotection Activities - New Directions
Xeroderma Pigmentosum National Service
2nd Floor, South Wing
St Thomas' Hospital
Westminster Bridge Road
London SE1 7EH

Feedback Questionnaire

We are interested in your opinion of the XPAND programme. Your views will contribute to development of the programme for people newly diagnosed with XP. We are interested in your views, there are no right or wrong answers.

Please tick the box to show how much you agree or disagree with the following statements about the XPAND programme.

1. Overall the programme was........

	1 Strongly disagree	2	3	4	5 Completely agree
Relevant to me					
Interesting					
Helpful					
Easy to understand					
Too long					
Too short					

Comments

Please give us reasons for your answers and say if there is anything you'd like us to change….

2. How helpful did you find the different parts of the programme?

	1<br>Not at all helpful	2	3	4	5<br>Very helpful
Face to face meeting with XPAND team member					
Telephone or skype calls with XPAND team member					
Printed materials					
Text messages					

Comments<br>Please explain why you found parts helpful or not helpful

3. Please tick the box to show how much you agree or disagree with the following statements about the XPAND programme?

The programme helped me to improve my UV protection

Strongly disagree	1	2	3	4	5	Completely agree

Comments<br>Tell us what you are doing differently? If nothing has changed please write "no change".

4. The programme has made living with XP easier in other ways (e.g. feeling generally more confident or closer to family and friends)

Strongly disagree	1	2	3	4	5	Completely agree

Comments<br>Could you tell us how? If nothing has changed please write "no change".

5. Overall the programme has changed……..

	1 Strongly disagree	2	3	4	5 Completely agree
My motivation to protect					
My confidence in managing protection					
My knowledge					
My routines in managing protection					
My ways of coping when life gets in the way of protection					
How my friends and family support me					

Comments<br>Can you tell us how these things have changed? If nothing has changed please write “no change”.

6. Would you recommend taking part in XPAND to other people with XP?

Yes ☐ No ☐

Please add any other comments here

Comments

THANK YOU VERY MUCH
