# Supplementary material for: Evaluation of a personalised adherence intervention to improve photoprotection in adults with Xeroderma Pigmentosum (XP): protocol for the trial of XPAND
Source: BMJ Open. 2019 Jul 17;9(7):e028577. doi: 10.1136/bmjopen-2018-028577 (PMC6661555; doi:10.1136/bmjopen-2018-028577)
Supplement: Supplementary data [file bmjopen-2018-028577supp005.pdf]

XPAND

Enhancing Photoprotection Activities - New Directions

Service use and Financial costs of XP to you and your family in the last 6 months

Xeroderma Pigmentosum National Service
2nd Floor, South Wing
St Thomas' Hospital
Westminster Bridge Road
London SE1 7EH

These questions are about the services that you have accessed and the financial cost that the XP causes for you and your family. It is fine if you make your best guess for questions where you are not sure of the answer.

1. **<u>In the last 6 months,</u> have you attended the XP clinic at Guy's and St Thomas' hospital? Please complete the following sentence:**

   a) **I have attended the clinic ____ times in the last 6 months**

If you did not attend the XP clinic in the last 6 months, go to question 2.

If you have attended the clinic in the last 6 months:

   b) **How long did the journey from home to the clinic take? _____ hours _____ minutes**

   c) **Did you have to spend the night away from home in order to visit the clinic?**
   Yes, I stayed in hospital
   Yes, but not in hospital
   No

   d) **Which specialists do you recall seeing on your visit(s) to the XP clinic? Please tick yes or no for each specialist**

**Specialist**	**Yes, I saw this person**	**No, I did not see this person**
Dermatologist		
Ophthalmologist		
Neurologist		
Geneticist		
Psychologist		

Specialist Nurse		
Plastic Surgeon		

2. Please complete the following sentence:
In the last 6 months, I have been to Accident and Emergency because of my XP ____ times.

3. Please give details of any hospital outpatient appointments you have had in the last 6 months <u>because of your XP. Do not include trips to the XP clinic at Guy's and St Thomas' here.</u>

**Speciality**	**Number of XP related visits in the last 6 months**	**If you paid for this service, roughly how much did it cost per visit?**	**Roughly how long did it take to travel to the appointment?**
Dermatology			___ hours __ minutes
Ophthalmology			___ hours __ minutes
Neurology			___ hours __ minutes
Paediatrics			___ hours __ minutes
Plastic Surgery			___ hours __ minutes
Psychologist			___ hours __ minutes

4. What surgery you have had in the <u>LAST 6 MONTHS</u>? (day or clinic surgery - where you did <u>not</u> stay in hospital overnight)

**Type of surgery**	**Approximate number of surgeries**	**If you paid for this service, approximately how much did it cost per episode of surgery?**	**Roughly how long did it take to travel for the surgery?**
Skin biopsy			___ hours __ minutes
Surgery to the skin			___ hours __ minutes
Surgery to the eyes			___ hours __ minutes
Other XP related surgery<br>What sort of surgery was it?<br>____________________			___ hours __ minutes

5. Please complete the following sentence.
In the last 6 months, I have been admitted to hospital and stayed overnight ___ times because of my XP. In this time, I spent a total of ___ nights in hospital.

6. In the last 6 months, which of these services have you visited or been visited at home by, because of your XP?

	**Number of appointments at surgery or clinic**	**Number of home visits**	**If you paid, approximately how much did it cost per visit?**	**How long did it take to travel for the appointment each time if it was not at home?**
General practitioner				___ hours __ minutes
Nurse				___ hours __ minutes
Physiotherapist				___ hours __ minutes
Psychologist , Counsellor or Psychotherapist				___ hours __ minutes
‘Alternative’ medicine or ‘complementary’ therapist				___ hours __ minutes
Occupational therapist				___ hours __ minutes
	**Number of appointments at surgery or clinic**	**Number of home visits**	**If you paid, approximately how much did it cost per visit?**	**How long did it take to travel for the appointment each time if it was not at home?**

Social worker				___ hours __ minutes
Home help or home care worker				___ hours __ minutes
Self-help or support group				___ hours __ minutes

7. Please list any tests you remember that you have had done in the LAST 6 MONTHS because of your XP

**Service**	**How many times?**	**If you paid, approximately how much did it cost?**
MRI (Magnetic Resonance Image) brain scan		
Audiometry		
Nerve testing (nerve conduction or EMG test)		
Blood test		
Other investigations / tests<br><br>What was it?<br><br>…………………………………………………………		

8. In the last 6 months, have you received help from friends or relatives with any of the following tasks, as a result of your XP?

**Type of help**	**Please Circle**	**Approximate number of hours they help you in a week**
Child Care<br>(circle 'No' if you have no children)	**No Yes**	
Personal care<br>*(for example washing, dressing)*	**No Yes**	
Help in the house<br>(for example cooking or cleaning)	**No Yes**	
Help outside the house<br>(for example shopping or transport)	**No Yes**	
Going with you to medical appointment	**No Yes**	

9. If you answered yes to any help in the previous question, have your helper(s) taken any time out of work or education to help you as a result of your XP?
If there are part-days to consider, assume that 1 day = 8 hours.
______________ days taken off from work in the last 6 months
______________ days taken off from education in the last 6 months

10. What is your employment status today? Please tick the one that best describes your situation

Paid employment - full-time	
Paid employment – part-time (working 30 or fewer hours per week on average)	
Unemployed – registered unemployed, available and looking for work	
Unemployed – unable to work due to illness	
Unpaid voluntary work	

Student	
Retired	
Housewife/husband	

11. If you have been in education in the last 6 months, approximately how many days have you been off due to health problems? _____________ days

12. If you have been in work in the last 6 months, approximately how many days have you been off due to health problems? _____________ days

13. Please circle the appropriate answer:
The impact of XP on the household finances (for example on what you can spend on food, clothes, and holidays) has been
Very small Small Moderate Large Very large

14. Approximately how much would you estimate that you / your household has paid in the last 6 months for these items? If you paid for items that were a one-off cost longer than 6 months ago, please record these in the "one-off cost" column ***(take a best and approximate guess, and do not spend too long on this – we realise it is not possible to be completely accurate for this!) :***

	**Approximate cost in last 6 months to you**	**A one-off cost**
Sunscreen creams		
UV protective window films		
Other house or car adaptations to protect against UV		
UV visor		
UV protective clothes		
Glasses		
Vitamin D tablets		
UV meter		

15. Please list any medication that you have taken in the last 6 months, because of your XP

**Medication name and strength**	**Length of time that you took / have taken the medication for out of the last 6 months**
	___ months ___ weeks ___ days
	___ months ___ weeks ___ days
	___ months ___ weeks ___ days
	___ months ___ weeks ___ days

**16. What is the date today? (day/month/year)**	

THANK YOU VERY MUCH FOR COMPLETING OUR QUESTIONNAIRE.
