# Supplementary material for: Evaluation of a personalised adherence intervention to improve photoprotection in adults with Xeroderma Pigmentosum (XP): protocol for the trial of XPAND
Source: BMJ Open. 2019 Jul 17;9(7):e028577. doi: 10.1136/bmjopen-2018-028577 (PMC6661555; doi:10.1136/bmjopen-2018-028577)
Supplement: Author's manuscript [file bmjopen-2018-028577.draft_revisions.pdf]

BMJ Open is committed to open peer review. As part of this commitment we make the peer review history of every article we publish publicly available.

When an article is published we post the peer reviewers' comments and the authors' responses online. We also post the versions of the paper that were used during peer review. These are the versions that the peer review comments apply to.

The versions of the paper that follow are the versions that were submitted during the peer review process. They are not the versions of record or the final published versions. They should not be cited or distributed as the published version of this manuscript.

BMJ Open is an open access journal and the full, final, typeset and author-corrected version of record of the manuscript is available on our site with no access controls, subscription charges or pay-per-view fees (http://bmjopen.bmj.com).

If you have any questions on BMJ Open's open peer review process please email info.bmjopen@bmj.com

BMJ Open

Evaluation of a personalised adherence intervention to improve photoprotection in adults with Xeroderma Pigmentosum (XP): Protocol for the trial of XPAND

Journal:	*BMJ Open*
Manuscript ID	bmjopen-2018-028577
Article Type:	Protocol
Date Submitted by the Author:	07-Jan-2019
Complete List of Authors:	Walburn, Jessica; King's College London, Institute of Pharmaceutical Science<br>Norton, Sam; King's College London, Psychology Department, Insitute of Psychiatry<br>Sarkany, Robert ; Guy's and St Thomas' NHS Foundation Trust, St John's Institute of Dermatology<br>Sainsbury, Kirby; Newcastle University, Institute of Health and Society<br>Araújo-Soares, Vera; Newcastle University, Institute of Health and Society<br>Morgan, Myfanwy; King's College London, Institute of Pharmaceutical Science<br>Canfield, Martha; King's College London, Psychology Department, Insitute of Psychiatry<br>Foster, Lesley; Guy's and St Thomas' NHS Foundation Trust, St John's Institute of Dermatology<br>Heydenreich, Jakob; Bispebjerg Hospital, Department of Dermatology<br>McCrone, Paul<br>Mander, Adrian; University of Cambridge, MRC Biostatistics Unit, Institute of Public Health<br>Sniehotta, Falko<br>Wulf, Hans Christian; Bispebjerg Hospital, Department of Dermatology<br>Weinman, John; Kings College London, Institute of Pharmaceutical Sciences & Institute of Psychiatry
Keywords:	Adherence, RCT, Photoprotection, Behaviour change, Xeroderma Pigmentosum, Photodermatology < DERMATOLOGY

SCHOLARONE™
Manuscripts

Trial of XPAND: Protocol 07.01.19 Version 4

Evaluation of a personalised adherence intervention to improve photoprotection in adults with Xeroderma Pigmentosum (XP): Protocol for the trial of XPAND

Authors:

Jessica Walburn *[1], Sam Norton [2], Robert Sarkany [3], Kirby Sainsbury [4], Vera Araújo-Soares [4], Myfanwy Morgan[1], Martha Canfield[2], Lesley Foster [3], Jakob Heydenreich[5], Paul McCrone [6], Adrian Mander [7], Falko F Sniehotta [4], Hans Christian Wulf [5], John Weinman [1]

Affiliations:

1. School of Cancer and Pharmaceutical Sciences, Faculty of Life Sciences and Medicine King's College London, UK
2. Health Psychology Section, Institute of Psychiatry, Psychology & Neuroscience, King's College London, UK; Department of Inflammation Biology, Faculty of Life Sciences and Medicine, King's College London, UK
3. National Xeroderma Pigmentosum Service, Guy's and St. Thomas' NHS Foundation Trust, London, UK
4. Institute of Health & Society, Faculty of Medical Sciences, Newcastle University, UK
5. Department of Dermatology D92, Bispebjerg Hospital, Copenhagen, Denmark.
6. Health Service & Population Research, Institute of Psychiatry, Psychology & Neuroscience, King's College London, UK
7. Cambridge Institute of Public Health, Cambridge University, Cambridge, UK

*Corresponding author: Dr Jessica Walburn, Clinical Practice & Medication Use Group,

For peer review only - http://bmjopen.bmj.com/site/about/guidelines.xhtml

School of Cancer and Pharmaceutical Sciences, King's College London, 150 Stamford Street,

London SE1 9NH, UK; email: jessica.2.walburn@kcl.ac.uk;

For peer review only

ABSTRACT

Introduction: Poor adherence to photoprotection for people with Xeroderma Pigmentosum (XP) can be life-threatening. This is the first randomised controlled trial (RCT) to test the efficacy of a personalised adherence intervention (XPAND) to reduce the level of ultraviolet radiation (UVR) reaching the face, by improving photoprotection activities in adults with XP. Methods and analysis: A two-armed parallel groups RCT where we will randomise 24 patients with sub-optimal adherence to either an intervention group receiving XPAND in 2018 or a delayed intervention group who receive XPAND in 2019. XPAND involves seven sessions, one-to-one with a facilitator, using behaviour change techniques and specially designed materials to target barriers to photoprotection. Following baseline assessment in April 2018 (t0) and intervention, the primary outcome will be measured across 21 consecutive days in June and July 2018 (t1). The primary outcome is the average daily UVR dose to the face (D-to-F), calculated by combining objective UVR exposure at the wrist, (measured by a dosimeter) with face photoprotection activities recorded on a daily diary. Secondary outcomes include average daily UVR D-to-F across 21 days in August (t2); psychosocial process variables measured by daily questions (t0, t1, t2) and self-report questionnaires [t0, t1, t2, December 2018 (t3)]. Intervention cost-utility is assessed by service use and personal cost questionnaires (t0, t3). The delayed intervention control arm participants will complete three further assessments in April 2019 (t4) and June-July 2019 (t5), and December 2019 (t6) with dosimetry and daily UVR protection diary completed for 21 days at t4 and t5. A process evaluation of fidelity, acceptability, and mechanisms of change will be conducted using mixed-methods.

For peer review only - http://bmjopen.bmj.com/site/about/guidelines.xhtml

Ethics and dissemination: This study tests a novel intervention, which, if successful will be integrated into routine clinical care to improve photoprotection in adults with XP. This work has implications for other photosensitive conditions.

Trial registration number: ClinicalTrials.gov Identifier: NCT03445052

Key words: Adherence; RCT; Photoprotection; Behaviour change; Xeroderma Pigmentosum; Photodermatology

Strengths and limitations of this study

- This is the first RCT to evaluate an adherence intervention designed to improve photoprotection in people diagnosed with XP.
- We have a primary outcome which is clinically relevant.
- We control for seasonal differences in environmental UVR exposure by comparing between groups across the same weeks.
- We include a process evaluation to understand participant views of XPAND which will be important when integrating the intervention into current clinical care.
- Recruitment and retention of participants may be difficult in this extremely rare disease.

For peer review only - http://bmjopen.bmj.com/site/about/guidelines.xhtml

INTRODUCTION

Xeroderma Pigmentosum (XP) is a very rare genetic condition, where the body is unable to repair ultraviolet radiation (UVR)-induced damage to DNA caused by daylight [1]. Any UVR exposure dramatically increases the chances of developing skin and eye cancers (e.g., rates of non-melanoma skin cancer increase 10,000 fold)[2]. Photoprotection is *recommended* for the healthy population and those taking medications which may cause photosensitivity. However, it is *essential* for people with XP. There is currently no cure for this disease and the only way to minimise and delay the cancers is meticulous and absolute photoprotection every day. Photoprotection involves a complex set of behaviours (i.e., application of broad-spectrum SPF50 sunscreen, wearing long sleeves and long trousers or skirts, wide brimmed hat, glasses, face scarf or buff and avoiding UVR exposure by staying indoors). The best way of protecting the face is by wearing a face visor (a legionnaire style hat with a UVR-protective transparent film at the front). Analysis of self-reported photoprotection used by 21 adults with XP in the UK revealed that over half were using 'very poor' or no photoprotection for at least 20% of all outdoor time [3]. This poor photoprotection has life-threatening consequences.

In common with other rare conditions [4], little is known about the psychological and behavioural patient-related factors that have the potential to improve health outcomes in patients with XP. In recognition of this deficit, the National Institute of Health Research (NIHR) agreed to fund a novel programme of research to investigate the psychosocial factors associated with non-adherence to photoprotection in XP (RP-PG-1212-20009: Developing a psychological intervention to improve ultraviolet protection and clinical outcomes in XP). The aims of the grant were two-fold: to identify the drivers of non-

For peer review only - http://bmjopen.bmj.com/site/about/guidelines.xhtml

adherence using complex mixed-methods research (qualitative interviews; cross-sectional survey; n-of-1; diary-dosimeter measures)[5] (known as “Phase One”) and to use this knowledge to design and test an intervention to improve adherence (“Phase Two”). We identified 17 modifiable psychosocial drivers of non-adherence to photoprotection activities from Phase One. These drivers or barriers to optimal photoprotection relate to factors influencing the motivation to protect (e.g., doubts about perceived necessity of photoprotection) and volitional factors limiting the enactment of photoprotection activities, even if motivation is high (e.g., lack of habit). Findings from the mixed-methods research studies are published separately [3 6 7].

Nobody has attempted to systematically develop an adherence intervention to improve photoprotection in XP. However, there is growing support for interventions focused on changing patient beliefs about their illness and treatment, translating into improvements in adherence across a range of chronic conditions [8-10]. This suggests that addressing unhelpful illness and treatment beliefs may be an important treatment target for photoprotection in XP. Furthermore, a recent systematic review found evidence in support of the effectiveness of psychosocial interventions to improve adherence to photoprotective activities among non-XP individuals at elevated risk for melanoma (due to personal or family history [11]).

We designed a personalised intervention titled, *‘XPAND: Enhancing XP Photoprotection Activities – New Directions’*, using Intervention Mapping [12]. Intervention Mapping starts by breaking the desired behaviour and determinants of behaviour into constituent parts, which are mapped to theory and translated to intervention components. Relevant theories to guide the intervention design included the Necessity and Concerns Framework [13], Common Sense Model of Illness Regulation [14], and the Theoretical Domains

For peer review only - http://bmjopen.bmj.com/site/about/guidelines.xhtml

Framework (not a theory itself but it combines the components of social cognition models used to explain behaviour)[15]. A full description of the development of the intervention, the process of personalisation, and the final intervention product will be reported separately.

This paper describes the protocol for a randomised controlled trial (RCT) to test the efficacy of XPAND to lower the dose of UVR reaching the face, by improving adherence to photoprotection. This can be achieved by minimising overall UVR exposure (i.e., time spent outside) and by increasing level of photoprotection worn or applied when outside. The primary objective is to reduce the average daily UVR dose to the face (D-to-F) across 21 days in June to July (2018), immediately after the delivery of the main intervention. Secondary objectives are: to maintain reductions in average daily UVR D-to-F across 21 days in August 2018 (after a booster session has been delivered to the intervention group); to increase and then maintain daily ratings of mood, self-efficacy, goal priority, automaticity and photoprotection activities across the two 21-day periods. Tertiary objectives are to explore intervention-related changes from baseline. A qualitative process evaluation will investigate the acceptability, feasibility, and change mechanisms from the perspective of the participant. We will conduct a cost-utility analysis, which will indicate whether implementation into clinical care is economically viable.

METHODS AND ANALYSIS

Participants and recruitment

People diagnosed with XP and registered at the National XP Service at Guy's and St Thomas' NHS Foundation Trust will be recruited into the trial. Eligible patients will be sent an invitation letter, followed by a telephone call from the research nurse, and those interested will be sent the study information leaflet. Following a second call, if they wish to

For peer review only - http://bmjopen.bmj.com/site/about/guidelines.xhtml

participate, they will give written consent at a home visit. Patients will be reminded that participation in the study is voluntary and that they may withdraw at any time. No payment will be given for participation. Screening and recruitment took place between February-March 2018.

Inclusion criteria

1. A confirmed diagnosis of Xeroderma Pigmentosum (reduced DNA repair activity in DNA fibroblast assay, in a clinical context compatible with a diagnosis of XP, and with confirmation by finding pathogenic mutations in an XP gene on gene sequencing)
2. Aged ≥ 16 years
3. Sub-optimal adherence to photoprotection when outdoors, as identified by the XP clinical team from data held in medical notes, or by the research team from data collected during the Phase One studies:
    a. Score <20 on our Adherence to Photoprotection scale (where a total score of 25 indicates optimal photoprotection) [16]
    b. Using photoprotective clothing combinations for the face that have been assessed by the clinical team as anything other than ‘excellent’ or ‘very good’ anytime outdoors, recorded on the daily UVR protection diary [3]
    c. Having a ‘resistant’ or ‘integrated’ mode of adjustment to photoprotection, as identified in the qualitative analysis [7]

Exclusion criteria

1. Diagnosed with cognitive impairment
2. Non-fluent in English (to enable in-depth discussion with the intervention facilitators)

For peer review only - http://bmjopen.bmj.com/site/about/guidelines.xhtml

3. Diagnosed with current clinical depression or anxiety, as detailed in medical notes, or confirmed after completion of Hospital Anxiety and Depression Scale (HADS)[17] at the baseline home visit

Study design and flowchart

The XPAND trial is a phase-II two-armed parallel group RCT, with a delayed intervention control arm. Participants are randomised to either the intervention group who receive the XPAND intervention in addition to routine care between April and June 2018 or to the control group who receive routine care in 2018 and then XPAND between April and June 2019. The delayed intervention control group is included to maximise information collection about the intervention due to the extremely rare nature of XP. For example, achievable sample sizes limit the ability to test between-group differences for the psychosocial outcomes with acceptable power and the delayed intervention group allows for intervention-related changes from baseline testing, which is less robust but still useful.

Patient flow through the study is described in Figure 1. The baseline assessment takes place in April 2018 (t0). Post-randomisation assessments take place after completion of the intervention in June to July 2018 (t1), after a booster session in August 2018 (t2), and a long-term follow-up in December 2018 (t3). All participants wear a wrist-worn UVR dosimeter continuously from t0-t2 and complete daily UVR protection diaries for 21 consecutive days at each assessment time point. At each assessment, participants additionally complete patient reported outcomes. The t3 follow-up does not involve dosimetry or daily UVR protection diary. In addition, the delayed intervention control arm only complete three further assessments in April 2019 (t4) and June-July 2019 (t5), and December 2019 (t6) with dosimetry and daily UVR protection diary completed for 21 days at t4 and t5.

For peer review only - http://bmjopen.bmj.com/site/about/guidelines.xhtml

A nested qualitative study examining fidelity, acceptability, and mechanisms of change will be conducted in the intervention group after the August assessment period is completed.

[please insert Figure 1 here]

The study is funded by the NIHR Programme Grants for Applied Research scheme (RP-PG-1212-20009) and received ethical approval from the West London & GTAC Research Ethics Committee 17/LO/2110.

Randomisation

Participants will be randomised, using an equal allocation ratio, to receive XPAND immediately (2018) or to the delayed group stratified by burning type to attempt to balance those with an extreme versus normal burning response. Participants who are in the same family will be randomised as a cluster to the same group to avoid contamination. Since all participants will be recruited at the point of randomisation, the trial statistician (SN) will generate a random allocation for all participants. The lead researcher (JWa) will randomly assign (coin toss) group 1 and group 2 to be the intervention or control. To protect the integrity of the randomisation, participants in the immediate intervention group will be asked not to reveal their allocation to those outside their immediate family. Group allocation will be concealed from the XP clinical team who are not part of the research team (excluding the PI) to avoid inadvertent changes to the standard care of these participants during the trial (e.g., greater/lesser discussion of adherence during routine clinical appointments).

Primary outcome

For peer review only - http://bmjopen.bmj.com/site/about/guidelines.xhtml

The primary outcome is the average daily UVR D-to-F (standard erythemal dose; SED), across 21 consecutive days between June and July 2018 (t1), following completion of six out of seven intervention sessions by the intervention group. UVR D-to-F is estimated by combining data from a UVR dosimeter worn on the wrist (SunSaver 3, Bispebjerg Hospital, Copenhagen, Denmark) [18] and the patient's photoprotective activities, self-reported using the daily UVR protection diary. The dosimeter was set to measure UVR levels every five seconds and record the average every 5 minutes, which has been validated as providing reliable measurements of total UVR exposure over the course of a day [19]. The combination of the daily UVR protection diary and dosimeter to estimate UVR D-to-F has been used in our earlier research with this population (Phase One) and demonstrated to function sufficiently well to be the primary outcome in this trial. The paper-based daily UVR protection diary allows participants to record periods of time spent outside (in 15-minute intervals) and the timing of any specific photoprotection activities while outside (i.e., wearing a face visor, hat, hoodie worn-up, glasses, scarf or face buff, or applying sunscreen and lip-block). UVR D-to-F will be calculated using the dose recorded at the wrist weighted by the level of protection provided by the combination facial photoprotection behaviours used [5].

Secondary outcomes

The following outcomes relate to between-group comparisons in 2018 and are recorded on the daily UVR protection diary. UVR-related outcomes also use data from the dosimeter.

a. Average daily UVR D-to-F across 21 consecutive days in August 2018 (t2). This is to investigate the stability of any improvement identified in the primary outcome

For peer review only - http://bmjopen.bmj.com/site/about/guidelines.xhtml

period (June-to-July 2018), as it will be 4-6 weeks after the main intervention and follows session 7 (the booster session).

b. Average daily total UVR exposure during each of the 21-day periods (t1, t2) and the average daily time outside across all daylight hours and restricted to 11-3pm, when UVR levels are at their highest. This data will be gathered from the dosimeter and the UVR protection diary.

c. Average daily proportion of time outside across daylight hours and between 11-3pm with "very good" or "excellent" face photoprotection during each of the 21-day periods (t1, t2). The categorisations relate to the Daily Photoprotection Scale (DPS) [3] developed for the Phase One n-of-1 study in consultation with the UK XP clinical team. Facial photoprotection activities (excluding sunscreen) are ranked according to their relative level of photoprotection. The following combinations are classed as "very good photoprotection": hat, glasses and scarf/buff; hat, glasses, scarf/buff and hoodie. "Excellent photoprotection" is wearing a face visor, which completely covers the face.

d. Average daily proportion of time outside across daylight hours with sunscreen protection, defined as when sunscreen was applied within the previous 3 hours, during each of the 21-day periods (t1, t2).

e. Average daily number of times sunscreen is applied irrespective of time outside during each of the 21-day periods (t1, t2).

f. Average daily measures of mood, automaticity of photoprotection activities, prioritisation of photoprotection compared to other priorities, and level of self-efficacy to manage barriers during the two follow-up periods (t1, t2) will be assessed. The single items have been adapted from ecological momentary

For peer review only - http://bmjopen.bmj.com/site/about/guidelines.xhtml

assessment questions used in our n-of-1 study [3] *(e.g., How much do you agree that UVR protection of your face today was something you did automatically without thinking* 0=strongly disagree – 10=strongly agree) and are included on the UVR protection diary.

Tertiary outcomes

The following outcomes are assessed once, at the start of each 21-day period (t0, t1, t2, t3). Health-related quality of life, psychological well-being, automaticity of photoprotection activities, self-efficacy in the context of barriers, and self-rated photoprotection adherence will be assessed using self-report questionnaires. Data will be combined across t1 and t2 to assess proximal impact of the intervention and t3 will be used to assess stability of impact.

See Table 1 for the schedule of self-reported measures.

a. Health-related quality of life will be assessed using the EQ-5D-5L[20]
b. Emotional well-being will be measured by the Short-form Warwick Edinburgh Mental Well-Being scale (SWEMWBS)[21]
c. Automaticity of photoprotection activities will be assessed using the 4-item Self-Report Behavioural Automaticity Index (SRBAI)[22], adapted to photoprotection. It is a validated subscale of the Self-Report Habit Index [23] which focuses on the automaticity of behaviour. It asks respondents to rate the extent to which they agree with each statement on a seven-point Likert-type scale [e.g., *UVR photoprotection is something I do without thinking* (1= strongly disagree-7= strongly agree)]. The average of responses gives a score between 1 and 7, with higher scores indicating greater automaticity.

For peer review only - http://bmjopen.bmj.com/site/about/guidelines.xhtml

d. Self-efficacy to photoprotect will be measured using a 21-item scale [Photoprotection Self-Efficacy Questionnaire (PhotoSEQ)] developed for this study, as no validated questionnaire specific to photoprotection activities in the presence of barriers, as recommended by Bandura (2006) [24], was identified. Three items ask the respondent to rate their level of confidence that they can carry out a type of photoprotection activity (e.g., shifting timing and/or duration of outdoor activity, photoprotection using clothing, correctly apply sunscreen) on a ten-point scale (0=Not at all – 10=very confident); two items ask separately about confidence to wear photoprotective clothing and apply sunscreen in the presence of nine different barriers in the following 4 weeks *[e.g., How confident are you that you can photoprotect even if/when: unexpected things get in the way*]. Two subscales are calculated, an average self-efficacy score for sunscreen and similarly for photoprotective clothing and shifting time/duration of activity. Higher scores indicate greater self-efficacy.

e. A secondary brief measure of photoprotection activities was developed to assess photoprotection to indicate whether improvements in photoprotection are maintained, when participants will not be completing the daily UVR protection diary at t3. To allow comparison with the diary, it will be completed at all follow-up points. The Brief Photoprotection Adherence Questionnaire (BPAQ) has 5 items and assesses duration of time outdoors, and photoprotection used when outdoors during the previous 7 days. Three items assess how often respondents wore protective clothing (e.g., *When you went outside, how often did you protect your face against UVR using protective clothing?* 0=never – 10=all the time); the number of days sunscreen was applied in the morning (0-7 days); and how often sunscreen was re-applied when outdoors for longer periods (0=not at all – 10=always). Two items ask respondents to estimate average duration of

For peer review only - http://bmjopen.bmj.com/site/about/guidelines.xhtml

time outdoors each day (Never, <30mins, 31mins-1 hour, 2 hours, upwards in hourly intervals to >8 hours) and average duration of time outdoors each day between 11am-3pm when environmental UVR levels are highest. Each item will be analysed separately.

Further exploratory analyses will examine change from baseline incorporating the delayed intervention group allowing for higher power to assess intervention-related changes. These will be undertaken unblinded to group allocation after main efficacy analyses are complete. All assessment materials developed for this study are included in supplementary files.

Table 1. Summary of self-reported measures schedule

	Intervention and control groups 2018				Delayed intervention group only 2019		
	Baseline (t0) (April)	Follow-up (t1) (June-July)	Follow-up(t2) (August)	Follow up (t3) (December)	Follow-up (t4) (April)	Follow-up (t5) (June-July)	Follow-up (t6) (December)
UVR Protection Diary	✓	✓	✓		✓	✓	
EuroQol five dimensions questionnaire (EQ-5D)	✓	✓	✓	✓	✓	✓	✓
Short-form Warwick-Edinburgh Mental Well-Being Scale (SWEMWBS)	✓	✓	✓	✓	✓	✓	✓
Self-Report Behavioural Automaticity Index' (SRBAI)	✓	✓	✓	✓	✓	✓	✓
Photoprotection self-efficacy Questionnaire (PhotoSEQ)	✓	✓	✓	✓	✓	✓	✓

For peer review only - http://bmjopen.bmj.com/site/about/guidelines.xhtml

Brief Photoprotection Adherence questionnaire (BPAQ)	✓	✓	✓	✓	✓	✓	✓
Service Use Questionnaire (SUQ)	✓			✓	✓		✓
Feedback questionnaire (intervention grp only)		✓				✓	

The XPAND intervention

XPAND is composed of a combination of one-to-one sessions and materials purpose-designed to target barriers to photoprotection. It was designed to be delivered by a healthcare professional (HCP) without specialist psychological training (e.g., clinical nurse specialist). In the trial context, facilitators will be two psychologists and a research nurse.

One-to-one sessions

Facilitators will deliver 7x one-to-one sessions to each participant. Further information on the structure of the intervention is available in Figure 2. Each session will include a combination of personalised and generic content. The intervention content and techniques were systematically developed and selected using Intervention Mapping [12]. Core strategies delivered to all participants will include those to increase self-efficacy, self-regulatory skills, and the automaticity of photoprotection. In addition, personalised modules will be delivered, as needed, selected initially based on data collected in Phase One studies and a profiling questionnaire, and iteratively as additional barriers emerge during the sessions. The facilitators will use a communication style consistent with Motivational Interviewing [25]. They will be guided by a manual, including theory-based behaviour change techniques

For peer review only

For peer review only - http://bmjopen.bmj.com/site/about/guidelines.xhtml

(BCTs) mapped to a recognised taxonomy [26] and specific techniques from other therapeutic approaches (e.g., Acceptance and Commitment Therapy) [27].

Intervention materials

Patient-facing materials have been developed and will be provided to participants in session 1 and used throughout the intervention to facilitate discussion, provide real-life examples, and reinforce the concepts and skills discussed during the sessions. These are summarised in Figure 2.

[Please insert Figure 2 here]

Sample size calculations

The target sample size for the study is a total of 24 patients (i.e. 12 per group), which was based on the ability to detect an average daily UVR D-to-F difference of 0.10 SED between the groups during the June to July 2018 assessment period. This difference was considered clinically meaningful and potentially achievable due to the personalised nature of the intervention. From our Phase One study of UVR exposure, the average daily UVR D-to-F for people diagnosed with XP was 0.27 SED (SD=0.14), with a within-person correlation of r_{ICC}=0.31. Thus the anticipated difference relates to an effect size of d=0.73, or just over a one-third reduction in average daily UVR D-to-F. Adjusting for the design effect due to the dependence of assessments within individuals, a sample size of 20 patients with 21 daily observations has 80% power to detect a reduction of 0.10 SED in average daily UVR D-to-F at the 5% significance level. Accounting for attrition of 20%, based on our previous research, the target sample size was set at 24.

Statistical analyses

For peer review only - http://bmjopen.bmj.com/site/about/guidelines.xhtml

The main efficacy analyses will be conducted by the trial statistician (SN) following a pre-specified analysis plan and blind to group allocation. The analysis will follow the intention to treat principle with individuals analysed within the groups to which they were randomised irrespective of whether they received or persisted with the intervention.

The treatment effect on the primary outcome of daily UVR D-to-F over 21 days between June and July 2018 and secondary outcome of daily UVR D-to-F over 21 days in August will be estimated simultaneously using a linear mixed model. Given the skewed distribution of UVR D-to-F, a logarithmic transformation will be applied and robust standard errors will be estimated. A random intercept will account for the repeated assessments of UVR D-to-F within individuals across all 42 days, with an autoregressive error structure to account for correlation between assessments across successive days. In addition to the dummy coded group variable, other covariates in the model include a dummy coded variable for the assessment period (June-July vs August), an assessment period by group interaction, the patients baseline average daily UVR, an indictor variable for the patients propensity to burn, and the background level of UVR recorded by the observatory closest to the patients' house on the day. Data will be summarised as the empirical Bayes estimate of the average daily UVR D-to-F in SEDs for each assessment period for each group.

Treatment effects for average daily levels of mood, self-efficacy, goal priority, automaticity and time outside will be analysed for both assessment periods simultaneously using mixed-effects as described above. Treatment effects for adherence, self-efficacy, automaticity, QoL, and psychological well-being taken at the start of the June and August assessment periods will also be estimated using linear mixed models but will specify an unconstrained error structure. It is not anticipated that transformation will be required to

For peer review only - http://bmjopen.bmj.com/site/about/guidelines.xhtml

account for skew in any of these outcomes; however, robust standard errors will be estimated. Covariates included in the analysis will be the same as above except that baseline levels of the outcomes will be included in place of baseline average daily UVR D-to-F.

Further exploratory analysis using dynamic regression models will evaluate changes in both level and variability in daily UVR exposure over time between March and September, accounting for treatment using a regression discontinuity approach. This is possible as the dosimeter will be worn for the entire period. However, due to patient burden and since the UVR protection diary is only completed for three-week intervals, it will also be necessary to calculate UVR D-to-F using average daily photoprotection during the pre and post diary intervals. This differs from the main analysis where UVR D-to-F is calculated directly based on photoprotection activities recorded on the diary.

Process evaluation

A mixed-methods process evaluation, using qualitative interviews and self-report measures, will explore the acceptability of the intervention, changes in photoprotection activities, and psychosocial mechanisms of change. A brief intervention feedback questionnaire, completed immediately after session 6, will be used to record more proximal perceptions of the main content of intervention. Responses to this questionnaire and the profiling questionnaire (see Figure 2) will be used to guide prompts during these interviews and give insight into psychosocial mechanisms of change.

The feedback questionnaire is adapted from one used to assess a fat reduction intervention [28]. It has 5-items assessing overall perceptions of the programme and its

For peer review only - http://bmjopen.bmj.com/site/about/guidelines.xhtml

components, the impact on photoprotection activities, and whether psychosocial variables have changed as a result of the intervention. The questions assess the extent to which respondents agree with statements about the intervention (e.g., *"Overall the programme was interesting*" 1=strongly disagree – 5=completely agree).

In-depth qualitative interviews will be conducted by a research nurse and health psychologist who were involved in the design and delivery of the intervention. They will not interview participants to whom they delivered the intervention. Interviews will be based on a topic guide and will explore participants' views of and experiences with XPAND, including more practical delivery-related aspects, such as the optimal number of sessions, preference for telephone or skype sessions, and the value of any further booster sessions. All interviews will be audio-recorded, transcribed, and coded using thematic analysis [29 30] in NVivo10.

Fidelity Assurance

A fidelity assessment will be undertaken to examine the extent to which facilitators delivered the key components of the intervention, specified in the intervention manual, during the face-to-face sessions. An independent researcher will apply a fidelity checklist to transcripts of all the session 1s and a random selection of subsequent sessions.

Evaluation of the cost-utility of XPAND

A decision model will be used to assess the cost-effectiveness of the intervention. Decision models allow outcomes and costs associated with alternative care process to be investigated via simulations. Model structures simplify the care process such that specific aspects can be focussed on. Advantages of these models are that they can be adapted to reflect the outcomes and costs that occur in a variety of settings (and therefore aid

For peer review only - http://bmjopen.bmj.com/site/about/guidelines.xhtml

generalisability); they allow evidence to be generated in a time and cost-efficient way; and they enable interventions to be evaluated that may be precluded using trial methods. The structure of the model to be used here will be developed by the health economists in collaboration with the clinical researchers, and will consist of health states that patients may be in over time. Transition between the states will be informed by literature on the progression of the condition, expert opinion, and data collected through the trial. The impact of the intervention on these transitions will be determined through the clinical trial. A health service perspective will be used followed by a societal perspective, which will incorporate broader costs.

Service use will be measured via completion of a Client Service Receipt Inventory (CSRI) [31]. This retrospectively assesses use of primary and secondary healthcare services (including surgical interventions), social care, tests/investigations, and aids and adaptations (see supplementary files for Service Use Questionnaire). The cost of these inputs will be calculated by combining the service use data with appropriate unit cost information. Other impacts of XP include additional financial costs for the patient (e.g., sunscreen); and time lost from work/education by patients (e.g., to attend appointments, receive treatment, manage symptoms). Costs of these effects will be calculated using average wage rates and information on returns to education. Costs will be combined with quality-adjusted life years (QALYs) derived from the EQ-5D-5L. The estimated cost of the intervention will be based on staff time required to deliver it, plus additional training and materials.

Trial management

An independent Trial Steering Committee (TSC) attended by the research team, two independent researchers, and one PPI panel member will meet every 3 months to provide

For peer review only - http://bmjopen.bmj.com/site/about/guidelines.xhtml

oversight of the trial. Trial data is collected after each measurement period by a member of the research team not delivering the intervention to that individual. Data are managed and entered by the research team, not the statisticians analysing the data. Adverse events are defined as events that occur during participation in the trial. All will be recorded [death; life threatening; hospitalisation; self-harm; attendance at A&E; distress (intervention group)] and reported to the TSC, who will make a judgement on the link to the trial and recommend modifications or discontinuing trial as appropriate.

Patient and Public Involvement

Patient and Public Involvement (PPI) has been integral to every step in the development of this protocol. The PPI panel advised on the study design, particularly related to participant burden of completion of the daily UVR protection diary, which informed the decision to limit the follow-up of the delayed intervention control group to a single period of 21 days (June-July 2019, t5). In addition, the panel reviewed all the XPAND intervention participant-facing materials which helped ensure they were appropriate and acceptable. The PPI panel were not involved in the recruitment process. A summary of research findings will be sent to all participants and published papers will be made available.

DISCUSSION

This is the first RCT to test a behaviour change intervention to improve adherence to photoprotection in adults with XP; an ambitious undertaking in a rare disease. It uses a robust methodology, both in the systematic development of the intervention and in the use of a clinically-relevant novel measurement approach to estimate dose of UVR reaching the face. The inclusion of a process evaluation will provide insight into the workings of the

For peer review only - http://bmjopen.bmj.com/site/about/guidelines.xhtml

intervention and the mechanism underlying our trial outcomes, as well as information about the acceptability of XPAND, a key consideration for the implementation of the intervention into routine clinical care. We will also have estimates of the cost-effectiveness of the intervention. Furthermore, over and above the obvious clinical advantages of decreasing the risk of morbidity and mortality associated with skin cancer for the XP population, if the trial is found to be efficacious, it will be of interest to those working in other long-term conditions which require photoprotection and may have significance for prevention of UVR-related skin damage in the healthy population.

ETHICS AND DISSEMINATION

This research has been approved by London – West London & GTAC Research Ethics Committee 17/LO/2110. The findings will be published in peer reviewed journals and presented at national and international scientific conferences.

Trial registration number: ClinicalTrials.gov Identifier: NCT03445052

Trial status

Recruitment completed. Patient involvement in the study will conclude in December 2019.

Competing interests

None declared

Acknowledgements

We would like to acknowledge the valuable role of the XP national clinical team (Hiva Fassihi, Tanya Henshaw, Alan Lehmann, Sally Turner) and the PPI panel (Cathy Coleman, Ben Fowler, Ros Tobin, Sandra Webb) in assisting with development of this protocol. In addition, we would like to acknowledge the support of the NIHR team (Hothan Esmael, Jonathan Gabriel,

For peer review only - http://bmjopen.bmj.com/site/about/guidelines.xhtml

Katherine Horner, Thomas Hutchinson) and Robert Pleass (Dermatology Research Manager Guy's and St. Thomas' NHS Foundation Trust). Many thanks to Federica Picariello for commenting on the final draft.

Contributors

JWa drafted the manuscript with assistance from KS and LF. VAS, MC, JH, PM, AM, MM, SN, KS, RS, FFS, JW, HCW reviewed and commented on the manuscript. All authors were involved in study design. LF, JW and KS are involved in intervention delivery and data collection.

Funding

This article presents independent research funded by the National Institute for Health Research (NIHR) under its Programme Grants for Applied Research scheme (RP-PG-1212-20009). The views expressed are those of the authors and not necessarily those of the NHS, the NIHR or the Department of Health. The trial sponsor is Guy's and St. Thomas' NHS Foundation Trust.

Figure legends

Figure 1. Progression of participants through the study

Figure 2. Structure of the XPAND intervention

Reference

1. Fassihi H, Sethi M, Fawcett H, Wing J, Chandler N, Mohammed S, Craythorne E, Morley AM, Lim R, Turner S: **Deep phenotyping of 89 xeroderma pigmentosum patients reveals unexpected heterogeneity dependent on the precise molecular defect.** *Proc Natl Acad Sci USA* 2016, **113**(9):E1236-E1245. doi: 10.1073/pnas.1519444113
2. Bradford PT, Goldstein AM, Tamura D, Khan SG, Ueda T, Boyle J, Oh K-S, Imoto K, Inui H, Moriwaki S-I et al: **Cancer and neurologic degeneration in xeroderma pigmentosum: Long term follow-up characterises the role of DNA repair**. *J Med Genet* 2011, **48**(3):168-176. doi: 10.1136/jmg.2010.083022
3. Sainsbury K, Vieira R, Walburn J, Sniehotta FF, Sarkany R, Weinman J, Araujo-Soares V: **Understanding and predicting a complex behaviour using n-of-1 methods: Photoprotection in xeroderma pigmentosum**. *Health Psychol* 2018. doi: 10.1037/hea0000673

For peer review only - http://bmjopen.bmj.com/site/about/guidelines.xhtml

4. Cooper V, Clatworthy J. Adherence research summarised: Unique patient challenges and support solutions in rare diseases. 2016 [Retrieved from http://www.altantishealthcare.com/.
5. Walburn J, Sarkany R, Norton S, Foster L, Morgan M, Sainsbury K, Araújo-Soares V, Anderson R, Garrood I, Heydenreich J: **An investigation of the predictors of photoprotection and UVR dose to the face in patients with XP: A protocol using observational mixed methods.** *BMJ Open* 2017, **7**(8):e018364. doi: 10.1136/bmjopen-2017-018364
6. Anderson R, Walburn J, Morgan M. **Experiences of stigma over the lifetime of people with xeroderma pigmentosum: A qualitative interview study in the United Kingdom.** *J Health Psychol* 2017:1359105317714643. doi: 10.1177/1359105317714643
7. Morgan M, Anderson R, Walburn J, et al. **The influence of perceived medical risks and psychosocial concerns on photoprotection behaviours among adults with Xeroderma Pigmentosum: A qualitative interview study in the UK.** *BMJ Open* In Press.
8. Broadbent E, Ellis C, Thomas J, Gamble G, Petrie K: **Further development of an illness perception intervention for myocardial infarction patients: A randomized controlled trial**. *J Psychosom Res* 2009, **67**(1):17-23. doi: 10.1016/j.jpsychores.2008.12.001
9. O'Carroll RE, Chambers JA, Dennis M, Sudlow C, Johnston M**: Improving adherence to medication in stroke survivors: A pilot randomised controlled trial**. *Ann Behav Med* 2013, **46**(3):358-368. doi: 10.1007/s12160-013-9515-5
10. Petrie KJ, Perry K, Broadbent E, Weinman J: **A text message programme designed to modify patients' illness and treatment beliefs improves self-reported adherence to asthma preventer medication**. *Br J Health Psychol* 2012, **17**(1):74-84. doi: 10.1111/j.2044-8287.2011.02033.x
11. Wu YP, Aspinwall LG, Conn BM, et al. **A systematic review of interventions to improve adherence to melanoma preventive behaviors for individuals at elevated risk.** *Prev Med* 2016;88:153-67. 10.1016/j.ypmed.2016.04.010
12. Eldredge LKB, Markham CM, Kok G, Ruiter RA, Parcel GS: **Planning health promotion programs: an intervention mapping approach**: John Wiley & Sons; 2016.
13. Horne R, Chapman SC, Parham R, Freemantle N, Forbes A, Cooper V**: Understanding patients' adherence-related beliefs about medicines prescribed for long-term conditions: A meta-analytic review of the Necessity-Concerns Framework.** *PLoS One* 2013, **8**(12):e80633. doi: 10.1371/journal.pone.0080633
14. Leventhal H, Meyer D, Nerenz D: **The common sense representation of illness danger**. In: *Contributions to medical psychology. Volume 2, edn*. Edited by Rachman S. New York: Pergamon Press; 1980: 7-30.
15. Francis JJ, O'Connor D, Curran J: **Theories of behaviour change synthesised into a set of theoretical groupings: Introducing a thematic series on the theoretical domains framework**. *Implement Sci* 2012, **7**(1):35. doi: 10.1186/1748-5908-7-35
16. Canfield M, Norton S, Walburn J, et al. **Adherence to face photoprotection behaviours in xerederma pigmentosum patients: initial validation of a new measure.** *PsyArXiv* 2018, November 2018. doi: 10.31234/osf.io/6g7ba
17. Zigmond AS, Snaith RP: **The hospital anxiety and depression scale.** *Acta Psychiatr Scand* 1983, **67**(6):361-370. doi: 10.1111/j.1600-0447.1983.tb09716.x

For peer review only - http://bmjopen.bmj.com/site/about/guidelines.xhtml

18. Thieden E, Ågren M, Wulf H: **The wrist is a reliable body site for personal dosimetry of ultraviolet radiation**. *Photodermatol Photoimmunol Photomed* 2000, **16**(2):57-61. doi: 10.1034/j.1600-0781.2000.d01-4.x
19. Thieden E, Ågren M, Wulf H. **Solar UVR exposures of indoor workers in a Working and a Holiday Period assessed by personal dosimeters and sun exposure diaries.** *Photodermatol Photoimmunol Photomed* 2001;17(6):249-55. doi: 10.1111/j.1600-0781.2001.170601.x
20. Herdman M, Gudex C, Lloyd A, Janssen M, Kind P, Parkin D, Bonsel G, Badia X: **Development and preliminary testing of the new five-level version of EQ-5D (EQ-5D-5L).** *Qual Life Res* 2011, **20**(10):1727-1736. doi: 10.1007/s11136-011-9903-x
21. Stewart-Brown S, Tennant A, Tennant R, Platt S, Parkinson J, Weich S: **Internal construct validity of the Warwick-Edinburgh mental well-being scale (WEMWBS): A Rasch analysis using data from the Scottish health education population survey**. *Health Qual Life Outcomes* 2009, **7**(1):15. doi: 10.1186/1477-7525-7-15
22. Gardner B, Abraham C, Lally P, de Bruijn G-J: **Towards parsimony in habit measurement: Testing the convergent and predictive validity of an automaticity subscale of the Self-Report Habit Index**. *Int J Behav Nutr Phys Act* 2012, **9**(1):102. doi: 10.1186/1479-5868-9-102
23. Verplanken B, Orbell S: **Reflections on past behavior: A self-report index of habit strength[1].** *J Appl Soc Psychol* 2003, **33**(6):1313-1330. doi: 10.1111/j.1559-1816.2003.tb01951.x
24. Bandura A: **Guide for constructing self-efficacy scales**. In: *Self-efficacy beliefs of adolescents.* Edited by Pajares, F & Urdan T. Greenwich, CT: Information Age Publishing, Inc; 2006: 307-337.
25. Miller WR, Rollnick S: **Motivational interviewing: Helping people change**: Guilford press; 2012.
26. Michie S, Richardson M, Johnston M, Abraham C, Francis J, Hardeman W, Eccles MP, Cane J, Wood CE: **The behavior change technique taxonomy (v1) of 93 hierarchically clustered techniques: Building an international consensus for the reporting of behavior change interventions**. *Ann Behav Med* 2013, **46**(1):81-95. doi: 10.1007/s12160-013-9486-6
27. Hayes SC, Strosahl KD, Wilson KG: **Acceptance and commitment therapy**: Guilford Press New York; 1999.
28. Vandelanotte C, De Bourdeaudhuij I, Brug J: **Acceptability and feasibility of an interactive computer-tailored fat intake intervention in Belgium**. *Health Promot Int* 2004, **19**(4):463-470. doi: 10.1093/heapro/dah408
29. Braun V, Clarke V. **Using thematic analysis in psychology.** *Qual Res Psychol* 2006;3(2):77-101. doi: 10.1191/1478088706qp063oa
30. Braun V, Clarke V, Terry G. **Thematic analysis**. In: Rohleder P, Lyons AC, eds. Qual Res Clin Health Psychol. Basingstoke: Palgrave MacMillan 2014:95-114.
31. Beecham J, Knapp M: **Costing psychiatric interventions**: Gaskell London; 2001.

For peer review only - http://bmjopen.bmj.com/site/about/guidelines.xhtml

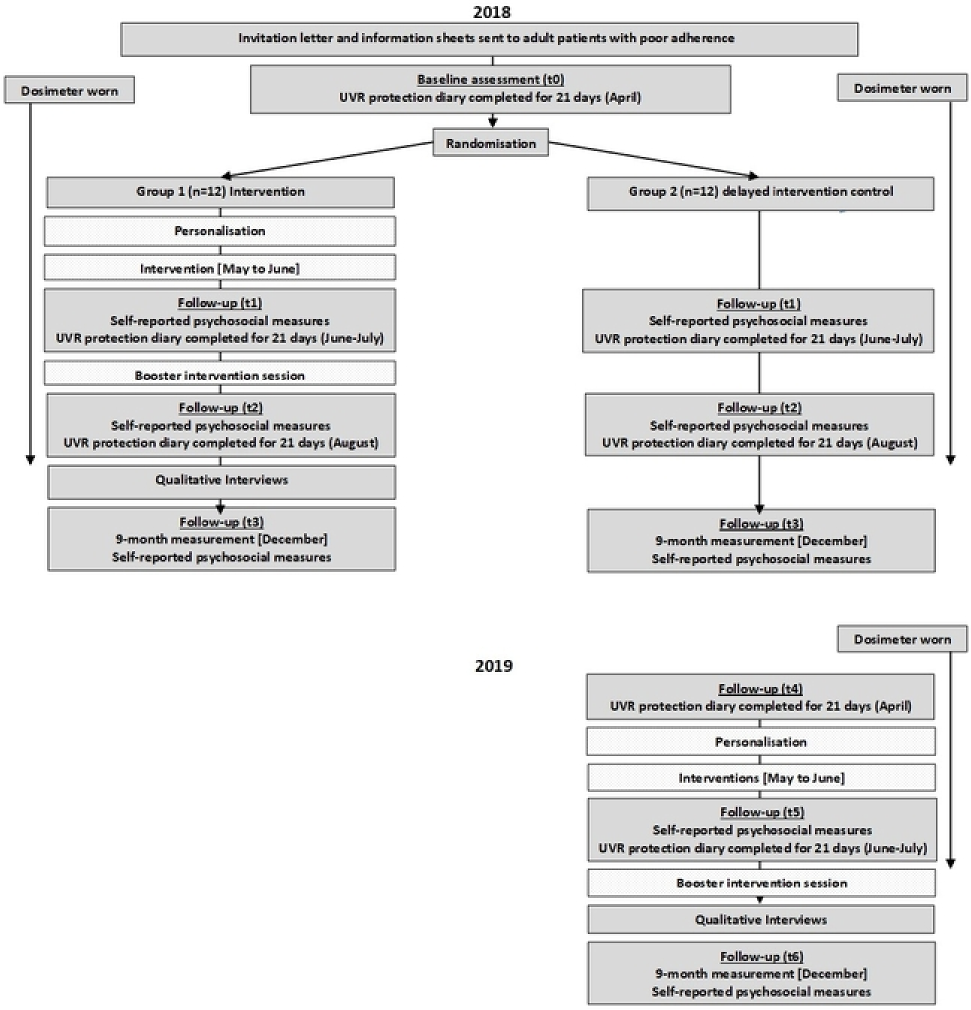

Figure 1. Progression of participants through the study

56x57mm (300 x 300 DPI)

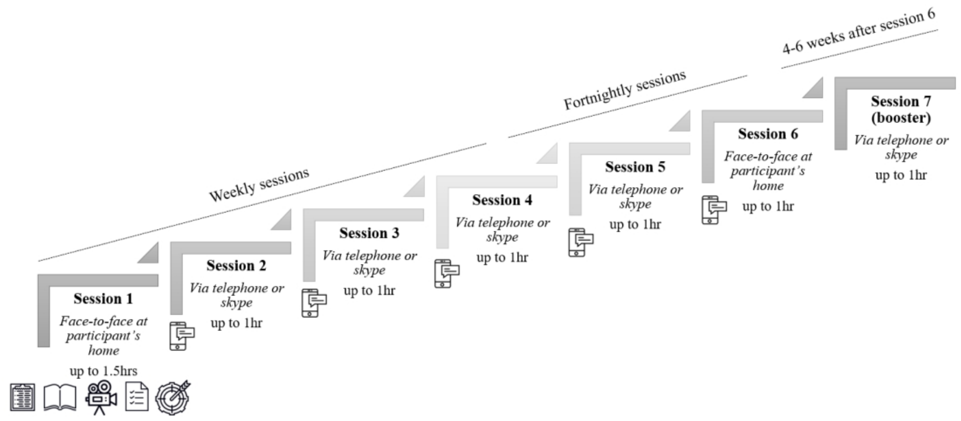

Profiling Questionnaire (PQ) to personalise and make decisions about delivery of intervention content for each individual participant. This 21-items questionnaire assesses drivers associated with suboptimal adherence to photoprotection, identified previously[1]. The items are in the format of the Brief Illness Perception Questionnaire (BIPQ)[2] and use a 0-10 response scale. For example, "How much do you think XP treatment at the clinic can help your skin or eye health?" (0-not at all, 10-very much). The pattern of responses recorded at t0 will be used to ascertain which driver/s appear important for each participant. The PQ will be completed by the intervention group at t1, t2, t3 to explore change in barriers to photoprotection from baseline. The delayed intervention control group will complete it at t4, t5, t6;

XPAND magazine, which targets barriers to photoprotection and has BCTs embedded in the text; participants will be guided to articles relevant to their barriers;

Video of a patient applying the correct amount of sunscreen, set in the context of a morning routine;

Activity sheets for each module;

Photoprotection goal setting tool, the 'UVR dial', which was created to illustrate layering of protective clothing options;

Daily text messages targeting personal barriers and generic behaviour change content; sent for the first 2 weeks, reducing in frequency by session 6.

Figure 2. Structure of the XPAND intervention

XPAND

Your daily UVR diary

For peer review only

Study ID

« 21 DAYS

« 14 DAYS

« 7 DAYS

Your daily UVR diary

People with XP tell us that it can be hard to protect against UVR all the time and we are interested in what works for you.

This booklet has your daily diary sheets to record when you were outside and whether you did anything to protect against UVR each day.

- ★ You only need to record the time you spend outside if it was for **10 minutes or longer**
- ★ "OUTSIDE" is any amount of time longer than 10 minutes you **did not spend in a building**
  "OUTSIDE" includes time spent travelling in cars, buses or trains
- ★ Our grid is split into 15 minute blocks, start your line at the nearest block

We are interested in your views about your UVR protection each day and your general mood. There are four short questions to complete.
Don't take too long thinking about your responses. Your immediate reaction to each question will probably be more accurate than a long thought out response. Please circle the number that best corresponds to your views on that day.

- ★ **If you can't remember exactly or you are unsure give us your best guess!**
- ★ Try to fill out the diary at the end of each day. It will be easier to do when it is fresh in your mind. If you do forget, don't worry! Fill it out as soon as you remember.

? **If you are unsure or have any questions contact Lesley on 07775111823 or email her at Lesley.Foster@gstt.nhs.uk**

Thank you very much for taking the time to complete the daily diary.

KING'S College LONDON

Guy's and St Thomas' NHS NHS Foundation Trust

Daily diary example overleaf

For peer review only - http://bmjopen.bmj.com/site/about/guidelines.xhtml

Your daily UVR diary

Example:

1. **Date (day of week)** 14 May (Saturday)
2. **Did you go outside today?** (Please tick) **YES** ✓ **NO** ☐ If no, skip to question 4
3. **Let us know when you were outside by marking a line on the diary**

	6am	7am	8am	9am	10am	11am	12pm	1pm	2pm	3pm	4pm	5pm	6pm	7pm	8pm	9pm
I was outside																

4. **Let us know if you wore any of these things today by marking a line on the diary**

	6am	7am	8am	9am	10am	11am	12pm	1pm	2pm	3pm	4pm	5pm	6pm	7pm	8pm	9pm
Hat																
Glasses																
Scarf or facebuff																
Hoodie worn up																
Face visor																

5. **If you put sunscreen on your face or used lip sun block tell us each time you did this by ticking the diary**

	6am	7am	8am	9am	10am	11am	12pm	1pm	2pm	3pm	4pm	5pm	6pm	7pm	8pm	9pm
Sun screen	✓				✓				✓							
Lip sunblock	✓															

How would you describe your mood today?

0 1 2 3 4 5 6 7 8 9 (10)

Very negative — Very positive

How important was UVR protection of your face today compared to other things you wanted/needed to do?

0 1 2 3 (4) 5 6 7 8 9 10

Not at all — Very important

How much do you agree that UVR protection of your face today was something you did automatically without thinking?

0 1 2 3 4 5 6 (7) 8 9 10

Strongly disagree — Strongly agree

How confident are you that you can protect your face well tomorrow, even if other things get in the way?

0 1 2 3 4 (5) 6 7 8 9 10

Not at all — Very much so

7 DAYS | 14 DAYS | 21 DAYS

Participant study code_______________

XPAND

Enhancing Photoprotection Activities - New Directions

Xeroderma Pigmentosum National Service
2nd Floor, South Wing
St Thomas' Hospital
Westminster Bridge Road
London SE1 7EH

We know people adjust ultraviolet radiation (UVR) protection to fit in with their everyday life. We are interested in how *you* protected against UVR in the last week.

Please circle the number that best corresponds to your views:

Thinking about your UVR protection over the last week

1. When you went outside, how often did you protect your face against UVR using protective clothing?

0	1	2	3	4	5	6	7	8	9	10
never										*all the time*

2. How many days did you apply sunscreen on your face when getting ready in the morning?

0	1	2	3	4	5	6	7
none							*every day of the week*

3. When you went outside for longer periods, how often did you reapply sunscreen on your face?

0	1	2	3	4	5	6	7	8	9	10
not at all										*always*

4. On average, how many hours did you spend outside per day between 7am and 7pm?

a) *Never*
b) *30min or less*
c) *31 min to 1 hour*
d) *2 hours*
e) *3 hours*
f) *4 hours*
g) *5 hours*
h) *6 hours*
i) *7 hours*
j) *8 hour or more*

For peer review only

For peer review only - http://bmjopen.bmj.com/site/about/guidelines.xhtml

Participant study code________________

5. On average, how many hours did you spend outside per day between 11am and 3pm?

a) *Never*

b) *30min or less*

c) *31 min to 1 hour*

d) *2 hours*

e) *3 hours*

f) *4 hours*

THANK YOU VERY MUCH

For peer review only

For peer review only - http://bmjopen.bmj.com/site/about/guidelines.xhtml

Feedback Questionnaire – 10112017 Version 1

Participant study code____________________
IRAS 236877

XPAND
Enhancing Photoprotection Activities - New Directions
Xeroderma Pigmentosum National Service
2nd Floor, South Wing
St Thomas' Hospital
Westminster Bridge Road
London SE1 7EH

Feedback Questionnaire

We are interested in your opinion of the XPAND programme. Your views will contribute to development of the programme for people newly diagnosed with XP. We are interested in your views, there are no right or wrong answers.

Please tick the box to show how much you agree or disagree with the following statements about the XPAND programme.

1. Overall the programme was……..

	1 Strongly disagree	2	3	4	5 Completely agree
Relevant to me					
Interesting					
Helpful					
Easy to understand					
Too long					
Too short					

Comments

For peer review only - http://bmjopen.bmj.com/site/about/guidelines.xhtml

Feedback Questionnaire – 10112017 Version 1

Participant study code___________________

Please give us reasons for your answers and say if there is anything you'd like us to change….

2. How helpful did you find the different parts of the programme?

	1<br>Not at all helpful	2	3	4	5<br>Very helpful
Face to face meeting with XPAND team member					
Telephone or skype calls with XPAND team member					
Printed materials					
Text messages					

Comments<br>Please explain why you found parts helpful or not helpful

For peer review only - http://bmjopen.bmj.com/site/about/guidelines.xhtml

Feedback Questionnaire – 10112017 Version 1

Participant study code___________________

3. Please tick the box to show how much you agree or disagree with the following statements about the XPAND programme?

The programme helped me to improve my UV protection

Strongly disagree	1	2	3	4	5	Completely agree

Comments
Tell us what you are doing differently? If nothing has changed please write “no change”.

4. The programme has made living with XP easier in other ways (e.g. feeling generally more confident or closer to family and friends)

Strongly disagree	1	2	3	4	5	Completely agree

Comments
Could you tell us how? If nothing has changed please write “no change”.

For peer review only

Feedback Questionnaire – 10112017 Version 1

Participant study code___________________

5. Overall the programme has changed……..

	1 Strongly disagree	2	3	4	5 Completely agree
My motivation to protect					
My confidence in managing protection					
My knowledge					
My routines in managing protection					
My ways of coping when life gets in the way of protection					
How my friends and family support me					

Comments<br>Can you tell us how these things have changed? If nothing has changed please write "no change".

For peer review only

Feedback Questionnaire – 10112017 Version 1

Participant study code___________________

6. Would you recommend taking part in XPAND to other people with XP?

Yes ☐ No ☐

Please add any other comments here

Comments

For peer review only

THANK YOU VERY MUCH

For peer review only - http://bmjopen.bmj.com/site/about/guidelines.xhtml

PSEQ VERSION 1 12/11/2017 IRAS 236877

Participant study code_______________

XPAND
Enhancing Photoprotection Activities - New Directions

Xeroderma Pigmentosum National Service
2nd Floor, South Wing
St Thomas' Hospital
Westminster Bridge Road
London SE1 7EH

We are interested in your own personal views about XP and ultraviolet (UVR) protection. There are no right or wrong answers.

Please circle the number that best corresponds to your views:

1. How confident are you that you can lower your overall exposure to daylight by shifting when or how long you do outdoor daily activities?

   0 1 2 3 4 5 6 7 8 9 10
   not at all *very confident*

2. How confident are you that you can wear all the protective clothing you want, to protect yourself when outdoors?

   0 1 2 3 4 5 6 7 8 9 10
   not at all *very confident*

3. How confident are you that you can correctly apply sunscreen so that you have best protection from UVR?

   0 1 2 3 4 5 6 7 8 9 10
   not at all *very confident*

4. Thinking about using clothing (e.g., hat, glasses, scarf or buff, hoodie) to achieve a high level of photoprotection of your face from UVR over the next 4 weeks, how confident are you that you can photoprotect even if/when………

A. unexpected things get in the way

0 1 2 3 4 5 6 7 8 9 10
not at all *very confident*

B. you are somewhere new

0 1 2 3 4 5 6 7 8 9 10
not at all *very confident*

For peer review only - http://bmjopen.bmj.com/site/about/guidelines.xhtml

PSEQ VERSION 1 12/11/2017 IRAS 236877

C. you are in a rush

0 1 2 3 4 5 6 7 8 9 10

not at all *very confident*

D. you are with new people

0 1 2 3 4 5 6 7 8 9 10

not at all *very confident*

E. you don't want to look different

0 1 2 3 4 5 6 7 8 9 10

not at all *very confident*

F. you are feeling low

0 1 2 3 4 5 6 7 8 9 10

not at all *very confident*

G. you are feeling happy

0 1 2 3 4 5 6 7 8 9 10

not at all *very confident*

H. you are pressured not to protect by family or friends

0 1 2 3 4 5 6 7 8 9 10

not at all *very confident*

I. It might be physically uncomfortable (e.g., too hot)

0 1 2 3 4 5 6 7 8 9 10

not at all *very confident*

5. How confident you are that you will correctly apply sunscreen to your face over the next 4 weeks even if/when:

A. unexpected things get in the way

0 1 2 3 4 5 6 7 8 9 10

not at all *very confident*

For peer review only

For peer review only - http://bmjopen.bmj.com/site/about/guidelines.xhtml

PSEQ VERSION 1 12/11/2017 IRAS 236877

B. you are somewhere new

0 1 2 3 4 5 6 7 8 9 10

not at all *very confident*

C. you are in a rush

0 1 2 3 4 5 6 7 8 9 10

not at all *very confident*

D. you are with new people

0 1 2 3 4 5 6 7 8 9 10

not at all *very confident*

E. you don't want to look different

0 1 2 3 4 5 6 7 8 9 10

not at all *very confident*

F. you are feeling low

0 1 2 3 4 5 6 7 8 9 10

not at all *very confident*

G. you are feeling happy

0 1 2 3 4 5 6 7 8 9 10

not at all *very confident*

H. you are pressured not to protect by family or friends

0 1 2 3 4 5 6 7 8 9 10

not at all *very confident*

I. It might be physically uncomfortable (e.g., sticky)

0 1 2 3 4 5 6 7 8 9 10

not at all *very confident*

For peer review only

SUQ VERSION 1 15/11/2017 IRAS 236877

XPAND
Enhancing Photoprotection Activities - New Directions

Service use and Financial costs of XP to you and your family in the last 6 months

Xeroderma Pigmentosum National Service
2nd Floor, South Wing
St Thomas' Hospital
Westminster Bridge Road
London SE1 7EH

For peer review only

These questions are about the services that you have accessed and the financial cost that the XP causes for you and your family. It is fine if you make your best guess for questions where you are not sure of the answer.

1. **<u>In the last 6 months,</u> have you attended the XP clinic at Guy's and St Thomas' hospital? Please complete the following sentence:**

   a) **I have attended the clinic ____ times in the last 6 months**

If you did not attend the XP clinic in the last 6 months, go to question 2.

If you have attended the clinic in the last 6 months:

   b) **How long did the journey from home to the clinic take? _____ hours _____ minutes**

   c) **Did you have to spend the night away from home in order to visit the clinic?**
   Yes, I stayed in hospital
   Yes, but not in hospital
   No

   d) **Which specialists do you recall seeing on your visit(s) to the XP clinic? Please tick yes or no for each specialist**

**Specialist**	**Yes, I saw this person**	**No, I did not see this person**
Dermatologist		
Ophthalmologist		
Neurologist		
Geneticist		
Psychologist		

SUQ VERSION 1 15/11/2017 IRAS 236877

Specialist Nurse		
Plastic Surgeon		

2. Please complete the following sentence:
In the last 6 months, I have been to Accident and Emergency because of my XP ____ times.

3. Please give details of any hospital outpatient appointments you have had in the last 6 months <u>because of your XP. Do not include trips to the XP clinic at Guy's and St Thomas' here.</u>

**Speciality**	**Number of XP related visits in the last 6 months**	**If you paid for this service, roughly how much did it cost per visit?**	**Roughly how long did it take to travel to the appointment?**
Dermatology			___ hours __ minutes
Ophthalmology			___ hours __ minutes
Neurology			___ hours __ minutes
Paediatrics			___ hours __ minutes
Plastic Surgery			___ hours __ minutes
Psychologist			___ hours __ minutes

4. What surgery you have had in the <u>LAST 6 MONTHS</u>? (day or clinic surgery - where you did <u>not</u> stay in hospital overnight)

**Type of surgery**	**Approximate number of surgeries**	**If you paid for this service, approximately how much did it cost per episode of surgery?**	**Roughly how long did it take to travel for the surgery?**
Skin biopsy			___ hours __ minutes
Surgery to the skin			___ hours __ minutes
Surgery to the eyes			___ hours __ minutes
Other XP related surgery<br>What sort of surgery was it?<br>___________________			___ hours __ minutes

For peer review only

SUQ VERSION 1 15/11/2017 IRAS 236877

5. Please complete the following sentence.
In the last 6 months, I have been admitted to hospital and stayed overnight ___ times because of my XP. In this time, I spent a total of ___ nights in hospital.

For peer review only

SUQ VERSION 1 15/11/2017 IRAS 236877

6. In the last 6 months, which of these services have you visited or been visited at home by, because of your XP?

	**Number of appointments at surgery or clinic**	**Number of home visits**	**If you paid, approximately how much did it cost per visit?**	**How long did it take to travel for the appointment each time if it was not at home?**
General practitioner				___ hours __ minutes
Nurse				___ hours __ minutes
Physiotherapist				___ hours __ minutes
Psychologist , Counsellor or Psychotherapist				___ hours __ minutes
‘Alternative’ medicine or ‘complementary’ therapist				___ hours __ minutes
Occupational therapist				___ hours __ minutes
	**Number of appointments at surgery or clinic**	**Number of home visits**	**If you paid, approximately how much did it cost per visit?**	**How long did it take to travel for the appointment each time if it was not at home?**

For peer review only

SUQ VERSION 1 15/11/2017 IRAS 236877

Social worker				___ hours __ minutes
Home help or home care worker				___ hours __ minutes
Self-help or support group				___ hours __ minutes

7. Please list any tests you remember that you have had done in the LAST 6 MONTHS because of your XP

**Service**	**How many times?**	**If you paid, approximately how much did it cost?**
MRI (Magnetic Resonance Image) brain scan		
Audiometry		
Nerve testing (nerve conduction or EMG test)		
Blood test		
Other investigations / tests<br>What was it?<br>………………………………………………………		

For peer review only

SUQ VERSION 1 15/11/2017 IRAS 236877

8. In the last 6 months, have you received help from friends or relatives with any of the following tasks, as a result of your XP?

Type of help	Please Circle	Approximate number of hours they help you in a week
Child Care<br>(circle 'No' if you have no children)	**No Yes**	
Personal care<br>*(for example washing, dressing)*	**No Yes**	
Help in the house<br>(for example cooking or cleaning)	**No Yes**	
Help outside the house<br>(for example shopping or transport)	**No Yes**	
Going with you to medical appointment	**No Yes**	

9. If you answered yes to any help in the previous question, have your helper(s) taken any time out of work or education to help you as a result of your XP?
If there are part-days to consider, assume that 1 day = 8 hours.
______________ days taken off from work in the last 6 months
______________ days taken off from education in the last 6 months

10. What is your employment status today? Please tick the one that best describes your situation

Paid employment - full-time	
Paid employment – part-time (working 30 or fewer hours per week on average)	
Unemployed – registered unemployed, available and looking for work	
Unemployed – unable to work due to illness	
Unpaid voluntary work	

For peer review only

SUQ VERSION 1 15/11/2017 IRAS 236877

Student	
Retired	
Housewife/husband	

11. If you have been in education in the last 6 months, approximately how many days have you been off due to health problems? _____________ days

12. If you have been in work in the last 6 months, approximately how many days have you been off due to health problems? _____________ days

13. Please circle the appropriate answer:
The impact of XP on the household finances (for example on what you can spend on food, clothes, and holidays) has been
Very small Small Moderate Large Very large

14. Approximately how much would you estimate that you / your household has paid in the last 6 months for these items? If you paid for items that were a one-off cost longer than 6 months ago, please record these in the "one-off cost" column *(take a best and approximate guess, and do not spend too long on this – we realise it is not possible to be completely accurate for this!) :*

	**Approximate cost in last 6 months to you**	**A one-off cost**
Sunscreen creams		
UV protective window films		
Other house or car adaptations to protect against UV		
UV visor		
UV protective clothes		
Glasses		
Vitamin D tablets		
UV meter		

For peer review only

SUQ VERSION 1 15/11/2017 IRAS 236877

15. Please list any medication that you have taken in the last 6 months, because of your XP

**Medication name and strength**	**Length of time that you took / have taken the medication for out of the last 6 months**
	___ months ___ weeks ___ days
	___ months ___ weeks ___ days
	___ months ___ weeks ___ days
	___ months ___ weeks ___ days

**16. What is the date today? (day/month/year)**	

THANK YOU VERY MUCH FOR COMPLETING OUR QUESTIONNAIRE.

For peer review only

Participant study code________________
IRAS 236877 Profile tool version 3 12/11/2017

XPAND

Enhancing Photoprotection Activities - New Directions

Xeroderma Pigmentosum National Service
2nd Floor, South Wing
St Thomas' Hospital
Westminster Bridge Road
London SE1 7EH

We are interested in your own personal views about XP and ultraviolet (UVR) protection. There are no right or wrong answers.

Answering these questions will help us tailor the XP and programme to your needs.

When we say UVR protection we mean anything you do to protect your face from UVR (e.g. sunscreen, clothing, staying indoors).

It will take about 5 minutes to complete.

Please circle the number that best corresponds to your views:

1. How much do you think UVR protection can help your skin or eye health?

   0 1 2 3 4 5 6 7 8 9 10
   not at all ... *very much*

2. How much do you think XP treatment at the clinic can help your skin or eye health?

   0 1 2 3 4 5 6 7 8 9 10
   not at all ... *very much*

3. How well do you think your typical level of protection protects you from UVR when you're outdoors?

   0 1 2 3 4 5 6 7 8 9 10
   not at all ... *completely*

4. How much control do you think you have over the way XP affects your skin or eye health?

   0 1 2 3 4 5 6 7 8 9 10
   no control at all ... *extreme amount of control*

5. How necessary do you think it is to protect yourself from UVR when........

   it is cloudy?

   0 1 2 3 4 5 6 7 8 9 10
   not at all ... *essential*

For peer review only - http://bmjopen.bmj.com/site/about/guidelines.xhtml

Participant study code________________
IRAS 236877 Profile tool version 3 12/11/2017

it is winter?<br><br>0 1 2 3 4 5 6 7 8 9 10<br>*not at all* *essential*
6. How necessary do you think it is to protect yourself from UVR if you go outdoors for less than 15 minutes?<br><br>0 1 2 3 4 5 6 7 8 9 10<br>*not at all* *essential*

Your experiences of UVR protection…

7. How important is it compared to other things you want/need to do?<br><br>0 1 2 3 4 5 6 7 8 9 10<br>*not at all* *very important*
8. How much do you feel that protecting well against UVR disrupts your everyday life?<br><br>0 1 2 3 4 5 6 7 8 9 10<br>*not at all* *very much*
9. How confident are you that you can protect against UVR even if other things get in the way?<br><br>0 1 2 3 4 5 6 7 8 9 10<br>*not at all* *very confident*
10. How much do you worry about other people's reactions to the things you have to do to protect against UVR?<br><br>0 1 2 3 4 5 6 7 8 9 10<br>*not at all* *very much*
11. How much does your UVR protection affect you emotionally?<br><br>0 1 2 3 4 5 6 7 8 9 10<br>*not at all* *very much*

Thinking about your health compared to people without XP

12. What do you think are the chances of a person with XP getting skin cancer compared to a person without XP?<br><br>0 1 2 3 4 5 6 7 8 9 10<br>*same chances* *much more chances*
13. How much do believe that you will get skin cancer at some point, no matter what you do?<br><br>0 1 2 3 4 5 6 7 8 9 10<br>*not at all* *very much*

For peer review only

Participant study code________________
IRAS 236877 Profile tool version 3 12/11/2017

Thinking about people around you…..

14. How much support do you receive from the people around you with your UVR protection (clothes, sunscreen, adjusting daily activities)?<br><br>0 1 2 3 4 5 6 7 8 9 10<br>*no support at all* … *a lot of support*
15. We know that support received doesn't always meet people's needs. In your opinion, how useful do you find the support you receive from others?<br><br>0 1 2 3 4 5 6 7 8 9 10<br>*not at all useful* … *extremely useful*
16. How open are you about your UVR protection needs with people you know but don't live with (e.g., friends or colleagues)?<br><br>0 1 2 3 4 5 6 7 8 9 10<br>*not at all* … *very open*

Thinking about protecting your face from UVR when you go outside tomorrow, How much do you agree with these statements?

17. How motivated are you to protect?<br><br>0 1 2 3 4 5 6 7 8 9 10<br>*not at all* … *very motivated*
18. I have made a detailed plan about (when, where and how) I'm going to protect<br><br>0 1 2 3 4 5 6 7 8 9 10<br>*completely disagree* … *totally agree*
19. I have a plan for how I'm going to protect even if things get in the way<br><br>0 1 2 3 4 5 6 7 8 9 10<br>*completely disagree* … *totally agree*

For peer review only - http://bmjopen.bmj.com/site/about/guidelines.xhtml

Participant study code_______________
IRAS 236877 Profile tool version 3 12/11/2017

Thinking about your UVR protection over the last week

20. To do something automatically means that you do it without having to think about it or consciously remind yourself to do it. How much do you agree that UVR protection was something you did automatically?<br><br>0 1 2 3 4 5 6 7 8 9 10<br>*strongly disagree* … *strongly agree*
21. How often did you purposely put UVR protective items in key places (e.g., sunscreen bottle in the car) to remind you to protect?<br><br>0 1 2 3 4 5 6 7 8 9 10<br>*never* … *all the time*
22. How often did you use the weather as a way of deciding what protection you would use?<br><br>0 1 2 3 4 5 6 7 8 9 10<br>*never* … *all the time*

THANK YOU VERY MUCH FOR ANSWERING THESE QUESTIONS

For peer review only

SPIRIT
STANDARD PROTOCOL ITEMS: RECOMMENDATIONS FOR INTERVENTIONAL TRIALS

SPIRIT 2013 Checklist: Recommended items to address in a clinical trial protocol and related documents*

Section/item	Item No	Description	Page No
**Administrative information**			
Title	1	Descriptive title identifying the study design, population, interventions, and, if applicable, trial acronym	1
Trial registration	2a	Trial identifier and registry name. If not yet registered, name of intended registry	4
	2b	All items from the World Health Organization Trial Registration Data Set	n/a
Protocol version	3	Date and version identifier	Header
Funding	4	Sources and types of financial, material, and other support	22
Roles and responsibilities	5a	Names, affiliations, and roles of protocol contributors	1, 22
	5b	Name and contact information for the trial sponsor	22
	5c	Role of study sponsor and funders, if any, in study design; collection, management, analysis, and interpretation of data; writing of the report; and the decision to submit the report for publication, including whether they will have ultimate authority over any of these activities	22
	5d	Composition, roles, and responsibilities of the coordinating centre, steering committee, endpoint adjudication committee, data management team, and other individuals or groups overseeing the trial, if applicable (see Item 21a for data monitoring committee)	20
**Introduction**			
Background and rationale	6a	Description of research question and justification for undertaking the trial, including summary of relevant studies (published and unpublished) examining benefits and harms for each intervention	5-7
	6b	Explanation for choice of comparators	5-7

For peer review only - http://bmjopen.bmj.com/site/about/guidelines.xhtml

Objectives	7	Specific objectives or hypotheses	7
Trial design	8	Description of trial design including type of trial (eg, parallel group, crossover, factorial, single group), allocation ratio, and framework (eg, superiority, equivalence, noninferiority, exploratory)	9
**Methods: Participants, interventions, and outcomes**			
Study setting	9	Description of study settings (eg, community clinic, academic hospital) and list of countries where data will be collected. Reference to where list of study sites can be obtained	7
Eligibility criteria	10	Inclusion and exclusion criteria for participants. If applicable, eligibility criteria for study centres and individuals who will perform the interventions (eg, surgeons, psychotherapists)	8
Interventions	11a	Interventions for each group with sufficient detail to allow replication, including how and when they will be administered	15-16
	11b	Criteria for discontinuing or modifying allocated interventions for a given trial participant (eg, drug dose change in response to harms, participant request, or improving/worsening disease)	20
	11c	Strategies to improve adherence to intervention protocols, and any procedures for monitoring adherence (eg, drug tablet return, laboratory tests)	18
	11d	Relevant concomitant care and interventions that are permitted or prohibited during the trial	9
Outcomes	12	Primary, secondary, and other outcomes, including the specific measurement variable (eg, systolic blood pressure), analysis metric (eg, change from baseline, final value, time to event), method of aggregation (eg, median, proportion), and time point for each outcome. Explanation of the clinical relevance of chosen efficacy and harm outcomes is strongly recommended	10-15
Participant timeline	13	Time schedule of enrolment, interventions (including any run-ins and washouts), assessments, and visits for participants. A schematic diagram is highly recommended (see Figure)	Figures 1, 2. Table 1

For peer review only - http://bmjopen.bmj.com/site/about/guidelines.xhtml

Sample size	14	Estimated number of participants needed to achieve study objectives and how it was determined, including clinical and statistical assumptions supporting any sample size calculations	16-18
Recruitment	15	Strategies for achieving adequate participant enrolment to reach target sample size	7-8
**Methods: Assignment of interventions (for controlled trials)**			
Allocation:			
Sequence generation	16a	Method of generating the allocation sequence (eg, computer-generated random numbers), and list of any factors for stratification. To reduce predictability of a random sequence, details of any planned restriction (eg, blocking) should be provided in a separate document that is unavailable to those who enrol participants or assign interventions	10
Allocation concealmen t mechanism	16b	Mechanism of implementing the allocation sequence (eg, central telephone; sequentially numbered, opaque, sealed envelopes), describing any steps to conceal the sequence until interventions are assigned	10
Implementat ion	16c	Who will generate the allocation sequence, who will enrol participants, and who will assign participants to interventions	10
Blinding (masking)	17a	Who will be blinded after assignment to interventions (eg, trial participants, care providers, outcome assessors, data analysts), and how	10, 16
	17b	If blinded, circumstances under which unblinding is permissible, and procedure for revealing a participant's allocated intervention during the trial	Can be obtained from authors
**Methods: Data collection, management, and analysis**			
Data collection methods	18a	Plans for assessment and collection of outcome, baseline, and other trial data, including any related processes to promote data quality (eg, duplicate measurements, training of assessors) and a description of study instruments (eg, questionnaires, laboratory tests) along with their reliability and validity, if known. Reference to where data collection forms can be found, if not in the protocol	9-15

	18b	Plans to promote participant retention and complete follow-up, including list of any outcome data to be collected for participants who discontinue or deviate from intervention protocols	n/a
Data management	19	Plans for data entry, coding, security, and storage, including any related processes to promote data quality (eg, double data entry; range checks for data values). Reference to where details of data management procedures can be found, if not in the protocol	20-21
Statistical methods	20a	Statistical methods for analysing primary and secondary outcomes. Reference to where other details of the statistical analysis plan can be found, if not in the protocol	16-17
	20b	Methods for any additional analyses (eg, subgroup and adjusted analyses)	16-17
	20c	Definition of analysis population relating to protocol non-adherence (eg, as randomised analysis), and any statistical methods to handle missing data (eg, multiple imputation)	Can be obtained from authors
**Methods: Monitoring**			
Data monitoring	21a	Composition of data monitoring committee (DMC); summary of its role and reporting structure; statement of whether it is independent from the sponsor and competing interests; and reference to where further details about its charter can be found, if not in the protocol. Alternatively, an explanation of why a DMC is not needed	20-21
	21b	Description of any interim analyses and stopping guidelines, including who will have access to these interim results and make the final decision to terminate the trial	n/a
Harms	22	Plans for collecting, assessing, reporting, and managing solicited and spontaneously reported adverse events and other unintended effects of trial interventions or trial conduct	20-21
Auditing	23	Frequency and procedures for auditing trial conduct, if any, and whether the process will be independent from investigators and the sponsor	Can be obtained from authors
**Ethics and dissemination**			

Research ethics approval	24	Plans for seeking research ethics committee/institutional review board (REC/IRB) approval	10
Protocol amendments	25	Plans for communicating important protocol modifications (eg, changes to eligibility criteria, outcomes, analyses) to relevant parties (eg, investigators, REC/IRBs, trial participants, trial registries, journals, regulators)	Can be obtained from authors
Consent or assent	26a	Who will obtain informed consent or assent from potential trial participants or authorised surrogates, and how (see Item 32)	7-8
	26b	Additional consent provisions for collection and use of participant data and biological specimens in ancillary studies, if applicable	n/a
Confidentiality	27	How personal information about potential and enrolled participants will be collected, shared, and maintained in order to protect confidentiality before, during, and after the trial	20-21
Declaration of interests	28	Financial and other competing interests for principal investigators for the overall trial and each study site	22
Access to data	29	Statement of who will have access to the final trial dataset, and disclosure of contractual agreements that limit such access for investigators	Can be obtained from authors
Ancillary and post-trial care	30	Provisions, if any, for ancillary and post-trial care, and for compensation to those who suffer harm from trial participation	n/a
Dissemination policy	31a	Plans for investigators and sponsor to communicate trial results to participants, healthcare professionals, the public, and other relevant groups (eg, via publication, reporting in results databases, or other data sharing arrangements), including any publication restrictions	22
	31b	Authorship eligibility guidelines and any intended use of professional writers	n/a
	31c	Plans, if any, for granting public access to the full protocol, participant-level dataset, and statistical code	n/a
**Appendices**			

For peer review only

Informed consent materials	32	Model consent form and other related documentation given to participants and authorised surrogates	Can be obtained from authors
Biological specimens	33	Plans for collection, laboratory evaluation, and storage of biological specimens for genetic or molecular analysis in the current trial and for future use in ancillary studies, if applicable	n/a

*It is strongly recommended that this checklist be read in conjunction with the SPIRIT 2013 Explanation & Elaboration for important clarification on the items. Amendments to the protocol should be tracked and dated. The SPIRIT checklist is copyrighted by the SPIRIT Group under the Creative Commons "Attribution-NonCommercial-NoDerivs 3.0 Unported" license.

For peer review only

BMJ Open

Evaluation of a personalised adherence intervention to improve photoprotection in adults with Xeroderma Pigmentosum (XP): Protocol for the trial of XPAND

Journal:	*BMJ Open*
Manuscript ID	bmjopen-2018-028577.R1
Article Type:	Protocol
Date Submitted by the Author:	16-Apr-2019

Complete List of Authors:	Walburn, Jessica; King's College London, Institute of Pharmaceutical Science<br>Norton, Sam; King's College London, Psychology Department, Insitute of Psychiatry<br>Sarkany, Robert ; Guy's and St Thomas' NHS Foundation Trust, St John's Institute of Dermatology<br>Sainsbury, Kirby; Newcastle University, Institute of Health and Society<br>Araújo-Soares, Vera; Newcastle University, Institute of Health and Society<br>Morgan, Myfanwy; King's College London, Institute of Pharmaceutical Science<br>Canfield, Martha; King's College London, Psychology Department, Insitute of Psychiatry<br>Foster, Lesley; Guy's and St Thomas' NHS Foundation Trust, St John's Institute of Dermatology<br>Heydenreich, Jakob; Bispebjerg Hospital, Department of Dermatology<br>McCrone, Paul<br>Mander, Adrian; University of Cambridge, MRC Biostatistics Unit, Institute of Public Health<br>Sniehotta, Falko<br>Wulf, Hans Christian; Bispebjerg Hospital, Department of Dermatology<br>Weinman, John; Kings College London, Institute of Pharmaceutical Sciences & Institute of Psychiatry

**Primary Subject Heading**:	Dermatology
Secondary Subject Heading:	Evidence based practice
Keywords:	Adherence, RCT, Photoprotection, Behaviour change, Xeroderma Pigmentosum, Photodermatology < DERMATOLOGY

SCHOLARONE™
Manuscripts

Evaluation of a personalised adherence intervention to improve photoprotection in adults with Xeroderma Pigmentosum (XP): Protocol for the trial of XPAND

Authors:

Jessica Walburn *[1], Sam Norton [2], Robert Sarkany [3], Kirby Sainsbury [4], Vera Araújo-Soares [4], Myfanwy Morgan[1], Martha Canfield[2], Lesley Foster [3], Jakob Heydenreich[5], Paul McCrone [6], Adrian Mander [7], Falko F Sniehotta [4], Hans Christian Wulf [5], John Weinman [1]

Affiliations:

1. School of Cancer and Pharmaceutical Sciences, Faculty of Life Sciences and Medicine King's College London, UK
2. Health Psychology Section, Institute of Psychiatry, Psychology & Neuroscience, King's College London, UK; Department of Inflammation Biology, Faculty of Life Sciences and Medicine, King's College London, UK
3. National Xeroderma Pigmentosum Service, Guy's and St. Thomas' NHS Foundation Trust, London, UK
4. Institute of Health & Society, Faculty of Medical Sciences, Newcastle University, UK
5. Department of Dermatology D92, Bispebjerg Hospital, Copenhagen, Denmark.
6. Health Service & Population Research, Institute of Psychiatry, Psychology & Neuroscience, King's College London, UK
7. Cambridge Institute of Public Health, Cambridge University, Cambridge, UK

*Corresponding author: Dr Jessica Walburn, Clinical Practice & Medication Use Group, School of Cancer and Pharmaceutical Sciences, King's College London, 150 Stamford Street, London SE1 9NH, UK; email: jessica.2.walburn@kcl.ac.uk;

For peer review only - http://bmjopen.bmj.com/site/about/guidelines.xhtml

ABSTRACT

Introduction: Poor adherence to photoprotection for people with Xeroderma Pigmentosum (XP) can be life-threatening. A randomised controlled trial (RCT) is being conducted to test the efficacy of a personalised adherence intervention (XPAND) to reduce the level of ultraviolet radiation (UVR) reaching the face, by improving photoprotection activities in adults with XP. Methods and analysis: A two-armed parallel groups RCT where we will randomise 24 patients with sub-optimal adherence to either an intervention group receiving XPAND in 2018 or a delayed intervention group who receive XPAND in 2019. XPAND involves seven sessions, one-to-one with a facilitator, using behaviour change techniques and specially designed materials to target barriers to photoprotection. Following baseline assessment in April 2018 (t0) and intervention, the primary outcome will be measured across 21 consecutive days in June and July 2018 (t1). The primary outcome is the average daily UVR dose to the face (D-to-F), calculated by combining objective UVR exposure at the wrist, (measured by a dosimeter) with face photoprotection activities recorded on a daily diary. Secondary outcomes include average daily UVR D-to-F across 21 days in August (t2); psychosocial process variables measured by daily questions (t0, t1, t2) and self-report questionnaires [t0, t1, t2, December 2018 (t3)]. Intervention cost-utility is assessed by service use and personal cost questionnaires (t0, t3). The delayed intervention control arm participants will complete three further assessments in April 2019 (t4) and June-July 2019 (t5), and December 2019 (t6) with dosimetry and daily UVR protection diary completed for 21 days at t4 and t5. A process evaluation will be conducted using mixed-methods. Ethics and dissemination: Ethical approval has been received from West London & GTAC REC

For peer review only - http://bmjopen.bmj.com/site/about/guidelines.xhtml

17/LO/2110. Results will be disseminated in peer reviewed journals and at conferences. This study tests a novel intervention, which, if successful will be integrated into routine care.

Trial registration number: ClinicalTrials.gov Identifier: NCT03445052

Key words: Adherence; RCT; Photoprotection; Behaviour change; Xeroderma Pigmentosum; Photodermatology

Strengths and limitations of this study

- To the best of our knowledge, this is the first RCT to evaluate an adherence intervention designed to improve photoprotection in people diagnosed with XP.
- We have a primary outcome which is clinically relevant.
- We control for seasonal differences in environmental UVR exposure by comparing between groups across the same weeks.
- We include a process evaluation to understand participant views of XPAND which will be important when integrating the intervention into current clinical care.
- Recruitment and retention of participants may be difficult in this extremely rare disease.

For peer review only

For peer review only - http://bmjopen.bmj.com/site/about/guidelines.xhtml

INTRODUCTION

Xeroderma Pigmentosum (XP) is a very rare genetic condition, where the body is unable to repair ultraviolet radiation (UVR)-induced damage to DNA caused by daylight [1]. Any UVR exposure dramatically increases the chances of developing skin and eye cancers (e.g., rates of non-melanoma skin cancer increase 10,000 fold)[2]. Photoprotection is *recommended* for the healthy population and those taking medications which may cause photosensitivity. However, it is *essential* for people with XP. There is currently no cure for this disease and the only way to minimise and delay the cancers is meticulous and absolute photoprotection every day. Photoprotection involves a complex set of behaviours (i.e., application of broad-spectrum SPF50 sunscreen, wearing long sleeves and long trousers or skirts, wide brimmed hat, glasses, face scarf or buff and avoiding UVR exposure by staying indoors). The best way of protecting the face is by wearing a face visor (a legionnaire style hat with a UVR-protective transparent film at the front). Analysis of self-reported photoprotection used by 21 adults with XP in the UK revealed that over half were using 'very poor' or no photoprotection for at least 20% of all outdoor time [3]. This poor photoprotection has life-threatening consequences.

In common with other rare conditions [4], little is known about the psychological and behavioural patient-related factors that have the potential to improve health outcomes in patients with XP. In recognition of this deficit, the National Institute of Health Research (NIHR) agreed to fund a novel programme of research to investigate the psychosocial factors associated with non-adherence to photoprotection in XP (RP-PG-1212-20009: Developing a psychological intervention to improve ultraviolet protection and clinical outcomes in XP). The aims of the grant were two-fold: to identify the drivers of non-

For peer review only - http://bmjopen.bmj.com/site/about/guidelines.xhtml

adherence using complex mixed-methods research (qualitative interviews; cross-sectional survey; n-of-1; diary-dosimeter measures)[5] (known as "Phase One") and to use this knowledge to design and test an intervention to improve adherence ("Phase Two"). We identified 17 modifiable psychosocial drivers of non-adherence to photoprotection activities from Phase One. These drivers or barriers to optimal photoprotection relate to factors influencing the motivation to protect (e.g., doubts about perceived necessity of photoprotection) and volitional factors limiting the enactment of photoprotection activities, even if motivation is high (e.g., lack of habit). Findings from the mixed-methods research studies are published separately [3 6 7].

We aimed to systematically develop an adherence intervention to improve photoprotection in XP. There is growing support for interventions focused on changing patient beliefs about their illness and treatment, translating into improvements in adherence across a range of chronic conditions [8-10]. This suggests that addressing unhelpful illness and treatment beliefs may be an important treatment target for photoprotection in XP. Furthermore, a recent systematic review found evidence in support of the effectiveness of psychosocial interventions to improve adherence to photoprotective activities among non-XP individuals at elevated risk for melanoma (due to personal or family history [11]).

We designed a personalised intervention titled, '*XPAND: Enhancing XP Photoprotection Activities – New Directions*', using Intervention Mapping [12]. Intervention Mapping starts by breaking the desired behaviour and determinants of behaviour into constituent parts, which are mapped to theory and translated to intervention components. Relevant theories to guide the intervention design included the Necessity and Concerns Framework [13], Common Sense Model of Illness Regulation [14], and the Theoretical Domains Framework (not a theory itself but it combines the components of social cognition models

For peer review only - http://bmjopen.bmj.com/site/about/guidelines.xhtml

used to explain behaviour)[15]. A full description of the development of the intervention, the process of personalisation, and the final intervention product will be reported separately.

This paper describes the protocol for a randomised controlled trial (RCT) to test the efficacy of XPAND to lower the dose of UVR reaching the face, by improving adherence to photoprotection. This can be achieved by minimising overall UVR exposure (i.e., time spent outside) and by increasing level of photoprotection worn or applied when outside. The primary objective is to reduce the average daily UVR dose to the face (D-to-F) across 21 days in June to July (2018), immediately after the delivery of the main intervention. Secondary objectives are: to maintain reductions in average daily UVR D-to-F across 21 days in August 2018 (after a booster session has been delivered to the intervention group); to increase and then maintain daily ratings of mood, self-efficacy, goal priority, automaticity and photoprotection activities across the two 21-day periods. Tertiary objectives are to explore intervention-related changes from baseline. A qualitative process evaluation will investigate the acceptability, feasibility, and change mechanisms from the perspective of the participant. We will conduct a cost-utility analysis, which will indicate whether implementation into clinical care is economically viable.

METHODS AND ANALYSIS

Participants and recruitment

People diagnosed with XP and registered at the National XP Service at Guy's and St Thomas' NHS Foundation Trust will be recruited into the trial. Eligible patients will be sent an invitation letter, followed by a telephone call from the research nurse, and those interested will be sent the study information leaflet. Following a second call, if they wish to participate, they will give written consent at a home visit. Patients will be reminded that

For peer review only - http://bmjopen.bmj.com/site/about/guidelines.xhtml

participation in the study is voluntary and that they may withdraw at any time. No payment will be given for participation. Screening and recruitment will take place between February-March 2018.

Inclusion criteria

1. A confirmed diagnosis of Xeroderma Pigmentosum (reduced DNA repair activity in DNA fibroblast assay, in a clinical context compatible with a diagnosis of XP, and with confirmation by finding pathogenic mutations in an XP gene on gene sequencing)
2. Aged ≥ 16 years
3. Sub-optimal adherence to photoprotection when outdoors, as identified by the XP clinical team from data held in medical notes, or by the research team from data collected during the Phase One studies:
    a. Score <20 on our Adherence to Photoprotection scale (where a total score of 25 indicates optimal photoprotection) [16]
    b. Using photoprotective clothing combinations for the face that have been assessed by the clinical team as anything other than 'excellent' or 'very good' anytime outdoors, recorded on the daily UVR protection diary [3]
    c. Having a 'resistant' or 'integrated' mode of adjustment to photoprotection, as identified in the qualitative analysis [7]

Exclusion criteria

1. Diagnosed with cognitive impairment (XP or non-XP related) due to potential impact on the efficacy of the intervention and on the participants' experiences of taking part.

For peer review only - http://bmjopen.bmj.com/site/about/guidelines.xhtml

2. Non-fluent in English (to enable in-depth discussion with the intervention facilitators)
3. Diagnosed with current clinical depression or anxiety, as detailed in medical notes, or confirmed after completion of Hospital Anxiety and Depression Scale (HADS)[17] at the baseline home visit

Study design and flowchart

The XPAND trial is a phase-II two-armed parallel group RCT, with a delayed intervention control arm. Participants are randomised to either the intervention group who receive the XPAND intervention in addition to routine care between April and June 2018 or to the control group who receive routine care in 2018 and then XPAND between April and June 2019. The delayed intervention control group is included to maximise information collection about the intervention due to the extremely rare nature of XP. For example, achievable sample sizes limit the ability to test between-group differences for the psychosocial outcomes with acceptable power and the delayed intervention group allows for intervention-related changes from baseline testing, which is less robust but still useful.

Patient flow through the study is described in Figure 1. The baseline assessment takes place in April 2018 (t0). Post-randomisation assessments take place after completion of the intervention in June to July 2018 (t1), after a booster session in August 2018 (t2), and a long-term follow-up in December 2018 (t3). All participants wear a wrist-worn UVR dosimeter continuously from t0-t2 and complete daily UVR protection diaries for 21 consecutive days at each assessment time point. At each assessment, participants additionally complete patient reported outcomes. The t3 follow-up does not involve dosimetry or daily UVR protection diary. In addition, the delayed intervention control arm only complete three

For peer review only - http://bmjopen.bmj.com/site/about/guidelines.xhtml

further assessments in April 2019 (t4) and June-July 2019 (t5), and December 2019 (t6) with dosimetry and daily UVR protection diary completed for 21 days at t4 and t5.

A nested qualitative study examining fidelity, acceptability, and mechanisms of change will be conducted in the intervention group after the August assessment period is completed.

[please insert Figure 1 here]

The study is funded by the NIHR Programme Grants for Applied Research scheme (RP-PG-1212-20009) and received ethical approval from the West London & GTAC Research Ethics Committee 17/LO/2110.

Randomisation

Participants will be randomised, in blocks using an equal allocation ratio, to receive XPAND immediately (2018) or to the delayed group stratified by burning type to attempt to balance those with an extreme (i.e., scoring between 1-3 on the sunburn severity score [18]) versus normal burning response. Participants who are in the same family will be randomised as a cluster to the same group to avoid contamination. Since all participants will be recruited at the point of randomisation, the trial statistician (SN) will generate a random allocation for all participants together as using fixed block sizes to ensure equal allocation to both groups. The lead researcher (JWa) will randomly assign (coin toss) group 1 and group 2 to be the intervention or control, to which the statistician will be masked. To protect the integrity of the randomisation, participants in the immediate intervention group will be asked not to reveal their allocation to those outside their immediate family. Group allocation will be concealed from the XP clinical team who are not part of the research team (excluding the PI)

For peer review only - http://bmjopen.bmj.com/site/about/guidelines.xhtml

to avoid inadvertent changes to the standard care of these participants during the trial (e.g., greater/lesser discussion of adherence during routine clinical appointments).

Primary outcome

The primary outcome is the average daily UVR D-to-F (standard erythemal dose; SED), across 21 consecutive days between June and July 2018 (t1), following completion of six out of seven intervention sessions by the intervention group. UVR D-to-F is estimated by combining data from a UVR dosimeter worn on the wrist (SunSaver 3, Bispebjerg Hospital, Copenhagen, Denmark) [19] and the patient's photoprotective activities, self-reported using the daily UVR protection diary. The dosimeter was set to measure UVR levels every five seconds and record the average every 5 minutes, which has been validated as providing reliable measurements of total UVR exposure over the course of a day [20]. The combination of the daily UVR protection diary and dosimeter to estimate UVR D-to-F has been used in our earlier research with this population (Phase One) and demonstrated to function sufficiently well to be the primary outcome in this trial. The paper-based daily UVR protection diary allows participants to record periods of time spent outside (in 15-minute intervals) and the timing of any specific photoprotection activities while outside (i.e., wearing a face visor, hat, hoodie worn-up, glasses, scarf or face buff, or applying sunscreen and lip-block). UVR D-to-F will be calculated using the dose recorded at the wrist weighted by the level of protection provided by the combination facial photoprotection behaviours used [5].

Secondary outcomes

The following outcomes relate to between-group comparisons in 2018 and are recorded on the daily UVR protection diary. UVR-related outcomes also use data from the dosimeter.

For peer review only - http://bmjopen.bmj.com/site/about/guidelines.xhtml

a. Average daily UVR D-to-F across 21 consecutive days in August 2018 (t2). This is to investigate the stability of any improvement identified in the primary outcome period (June-to-July 2018), as it will be 4-6 weeks after the main intervention and follows session 7 (the booster session).

b. Average daily total UVR exposure during each of the 21-day periods (t1, t2) and the average daily time outside across all daylight hours and restricted to 11-3pm, when UVR levels are at their highest. This data will be gathered from the dosimeter and the UVR protection diary.

c. Average daily proportion of time outside across daylight hours and between 11-3pm with “very good” or “excellent” face photoprotection during each of the 21-day periods (t1, t2). The categorisations relate to the Daily Photoprotection Scale (DPS) [3] developed for the Phase One n-of-1 study in consultation with the UK XP clinical team. Facial photoprotection activities (excluding sunscreen) are ranked according to their relative level of photoprotection. The following combinations are classed as “very good photoprotection”: hat, glasses and scarf/buff; hat, glasses, scarf/buff and hoodie. “Excellent photoprotection” is wearing a face visor, which completely covers the face.

d. Average daily proportion of time outside across daylight hours with sunscreen protection, defined as when sunscreen was applied within the previous 3 hours, during each of the 21-day periods (t1, t2).

e. Average daily number of times sunscreen is applied irrespective of time outside during each of the 21-day periods (t1, t2).

f. Average daily measures of mood, automaticity of photoprotection activities, prioritisation of photoprotection compared to other priorities, and level of self-

For peer review only - http://bmjopen.bmj.com/site/about/guidelines.xhtml

efficacy to manage barriers during the two follow-up periods (t1, t2) will be assessed. The single items have been adapted from ecological momentary assessment questions used in our n-of-1 study [3] *(e.g., How much do you agree that UVR protection of your face today was something you did automatically without thinking* 0=strongly disagree – 10=strongly agree) and are included on the UVR protection diary.

Tertiary outcomes

The following outcomes are assessed once, at the start of each 21-day period (t0, t1, t2, t3). Health-related quality of life (HRQoL), psychological well-being, automaticity of photoprotection activities, self-efficacy in the context of barriers, and self-rated photoprotection adherence will be measured using self-report questionnaires. Data will be combined across t1 and t2 to assess proximal impact of the intervention and t3 will be used to assess stability of impact.

See Table 1 for the schedule of self-reported measures.

a. HRQoL will be assessed using the EQ-5D-5L[21]
b. Emotional well-being will be measured by the Short-form Warwick Edinburgh Mental Well-Being scale (SWEMWBS)[22]
c. Automaticity of photoprotection activities will be assessed using the 4-item Self-Report Behavioural Automaticity Index (SRBAI)[23], adapted to photoprotection. It is a validated subscale of the Self-Report Habit Index [24] which focuses on the automaticity of behaviour. It asks respondents to rate the extent to which they agree with each statement on a seven-point Likert-type scale [e.g., *UVR photoprotection is something I*

For peer review only

do without thinking (1= strongly disagree-7= strongly agree)]. The average of responses gives a score between 1 and 7, with higher scores indicating greater automaticity.

d. Self-efficacy to photoprotect will be measured using a 21-item scale [Photoprotection Self-Efficacy Questionnaire (PhotoSEQ)] developed for this study, as no validated questionnaire specific to photoprotection activities in the presence of barriers, as recommended by Bandura (2006) [25], was identified. Three items ask the respondent to rate their level of confidence that they can carry out a type of photoprotection activity (e.g., shifting timing and/or duration of outdoor activity, photoprotection using clothing, correctly apply sunscreen) on a ten-point scale (0=Not at all – 10=very confident); two items ask separately about confidence to wear photoprotective clothing and apply sunscreen in the presence of nine different barriers in the following 4 weeks *[e.g., How confident are you that you can photoprotect even if/when: unexpected things get in the way*]. Two subscales are calculated, an average self-efficacy score for sunscreen and similarly for photoprotective clothing and shifting time/duration of activity. Higher scores indicate greater self-efficacy.

e. A secondary brief measure of photoprotection activities was developed to assess photoprotection to indicate whether improvements in photoprotection are maintained, when participants will not be completing the daily UVR protection diary at t3. To allow comparison with the diary, it will be completed at all follow-up points. The Brief Photoprotection Adherence Questionnaire (BPAQ) has 5 items and assesses duration of time outdoors, and photoprotection used when outdoors during the previous 7 days. Three items assess different ways of protecting outdoors: how often respondents wore protective clothing (e.g., *When you went outside, how often did you protect your face against UVR using protective clothing?* 0=never – 10=all the time); the number of days

For peer review only - http://bmjopen.bmj.com/site/about/guidelines.xhtml

sunscreen was applied in the morning (0-7 days); and how often sunscreen was re-applied when outdoors for longer periods (0=not at all – 10=always). Two items ask respondents to estimate average duration of time outdoors each day (Never, <30mins, 31mins-1 hour, 2 hours, upwards in hourly intervals to >8 hours) and average duration of time outdoors each day between 11am-3pm when environmental UVR levels are highest. Each item will be analysed separately.

Further exploratory analyses will examine change from baseline incorporating the delayed intervention group allowing for higher power to assess intervention-related changes. These will be undertaken unblinded to group allocation after main efficacy analyses are complete. All assessment materials developed for this study are included in supplementary files.

Table 1. Summary of self-reported measures schedule

	Intervention and control groups 2018				Delayed intervention group only 2019		
	**Baseline (t0) (April)**	**Follow-up (t1) (June-July)**	**Follow-up(t2) (August)**	**Follow up (t3) (December)**	**Follow-up (t4) (April)**	**Follow-up (t5) (June-July)**	**Follow-up (t6) (December)**
UVR Protection Diary	✓	✓	✓		✓	✓	
EuroQol five dimensions questionnaire (EQ-5D)	✓	✓	✓	✓	✓	✓	✓
Short-form Warwick-Edinburgh Mental Well-Being Scale (SWEMWBS)	✓	✓	✓	✓	✓	✓	✓
Self-Report Behavioural	✓	✓	✓	✓	✓	✓	✓

For peer review only - http://bmjopen.bmj.com/site/about/guidelines.xhtml

Automaticity Index' (SRBAI)							
Photoprotection self-efficacy Questionnaire (PhotoSEQ)	✓	✓	✓	✓	✓	✓	✓
Brief Photoprotection Adherence questionnaire (BPAQ)	✓	✓	✓	✓	✓	✓	✓
Service Use Questionnaire (SUQ)	✓			✓	✓		✓
Feedback questionnaire (intervention grp only)		✓				✓	

The XPAND intervention

XPAND is composed of a combination of one-to-one sessions and materials purpose-designed to target barriers to photoprotection. It was designed to be delivered by a healthcare professional (HCP) without specialist psychological training (e.g., clinical nurse specialist). In the trial context, facilitators will be two psychologists and a research nurse.

One-to-one sessions

Facilitators will deliver 7x one-to-one sessions to each participant. Further information on the structure of the intervention is available in Figure 2. Each session will include a combination of personalised and generic content. The intervention content and techniques were systematically developed and selected using Intervention Mapping [12]. Core strategies delivered to all participants will include those to increase self-efficacy, self-regulatory skills, and the automaticity of photoprotection. In addition, personalised modules will be delivered, as needed, selected initially based on data collected in Phase One studies and a profiling questionnaire, and iteratively as additional barriers emerge during the sessions.

For peer review only - http://bmjopen.bmj.com/site/about/guidelines.xhtml

For peer review only

The facilitators will use a communication style consistent with Motivational Interviewing [26]. They will be guided by a manual, including theory-based behaviour change techniques (BCTs) mapped to a recognised taxonomy [27] and specific techniques from other therapeutic approaches (e.g., Acceptance and Commitment Therapy) [28].

Intervention materials

Patient-facing materials have been developed and will be provided to participants in session 1 and used throughout the intervention to facilitate discussion, provide real-life examples, and reinforce the concepts and skills discussed during the sessions. These are summarised in Figure 2.

[Please insert Figure 2 here]

Sample size calculations

The target sample size for the study is a total of 24 patients (i.e. 12 per group), which was based on the ability to detect an average daily UVR D-to-F difference of 0.10 SED between the groups during the June to July 2018 assessment period. This difference was considered clinically meaningful and potentially achievable due to the personalised nature of the intervention. From our Phase One study of UVR exposure, the average daily UVR D-to-F for people diagnosed with XP was 0.27 SED (SD=0.14), with a within-person correlation of r_{ICC}=0.31. Thus the anticipated difference relates to an effect size of d=0.73, or just over a one-third reduction in average daily UVR D-to-F. Adjusting for the design effect due to the dependence of assessments within individuals, a sample size of 20 patients with 21 daily observations has 80% power to detect a reduction of 0.10 SED in average daily UVR D-to-F at the 5% significance level. Accounting for attrition of 20%, based on our previous research, the target sample size was set at 24.

For peer review only - http://bmjopen.bmj.com/site/about/guidelines.xhtml

Statistical analyses

The main efficacy analyses will be conducted by the trial statistician (SN) following a pre-specified analysis plan and blind to group allocation. The analysis will follow the intention to treat principle with individuals analysed within the groups to which they were randomised irrespective of whether they received or persisted with the intervention.

The treatment effect on the primary outcome of daily UVR D-to-F over 21 days between June and July 2018 and secondary outcome of daily UVR D-to-F over 21 days in August will be estimated simultaneously using a linear mixed model. Given the skewed distribution of UVR D-to-F, a logarithmic transformation will be applied and robust standard errors will be estimated. A random intercept will account for the repeated assessments of UVR D-to-F within individuals across all 42 days, with an autoregressive error structure to account for correlation between assessments across successive days. In addition to the dummy coded group variable, other covariates in the model include a dummy coded variable for the assessment period (June-July vs August), an assessment period by group interaction, the patients baseline average daily UVR, an indictor variable for the patients propensity to burn, and the background level of UVR recorded by the observatory closest to the patients' house on the day. Data will be summarised as the empirical Bayes estimate of the average daily UVR D-to-F in SEDs for each assessment period for each group.

This approach allows for missing daily data within each individual under the assumption that data are *missing at random* We will run sensitivity analyses to explore the influence of missing data on the effect estimates, so as to allow for a consideration of the likely plausible range for the treatment effect under a number of *missing not at random* assumptions. For average daily assessments, where these are not available for the full 21 days, this will

For peer review only - http://bmjopen.bmj.com/site/about/guidelines.xhtml

involve imputing missing values under a range of conservative assumptions using the average daily values of the group to which the individual is assigned using a pattern mixture model approach (e.g., average daily level + 0.0SED to 0.5SED).

Treatment effects for average daily levels of mood, self-efficacy, goal priority, automaticity and time outside will be analysed for both assessment periods simultaneously using mixed-effects as described above. Treatment effects for adherence, self-efficacy, automaticity, HRQoL, and psychological well-being taken at the start of the June and August assessment periods will also be estimated using linear mixed models but will specify an unconstrained error structure. It is not anticipated that transformation will be required to account for skew in any of these outcomes; however, robust standard errors will be estimated. Covariates included in the analysis will be the same as above except that baseline levels of the outcomes will be included in place of baseline average daily UVR D-to-F. Scale scores will be calculated using proration to account for missing items within the scale where there is at most one-third of items missing. For example, where scale includes 6 items, at least 4 items must have been completed otherwise the scale score will be set to missing for that individual.

Further exploratory analysis using dynamic regression models will evaluate changes in both level and variability in daily UVR exposure over time between March and September, accounting for treatment using a regression discontinuity approach. This is possible as the dosimeter will be worn for the entire period. However, due to patient burden and since the UVR protection diary is only completed for three-week intervals, it will also be necessary to calculate UVR D-to-F using average daily photoprotection during the pre and post diary

For peer review only - http://bmjopen.bmj.com/site/about/guidelines.xhtml

intervals. This differs from the main analysis where UVR D-to-F is calculated directly based on photoprotection activities recorded on the diary.

Process evaluation

A mixed-methods process evaluation, using qualitative interviews and self-report measures, will explore the acceptability of the intervention, changes in photoprotection activities, and psychosocial mechanisms of change. A brief intervention feedback questionnaire, completed immediately after session 6, will be used to record more proximal perceptions of the main content of intervention. Responses to this questionnaire and the profiling questionnaire (see Figure 2) will be used to guide prompts during these interviews and give insight into psychosocial mechanisms of change.

The feedback questionnaire is adapted from one used to assess a fat reduction intervention [29]. It has 5-items assessing overall perceptions of the programme and its components, the impact on photoprotection activities, and whether psychosocial variables have changed as a result of the intervention. The questions assess the extent to which respondents agree with statements about the intervention (e.g., *"Overall the programme was interesting*" 1=strongly disagree – 5=completely agree).

In-depth qualitative interviews will be conducted by a research nurse and health psychologist who were involved in the design and delivery of the intervention. They will not interview participants to whom they delivered the intervention. Interviews will be based on a topic guide and will explore participants' views of and experiences with XPAND, including more practical delivery-related aspects, such as the optimal number of sessions, preference

For peer review only - http://bmjopen.bmj.com/site/about/guidelines.xhtml

for telephone or skype sessions, and the value of any further booster sessions. All interviews will be audio-recorded, transcribed, and coded using thematic analysis [30 31] in NVivo10.

Fidelity Assurance

A fidelity assessment will be undertaken to examine the extent to which facilitators delivered the key components of the intervention, specified in the intervention manual, during the face-to-face sessions. Independent researchers will apply a fidelity checklist to audio recordings of all session 1s and session 6s, as well as and a random selection of subsequent sessions.

Evaluation of the cost-utility of XPAND

A decision model will be used to assess the cost-effectiveness of the intervention. Decision models allow outcomes and costs associated with alternative care process to be investigated via simulations. Model structures simplify the care process such that specific aspects can be focussed on. Advantages of these models are that they can be adapted to reflect the outcomes and costs that occur in a variety of settings (and therefore aid generalisability); they allow evidence to be generated in a time and cost-efficient way; and they enable interventions to be evaluated that may be precluded using trial methods. The structure of the model to be used here will be developed by the health economists in collaboration with the clinical researchers, and will consist of health states that patients may be in over time. Transition between the states will be informed by literature on the progression of the condition, expert opinion, and data collected through the trial. The impact of the intervention on these transitions will be determined through the clinical trial. A health service perspective will be used followed by a societal perspective, which will incorporate broader costs.

For peer review only - http://bmjopen.bmj.com/site/about/guidelines.xhtml

Service use will be measured via completion of a Client Service Receipt Inventory (CSRI) [32]. This retrospectively assesses use of primary and secondary healthcare services (including surgical interventions), social care, tests/investigations, and aids and adaptations (see supplementary files for Service Use Questionnaire). The cost of these inputs will be calculated by combining the service use data with appropriate unit cost information. Other impacts of XP include additional financial costs for the patient (e.g., sunscreen); and time lost from work/education by patients (e.g., to attend appointments, receive treatment, manage symptoms). Costs of these effects will be calculated using average wage rates and information on returns to education. Costs will be combined with quality-adjusted life years (QALYs) derived from the EQ-5D-5L. The estimated cost of the intervention will be based on staff time required to deliver it, plus additional training and materials.

Trial management

An independent Trial Steering Committee (TSC) attended by the research team, two independent researchers, and one PPI panel member will meet every 3 months to provide oversight of the trial. Trial data is collected after each measurement period by a member of the research team not delivering the intervention to that individual. Data are managed and entered by the research team, not the statisticians analysing the data. Adverse events are defined as events that occur during participation in the trial. All will be recorded [death; life threatening; hospitalisation; self-harm; attendance at A&E; distress (intervention group)] and reported to the TSC, who will make a judgement on the link to the trial and recommend modifications or discontinuing trial as appropriate.

Patient and Public Involvement

For peer review only - http://bmjopen.bmj.com/site/about/guidelines.xhtml

Patient and Public Involvement (PPI) has been integral to every step in the development of this protocol. The PPI panel advised on the study design, particularly related to participant burden of completion of the daily UVR protection diary, which informed the decision to limit the follow-up of the delayed intervention control group to a single period of 21 days (June-July 2019, t5). In addition, the panel reviewed all the XPAND intervention participant-facing materials which helped ensure they were appropriate and acceptable. The PPI panel were not involved in the recruitment process. A summary of research findings will be sent to all participants and published papers will be made available.

DISCUSSION

To the best of our knowledge this is the first RCT to test a behaviour change intervention to improve adherence to photoprotection in adults with XP; an ambitious undertaking in a rare disease. It uses a robust methodology, both in the systematic development of the intervention and in the use of a clinically-relevant novel measurement approach to estimate dose of UVR reaching the face. The inclusion of a process evaluation will provide insight into the workings of the intervention and the mechanism underlying our trial outcomes, as well as information about the acceptability of XPAND, a key consideration for the implementation of the intervention into routine clinical care. We will also have estimates of the cost-effectiveness of the intervention. Furthermore, over and above the obvious clinical advantages of decreasing the risk of morbidity and mortality associated with skin cancer for the XP population, if the trial is found to be efficacious, it will be of interest to those working in other long-term conditions which require photoprotection and may have significance for prevention of UVR-related skin damage in the healthy population.

For peer review only - http://bmjopen.bmj.com/site/about/guidelines.xhtml

ETHICS AND DISSEMINATION

This research has been approved by London – West London & GTAC Research Ethics Committee 17/LO/2110. The findings will be published in peer reviewed journals and presented at national and international scientific conferences.

Trial registration number: ClinicalTrials.gov Identifier: NCT03445052

Trial status

Recruitment completed. Patient involvement in the study will conclude in December 2019.

Competing interests

None declared

Acknowledgements

We would like to acknowledge the valuable role of the XP national clinical team (Hiva Fassihi, Tanya Henshaw, Alan Lehmann, Sally Turner) and the PPI panel (Cathy Coleman, Ben Fowler, Ros Tobin, Sandra Webb) in assisting with development of this protocol. In addition, we would like to acknowledge the support of the NIHR team (Hothan Esmael, Jonathan Gabriel, Katherine Horner, Thomas Hutchinson) and Robert Pleass (Dermatology Research Manager Guy's and St. Thomas' NHS Foundation Trust). Many thanks to Federica Picariello for commenting on the final draft.

Contributors

JWa drafted the manuscript with assistance from KS and LF. VAS, MC, JH, PM, AM, MM, SN, KS, RS, FFS, JW, HCW reviewed and commented on the manuscript. All authors were involved in study design. LF, JW and KS are involved in intervention delivery and data collection.

For peer review only

For peer review only - http://bmjopen.bmj.com/site/about/guidelines.xhtml

Funding

This article presents independent research funded by the National Institute for Health Research (NIHR) under its Programme Grants for Applied Research scheme (RP-PG-1212-20009). The views expressed are those of the authors and not necessarily those of the NHS, the NIHR or the Department of Health. The trial sponsor is Guy's and St. Thomas' NHS Foundation Trust.

Figure legends

Figure 1. Progression of participants through the study

Figure 2. Structure of the XPAND intervention

References

1. Fassihi H, Sethi M, Fawcett H, et al. Deep phenotyping of 89 xeroderma pigmentosum patients reveals unexpected heterogeneity dependent on the precise molecular defect. *Proceedings of the National Academy of Sciences* 2016;113(9):E1236-E45.
2. Bradford PT, Goldstein AM, Tamura D, et al. Cancer and neurologic degeneration in xeroderma pigmentosum: long term follow-up characterises the role of DNA repair. *J Med Genet* 2011;48(3):168-76. doi: 10.1136/jmg.2010.083022
3. Sainsbury K, Vieira R, Walburn J, et al. Understanding and predicting a complex behaviour using n-of-1 methods: Photoprotection in xeroderma pigmentosum. *Health Psychol* 2018
4. Cooper V, Clatworthy J. Adherence research summarised: Unique patient challenges and support solutions in rare diseases. 2016 [Available from: Retrieved from http://www.altantishealthcare.com/.
5. Walburn J, Sarkany R, Norton S, et al. An investigation of the predictors of photoprotection and UVR dose to the face in patients with XP: a protocol using observational mixed methods. *BMJ open* 2017;7(8):e018364.
6. Anderson R, Walburn J, Morgan M. Experiences of stigma over the lifetime of people with xeroderma pigmentosum: A qualitative interview study in the United Kingdom. *J Health Psychol* 2017:1359105317714643.
7. Morgan M, Anderson R, Walburn J, et al. The influence of perceived medical risks and psychosocial concerns on photoprotection behaviours among adults with Xeroderma Pigmentosum: A qualitative interview study in the UK. . *BMJ Open* In Press
8. Broadbent E, Ellis C, Thomas J, et al. Further development of an illness perception intervention for myocardial infarction patients: a randomized controlled trial. *J Psychosom Res* 2009;67(1):17-23.
9. O'Carroll RE, Chambers JA, Dennis M, et al. Improving adherence to medication in stroke survivors: a pilot randomised controlled trial. *Ann Behav Med* 2013;46(3):358-68.

For peer review only - http://bmjopen.bmj.com/site/about/guidelines.xhtml

10. Petrie KJ, Perry K, Broadbent E, et al. A text message programme designed to modify patients' illness and treatment beliefs improves self-reported adherence to asthma preventer medication. *Br J Health Psychol* 2012;17(1):74-84.
11. Wu YP, Aspinwall LG, Conn BM, et al. A systematic review of interventions to improve adherence to melanoma preventive behaviors for individuals at elevated risk. *Prev Med* 2016;88:153-67.
12. Eldredge LKB, Markham CM, Kok G, et al. Planning health promotion programs: an intervention mapping approach: John Wiley & Sons 2016.
13. Horne R, Chapman SC, Parham R, et al. Understanding patients' adherence-related beliefs about medicines prescribed for long-term conditions: a meta-analytic review of the Necessity-Concerns Framework. *PLoS One* 2013;8(12):e80633.
14. Leventhal H, Meyer D, Nerenz D. The common sense representation of illness danger. In: Rachman S, ed. Contributions to medical psychology. New York: Pergamon Press 1980:7-30.
15. Francis JJ, O'Connor D, Curran J. Theories of behaviour change synthesised into a set of theoretical groupings: introducing a thematic series on the theoretical domains framework. *Implementation Science* 2012;7(1):35.
16. Canfield M, Norton S, Walburn J, et al. Adherence to face photoprotection behaviours in xerederma pigmentosum patients: initial validation of a new measure. *PsyArXiv* 2018, November 2018. doi: https://doi.org/10.31234/osf.io/6g7ba
17. Zigmond AS, Snaith RP. The hospital anxiety and depression scale. *Acta Psychiatr Scand* 1983;67(6):361-70.
18. Sethi M, Lehmann A, Fawcett H, et al. Patients with xeroderma pigmentosum complementation groups C, E and V do not have abnormal sunburn reactions. *Br J Dermatol* 2013;169(6):1279-87.
19. Thieden E, Ågren M, Wulf H. The wrist is a reliable body site for personal dosimetry of ultraviolet radiation. *Photodermatol Photoimmunol Photomed* 2000;16(2):57-61.
20. Thieden E, Ågren M, Wulf H. Solar UVR exposures of indoor workers in a Working and a Holiday Period assessed by personal dosimeters and sun exposure diaries. *Photodermatol Photoimmunol Photomed* 2001;17(6):249-55.
21. Herdman M, Gudex C, Lloyd A, et al. Development and preliminary testing of the new five-level version of EQ-5D (EQ-5D-5L). *Qual Life Res* 2011;20(10):1727-36.
22. Stewart-Brown S, Tennant A, Tennant R, et al. Internal construct validity of the Warwick-Edinburgh mental well-being scale (WEMWBS): a Rasch analysis using data from the Scottish health education population survey. *Health and quality of life outcomes* 2009;7(1):15.
23. Gardner B, Abraham C, Lally P, et al. Towards parsimony in habit measurement: Testing the convergent and predictive validity of an automaticity subscale of the Self-Report Habit Index. *International Journal of Behavioral Nutrition and Physical Activity* 2012;9(1):102.
24. Verplanken B, Orbell S. Reflections on Past Behavior: A Self-Report Index of Habit Strength1. *J Appl Soc Psychol* 2003;33(6):1313-30.
25. Bandura A. Guide for constructing self-efficacy scales. *Self-efficacy beliefs of adolescents* 2006;5(307-337)
26. Miller WR, Rollnick S. Motivational interviewing: Helping people change: Guilford press 2012.

For peer review only - http://bmjopen.bmj.com/site/about/guidelines.xhtml

For peer review only

27. Michie S, Richardson M, Johnston M, et al. The behavior change technique taxonomy (v1) of 93 hierarchically clustered techniques: building an international consensus for the reporting of behavior change interventions. *Ann Behav Med* 2013;46(1):81-95.
28. Hayes SC, Strosahl KD, Wilson KG. Acceptance and commitment therapy: Guilford Press New York 1999.
29. Vandelanotte C, De Bourdeaudhuij I, Brug J. Acceptability and feasibility of an interactive computer-tailored fat intake intervention in Belgium. *Health Promot Int* 2004;19(4):463-70.
30. Braun V, Clarke V. Using thematic analysis in psychology. *Qualitative Research in Psychology* 2006;3(2):77-101.
31. Braun V, Clarke V, Terry G. Thematic analysis. In: Rohleder P, Lyons AC, eds. Qual Res Clin Health Psychol. Basingstoke: Palgrave MacMillan 2014:95-114.
32. Beecham J, Knapp M. Costing psychiatric interventions: Gaskell London 2001.

For peer review only

For peer review only - http://bmjopen.bmj.com/site/about/guidelines.xhtml

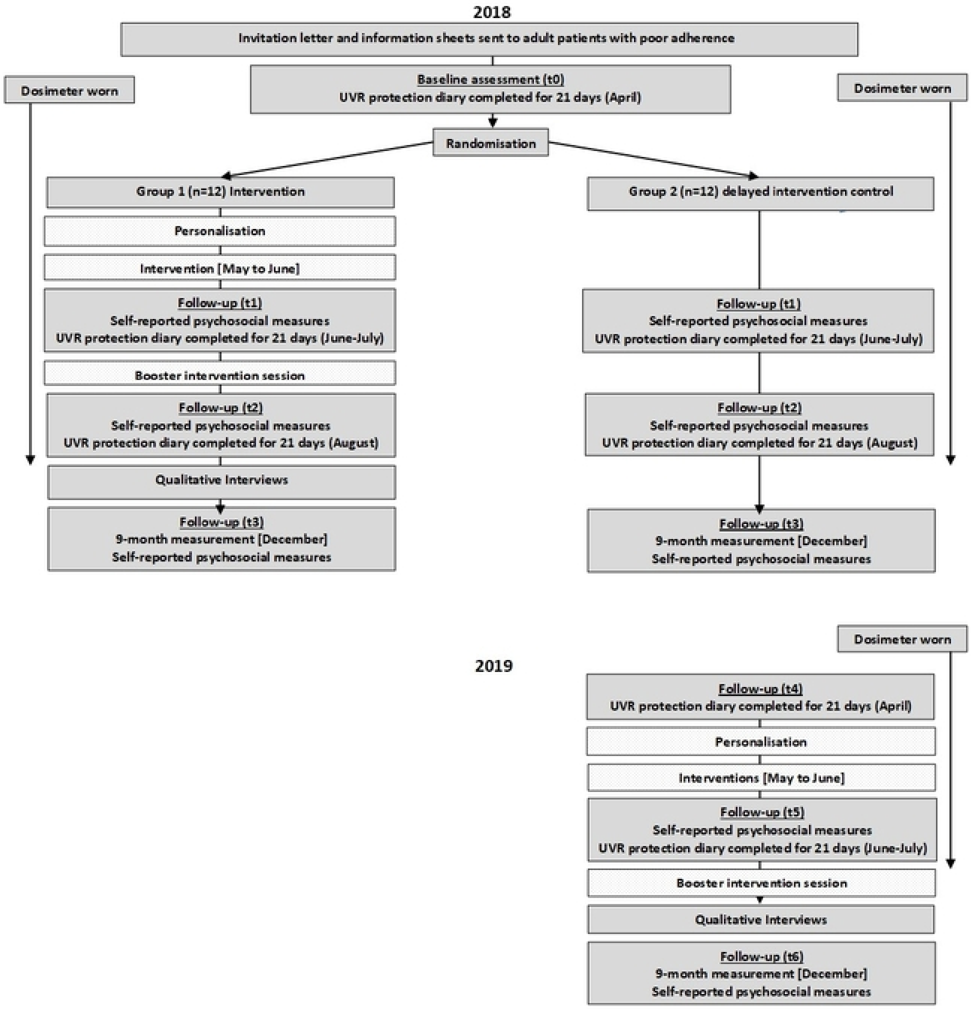

Figure 1. Progression of participants through the study

56x57mm (300 x 300 DPI)

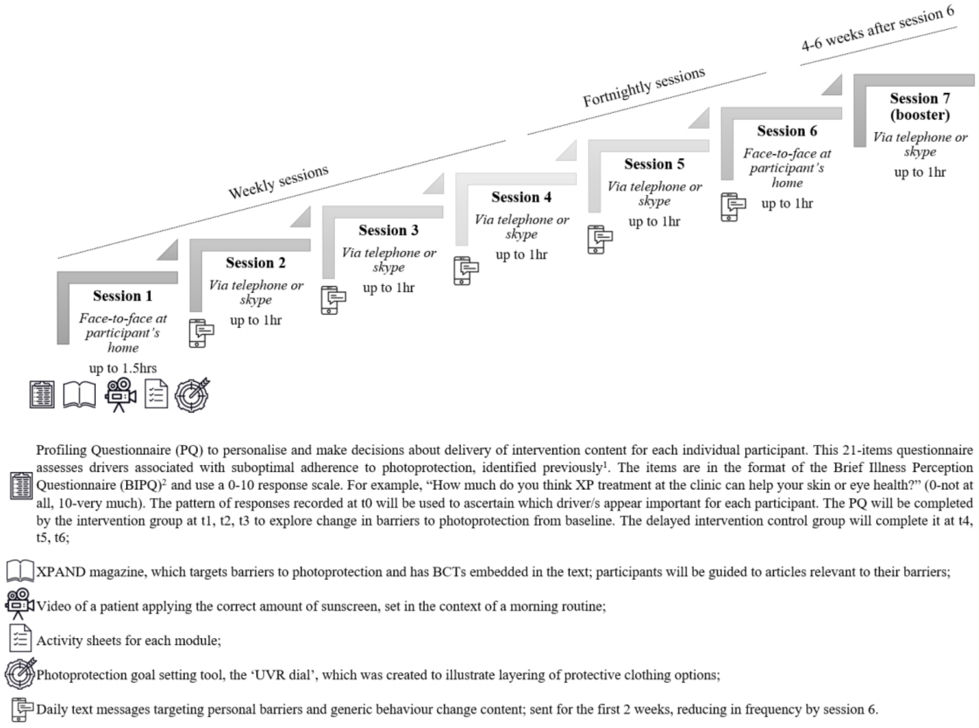

Figure 2. Structure of the XPAND intervention

XPAND

Your daily UVR diary

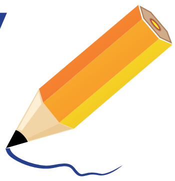

For peer review only

Study ID

« 21 DAYS

« 14 DAYS

« 7 DAYS

Your daily UVR diary

People with XP tell us that it can be hard to protect against UVR all the time and we are interested in what works for you.

This booklet has your daily diary sheets to record when you were outside and whether you did anything to protect against UVR each day.

- ★ You only need to record the time you spend outside if it was for **10 minutes or longer**
- ★ "OUTSIDE" is any amount of time longer than 10 minutes you **did not spend in a building**
  "OUTSIDE" includes time spent travelling in cars, buses or trains
- ★ Our grid is split into 15 minute blocks, start your line at the nearest block

We are interested in your views about your UVR protection each day and your general mood. There are four short questions to complete.
Don't take too long thinking about your responses. Your immediate reaction to each question will probably be more accurate than a long thought out response. Please circle the number that best corresponds to your views on that day.

- ★ **If you can't remember exactly or you are unsure give us your best guess!**
- ★ Try to fill out the diary at the end of each day. It will be easier to do when it is fresh in your mind. If you do forget, don't worry! Fill it out as soon as you remember.

? **If you are unsure or have any questions contact Lesley on 07775111823 or email her at Lesley.Foster@gstt.nhs.uk**

Thank you very much for taking the time to complete the daily diary.

KING'S College LONDON

Guy's and St Thomas' NHS
NHS Foundation Trust

Daily diary example overleaf

For peer review only - http://bmjopen.bmj.com/site/about/guidelines.xhtml

Example:

Your daily UVR diary

1 **Date (day of week)** 14 May (Saturday)

2 **Did you go outside today?** (Please tick) **YES** ✓ **NO** ☐ If no, skip to question 4

3 **Let us know when you were outside by marking a line on the diary**

	6am	7am	8am	9am	10am	11am	12pm	1pm	2pm	3pm	4pm	5pm	6pm	7pm	8pm	9pm
I was outside																

4 **Let us know if you wore any of these things today by marking a line on the diary**

	6am	7am	8am	9am	10am	11am	12pm	1pm	2pm	3pm	4pm	5pm	6pm	7pm	8pm	9pm
Hat																
Glasses																
Scarf or facebuff																
Hoodie worn up																
Face visor																

5 **If you put sunscreen on your face or used lip sun block tell us each time you did this by ticking the diary**

	6am	7am	8am	9am	10am	11am	12pm	1pm	2pm	3pm	4pm	5pm	6pm	7pm	8pm	9pm
Sun screen	✓				✓				✓							
Lip sunblock	✓															

How would you describe your mood today?

0 1 2 3 4 5 6 7 8 9 (10)

Very negative — Very positive

How important was UVR protection of your face today compared to other things you wanted/needed to do?

0 1 2 3 (4) 5 6 7 8 9 10

Not at all — Very important

How much do you agree that UVR protection of your face today was something you did automatically without thinking?

0 1 2 3 4 5 6 (7) 8 9 10

Strongly disagree — Strongly agree

How confident are you that you can protect your face well tomorrow, even if other things get in the way?

0 1 2 3 4 (5) 6 7 8 9 10

Not at all — Very much so

« **7 DAYS** « **14 DAYS** « **21 DAYS**

Participant study code________________

XPAND

Enhancing Photoprotection Activities - New Directions

Xeroderma Pigmentosum National Service
2nd Floor, South Wing
St Thomas' Hospital
Westminster Bridge Road
London SE1 7EH

We know people adjust ultraviolet radiation (UVR) protection to fit in with their everyday life. We are interested in how *you* protected against UVR in the last week.

Please circle the number that best corresponds to your views:

Thinking about your UVR protection over the last week

1. When you went outside, how often did you protect your face against UVR using protective clothing?

   0 *never* 1 2 3 4 5 6 7 8 9 10 *all the time*

2. How many days did you apply sunscreen on your face when getting ready in the morning?

   0 *none* 1 2 3 4 5 6 7 *every day of the week*

3. When you went outside for longer periods, how often did you reapply sunscreen on your face?

   0 *not at all* 1 2 3 4 5 6 7 8 9 10 *always*

4. On average, how many hours did you spend outside per day between 7am and 7pm?

   a) *Never*
   b) *30min or less*
   c) *31 min to 1 hour*
   d) *2 hours*
   e) *3 hours*
   f) *4 hours*
   g) *5 hours*
   h) *6 hours*
   i) *7 hours*
   j) *8 hour or more*

For peer review only

For peer review only - http://bmjopen.bmj.com/site/about/guidelines.xhtml

Participant study code________________

5. On average, how many hours did you spend outside per day between 11am and 3pm?

a) *Never*

b) *30min or less*

c) *31 min to 1 hour*

d) *2 hours*

e) *3 hours*

f) *4 hours*

For peer review only

THANK YOU VERY MUCH

For peer review only - http://bmjopen.bmj.com/site/about/guidelines.xhtml

Feedback Questionnaire – 10112017 Version 1

Participant study code____________________
IRAS 236877

XPAND
Enhancing Photoprotection Activities - New Directions
Xeroderma Pigmentosum National Service
2nd Floor, South Wing
St Thomas' Hospital
Westminster Bridge Road
London SE1 7EH

Feedback Questionnaire

We are interested in your opinion of the XPAND programme. Your views will contribute to development of the programme for people newly diagnosed with XP. We are interested in your views, there are no right or wrong answers.

Please tick the box to show how much you agree or disagree with the following statements about the XPAND programme.

1. Overall the programme was……..

	1 Strongly disagree	2	3	4	5 Completely agree
Relevant to me					
Interesting					
Helpful					
Easy to understand					
Too long					
Too short					

Comments

For peer review only - http://bmjopen.bmj.com/site/about/guidelines.xhtml

Feedback Questionnaire – 10112017 Version 1

Participant study code___________________

Please give us reasons for your answers and say if there is anything you'd like us to change….

2. How helpful did you find the different parts of the programme?

	1 Not at all helpful	2	3	4	5 Very helpful
Face to face meeting with XPAND team member					
Telephone or skype calls with XPAND team member					
Printed materials					
Text messages					

Comments<br>Please explain why you found parts helpful or not helpful

For peer review only - http://bmjopen.bmj.com/site/about/guidelines.xhtml

Feedback Questionnaire – 10112017 Version 1

Participant study code____________________

3. Please tick the box to show how much you agree or disagree with the following statements about the XPAND programme?

The programme helped me to improve my UV protection

Strongly disagree	1	2	3	4	5	Completely agree

Comments<br>Tell us what you are doing differently? If nothing has changed please write "no change".

4. The programme has made living with XP easier in other ways (e.g. feeling generally more confident or closer to family and friends)

Strongly disagree	1	2	3	4	5	Completely agree

Comments<br>Could you tell us how? If nothing has changed please write "no change".

For peer review only - http://bmjopen.bmj.com/site/about/guidelines.xhtml

Feedback Questionnaire – 10112017 Version 1

Participant study code___________________

5. Overall the programme has changed……..

	1 Strongly disagree	2	3	4	5 Completely agree
My motivation to protect					
My confidence in managing protection					
My knowledge					
My routines in managing protection					
My ways of coping when life gets in the way of protection					
How my friends and family support me					

Comments

Can you tell us how these things have changed? If nothing has changed please write "no change".

For peer review only

Feedback Questionnaire – 10112017 Version 1

Participant study code___________________

6. Would you recommend taking part in XPAND to other people with XP?

Yes ☐ No ☐

Please add any other comments here

Comments

THANK YOU VERY MUCH

For peer review only

For peer review only - http://bmjopen.bmj.com/site/about/guidelines.xhtml

PSEQ VERSION 1 12/11/2017 IRAS 236877

Participant study code_______________

XPAND
Enhancing Photoprotection Activities - New Directions

Xeroderma Pigmentosum National Service
2nd Floor, South Wing
St Thomas' Hospital
Westminster Bridge Road
London SE1 7EH

We are interested in your own personal views about XP and ultraviolet (UVR) protection. There are no right or wrong answers.

Please circle the number that best corresponds to your views:

1. How confident are you that you can lower your overall exposure to daylight by shifting when or how long you do outdoor daily activities?

   0 1 2 3 4 5 6 7 8 9 10
   not at all *very confident*

2. How confident are you that you can wear all the protective clothing you want, to protect yourself when outdoors?

   0 1 2 3 4 5 6 7 8 9 10
   not at all *very confident*

3. How confident are you that you can correctly apply sunscreen so that you have best protection from UVR?

   0 1 2 3 4 5 6 7 8 9 10
   not at all *very confident*

4. Thinking about using clothing (e.g., hat, glasses, scarf or buff, hoodie) to achieve a high level of photoprotection of your face from UVR over the next 4 weeks, how confident are you that you can photoprotect even if/when………

A. unexpected things get in the way

0 1 2 3 4 5 6 7 8 9 10
not at all *very confident*

B. you are somewhere new

0 1 2 3 4 5 6 7 8 9 10
not at all *very confident*

For peer review only - http://bmjopen.bmj.com/site/about/guidelines.xhtml

PSEQ VERSION 1 12/11/2017 IRAS 236877

C. you are in a rush

0 1 2 3 4 5 6 7 8 9 10

not at all *very confident*

D. you are with new people

0 1 2 3 4 5 6 7 8 9 10

not at all *very confident*

E. you don't want to look different

0 1 2 3 4 5 6 7 8 9 10

not at all *very confident*

F. you are feeling low

0 1 2 3 4 5 6 7 8 9 10

not at all *very confident*

G. you are feeling happy

0 1 2 3 4 5 6 7 8 9 10

not at all *very confident*

H. you are pressured not to protect by family or friends

0 1 2 3 4 5 6 7 8 9 10

not at all *very confident*

I. It might be physically uncomfortable (e.g., too hot)

0 1 2 3 4 5 6 7 8 9 10

not at all *very confident*

5. How confident you are that you will correctly apply sunscreen to your face over the next 4 weeks even if/when:

A. unexpected things get in the way

0 1 2 3 4 5 6 7 8 9 10

not at all *very confident*

For peer review only - http://bmjopen.bmj.com/site/about/guidelines.xhtml

PSEQ VERSION 1 12/11/2017 IRAS 236877

B. you are somewhere new

0 1 2 3 4 5 6 7 8 9 10

not at all *very confident*

C. you are in a rush

0 1 2 3 4 5 6 7 8 9 10

not at all *very confident*

D. you are with new people

0 1 2 3 4 5 6 7 8 9 10

not at all *very confident*

E. you don’t want to look different

0 1 2 3 4 5 6 7 8 9 10

not at all *very confident*

F. you are feeling low

0 1 2 3 4 5 6 7 8 9 10

not at all *very confident*

G. you are feeling happy

0 1 2 3 4 5 6 7 8 9 10

not at all *very confident*

H. you are pressured not to protect by family or friends

0 1 2 3 4 5 6 7 8 9 10

not at all *very confident*

I. It might be physically uncomfortable (e.g., sticky)

0 1 2 3 4 5 6 7 8 9 10

not at all *very confident*

For peer review only

Participant study code_______________
IRAS 236877 Profile tool version 3 12/11/2017

XPAND
Enhancing Photoprotection Activities - New Directions

Xeroderma Pigmentosum National Service
2nd Floor, South Wing
St Thomas' Hospital
Westminster Bridge Road
London SE1 7EH

We are interested in your own personal views about XP and ultraviolet (UVR) protection. There are no right or wrong answers.

Answering these questions will help us tailor the XP and programme to your needs.

When we say UVR protection we mean anything you do to protect your face from UVR (e.g. sunscreen, clothing, staying indoors).

It will take about 5 minutes to complete.

Please circle the number that best corresponds to your views:

1. How much do you think UVR protection can help your skin or eye health?

0	1	2	3	4	5	6	7	8	9	10
not at all										*very much*

2. How much do you think XP treatment at the clinic can help your skin or eye health?

0	1	2	3	4	5	6	7	8	9	10
not at all										*very much*

3. How well do you think your typical level of protection protects you from UVR when you're outdoors?

0	1	2	3	4	5	6	7	8	9	10
not at all										*completely*

4. How much control do you think you have over the way XP affects your skin or eye health?

0	1	2	3	4	5	6	7	8	9	10
no control at all										*extreme amount of control*

5. How necessary do you think it is to protect yourself from UVR when……..

it is cloudy?

0	1	2	3	4	5	6	7	8	9	10
not at all										*essential*

For peer review only - http://bmjopen.bmj.com/site/about/guidelines.xhtml

Participant study code________________

IRAS 236877 Profile tool version 3 12/11/2017

it is winter?<br><br>0 1 2 3 4 5 6 7 8 9 10<br>*not at all* *essential*
6. How necessary do you think it is to protect yourself from UVR if you go outdoors for less than 15 minutes?<br><br>0 1 2 3 4 5 6 7 8 9 10<br>*not at all* *essential*

Your experiences of UVR protection…

7. How important is it compared to other things you want/need to do?<br><br>0 1 2 3 4 5 6 7 8 9 10<br>*not at all* *very important*
8. How much do you feel that protecting well against UVR disrupts your everyday life?<br><br>0 1 2 3 4 5 6 7 8 9 10<br>*not at all* *very much*
9. How confident are you that you can protect against UVR even if other things get in the way?<br><br>0 1 2 3 4 5 6 7 8 9 10<br>*not at all* *very confident*
10. How much do you worry about other people's reactions to the things you have to do to protect against UVR?<br><br>0 1 2 3 4 5 6 7 8 9 10<br>*not at all* *very much*
11. How much does your UVR protection affect you emotionally?<br><br>0 1 2 3 4 5 6 7 8 9 10<br>*not at all* *very much*

Thinking about your health compared to people without XP

12. What do you think are the chances of a person with XP getting skin cancer compared to a person without XP?<br><br>0 1 2 3 4 5 6 7 8 9 10<br>*same chances* *much more chances*
13. How much do believe that you will get skin cancer at some point, no matter what you do?<br><br>0 1 2 3 4 5 6 7 8 9 10<br>*not at all* *very much*

For peer review only - http://bmjopen.bmj.com/site/about/guidelines.xhtml

Participant study code_______________

IRAS 236877

Profile tool version 3 12/11/2017

Thinking about people around you…..

**14. How much support do you receive from the people around you with your UVR protection (clothes, sunscreen, adjusting daily activities)?**										
0	1	2	3	4	5	6	7	8	9	10
no support at all										*a lot of support*
**15. We know that support received doesn't always meet people's needs. In your opinion, how useful do you find the support you receive from others?**										
0	1	2	3	4	5	6	7	8	9	10
not at all useful										*extremely useful*
**16. How open are you about your UVR protection needs with people you know but don't live with (e.g., friends or colleagues)?**										
0	1	2	3	4	5	6	7	8	9	10
not at all										*very open*

Thinking about protecting your face from UVR when you go outside tomorrow, How much do you agree with these statements?

**17. How motivated are you to protect?**										
0	1	2	3	4	5	6	7	8	9	10
not at all										*very motivated*
**18. I have made a detailed plan about (when, where and how) I'm going to protect**										
0	1	2	3	4	5	6	7	8	9	10
completely disagree										*totally agree*
**19. I have a plan for how I'm going to protect even if things get in the way**										
0	1	2	3	4	5	6	7	8	9	10
completely disagree										*totally agree*

For peer review only

Participant study code________________
IRAS 236877 Profile tool version 3 12/11/2017

Thinking about your UVR protection over the last week

20. To do something automatically means that you do it without having to think about it or consciously remind yourself to do it. How much do you agree that UVR protection was something you did automatically?

0	1	2	3	4	5	6	7	8	9	10
strongly disagree										*strongly agree*

21. How often did you purposely put UVR protective items in key places (e.g., sunscreen bottle in the car) to remind you to protect?

0	1	2	3	4	5	6	7	8	9	10
never										*all the time*

22. How often did you use the weather as a way of deciding what protection you would use?

0	1	2	3	4	5	6	7	8	9	10
never										*all the time*

THANK YOU VERY MUCH FOR ANSWERING THESE QUESTIONS

For peer review only

SUQ VERSION 1 15/11/2017 IRAS 236877

XPAND
Enhancing Photoprotection Activities - New Directions

Service use and Financial costs of XP to you and

your family in the last 6 months

Xeroderma Pigmentosum National Service
2nd Floor, South Wing
St Thomas' Hospital
Westminster Bridge Road
London SE1 7EH

For peer review only

These questions are about the services that you have accessed and the financial cost that the XP causes for you and your family. It is fine if you make your best guess for questions where you are not sure of the answer.

1. In the last 6 months, have you attended the XP clinic at Guy's and St Thomas' hospital? Please complete the following sentence:

a) I have attended the clinic ____ times in the last 6 months

If you did not attend the XP clinic in the last 6 months, go to question 2.

If you have attended the clinic in the last 6 months:

b) How long did the journey from home to the clinic take? _____ hours _____ minutes

c) Did you have to spend the night away from home in order to visit the clinic?
Yes, I stayed in hospital
Yes, but not in hospital
No

d) Which specialists do you recall seeing on your visit(s) to the XP clinic? Please tick yes or no for each specialist

Specialist	Yes, I saw this person	No, I did not see this person
Dermatologist		
Ophthalmologist		
Neurologist		
Geneticist		
Psychologist		

SUQ VERSION 1 15/11/2017 IRAS 236877

Specialist Nurse		
Plastic Surgeon		

2. Please complete the following sentence:
In the last 6 months, I have been to Accident and Emergency because of my XP ____ times.

3. Please give details of any hospital outpatient appointments you have had in the last 6 months <u>because of your XP. Do not include trips to the XP clinic at Guy's and St Thomas' here.</u>

**Speciality**	**Number of XP related visits in the last 6 months**	**If you paid for this service, roughly how much did it cost per visit?**	**Roughly how long did it take to travel to the appointment?**
Dermatology			___ hours __ minutes
Ophthalmology			___ hours __ minutes
Neurology			___ hours __ minutes
Paediatrics			___ hours __ minutes
Plastic Surgery			___ hours __ minutes
Psychologist			___ hours __ minutes

4. What surgery you have had in the <u>LAST 6 MONTHS</u>? (day or clinic surgery - where you did <u>not</u> stay in hospital overnight)

**Type of surgery**	**Approximate number of surgeries**	**If you paid for this service, approximately how much did it cost per episode of surgery?**	**Roughly how long did it take to travel for the surgery?**
Skin biopsy			___ hours __ minutes
Surgery to the skin			___ hours __ minutes
Surgery to the eyes			___ hours __ minutes
Other XP related surgery<br>What sort of surgery was it?<br>____________________			___ hours __ minutes

For peer review only

SUQ VERSION 1 15/11/2017 IRAS 236877

5. Please complete the following sentence.
In the last 6 months, I have been admitted to hospital and stayed overnight ___ times because of my XP. In this time, I spent a total of ___ nights in hospital.

For peer review only

SUQ VERSION 1 15/11/2017 IRAS 236877

6. In the last 6 months, which of these services have you visited or been visited at home by, because of your XP?

	**Number of appointments at surgery or clinic**	**Number of home visits**	**If you paid, approximately how much did it cost per visit?**	**How long did it take to travel for the appointment each time if it was not at home?**
General practitioner				___ hours __ minutes
Nurse				___ hours __ minutes
Physiotherapist				___ hours __ minutes
Psychologist , Counsellor or Psychotherapist				___ hours __ minutes
‘Alternative’ medicine or ‘complementary’ therapist				___ hours __ minutes
Occupational therapist				___ hours __ minutes
	**Number of appointments at surgery or clinic**	**Number of home visits**	**If you paid, approximately how much did it cost per visit?**	**How long did it take to travel for the appointment each time if it was not at home?**

For peer review only

SUQ VERSION 1 15/11/2017 IRAS 236877

Social worker				___ hours __ minutes
Home help or home care worker				___ hours __ minutes
Self-help or support group				___ hours __ minutes

For peer review only

7. Please list any tests you remember that you have had done in the LAST 6 MONTHS because of your XP

**Service**	**How many times?**	**If you paid, approximately how much did it cost?**
MRI (Magnetic Resonance Image) brain scan		
Audiometry		
Nerve testing (nerve conduction or EMG test)		
Blood test		
Other investigations / tests<br><br>What was it?<br><br>……………………………………………………………		

SUQ VERSION 1 15/11/2017 IRAS 236877

8. In the last 6 months, have you received help from friends or relatives with any of the following tasks, as a result of your XP?

Type of help	Please Circle	Approximate number of hours they help you in a week
Child Care<br>(circle 'No' if you have no children)	**No Yes**	
Personal care<br>*(for example washing, dressing)*	**No Yes**	
Help in the house<br>(for example cooking or cleaning)	**No Yes**	
Help outside the house<br>(for example shopping or transport)	**No Yes**	
Going with you to medical appointment	**No Yes**	

9. If you answered yes to any help in the previous question, have your helper(s) taken any time out of work or education to help you as a result of your XP?
If there are part-days to consider, assume that 1 day = 8 hours.
______________ days taken off from work in the last 6 months
______________ days taken off from education in the last 6 months

10. What is your employment status today? Please tick the one that best describes your situation

Paid employment - full-time	
Paid employment – part-time (working 30 or fewer hours per week on average)	
Unemployed – registered unemployed, available and looking for work	
Unemployed – unable to work due to illness	
Unpaid voluntary work	

For peer review only

SUQ VERSION 1 15/11/2017 IRAS 236877

Student	
Retired	
Housewife/husband	

11. If you have been in education in the last 6 months, approximately how many days have you been off due to health problems? _____________ days

12. If you have been in work in the last 6 months, approximately how many days have you been off due to health problems? _____________ days

13. Please circle the appropriate answer:
The impact of XP on the household finances (for example on what you can spend on food, clothes, and holidays) has been
Very small Small Moderate Large Very large

14. Approximately how much would you estimate that you / your household has paid in the last 6 months for these items? If you paid for items that were a one-off cost longer than 6 months ago, please record these in the "one-off cost" column *(take a best and approximate guess, and do not spend too long on this – we realise it is not possible to be completely accurate for this!) :*

	**Approximate cost in last 6 months to you**	**A one-off cost**
Sunscreen creams		
UV protective window films		
Other house or car adaptations to protect against UV		
UV visor		
UV protective clothes		
Glasses		
Vitamin D tablets		
UV meter		

For peer review only

SUQ VERSION 1 15/11/2017 IRAS 236877

15. Please list any medication that you have taken in the last 6 months, because of your XP

Medication name and strength	Length of time that you took / have taken the medication for out of the last 6 months
	___ months ___ weeks ___ days
	___ months ___ weeks ___ days
	___ months ___ weeks ___ days
	___ months ___ weeks ___ days

**16. What is the date today? (day/month/year)**	

THANK YOU VERY MUCH FOR COMPLETING OUR QUESTIONNAIRE.

For peer review only

STANDARD PROTOCOL ITEMS: RECOMMENDATIONS FOR INTERVENTIONAL TRIALS

SPIRIT 2013 Checklist: Recommended items to address in a clinical trial protocol and related documents*

Section/item	Item No	Description	Page No
**Administrative information**			
Title	1	Descriptive title identifying the study design, population, interventions, and, if applicable, trial acronym	1
Trial registration	2a	Trial identifier and registry name. If not yet registered, name of intended registry	4
	2b	All items from the World Health Organization Trial Registration Data Set	n/a
Protocol version	3	Date and version identifier	Header
Funding	4	Sources and types of financial, material, and other support	22
Roles and responsibilities	5a	Names, affiliations, and roles of protocol contributors	1, 22
	5b	Name and contact information for the trial sponsor	22
	5c	Role of study sponsor and funders, if any, in study design; collection, management, analysis, and interpretation of data; writing of the report; and the decision to submit the report for publication, including whether they will have ultimate authority over any of these activities	22
	5d	Composition, roles, and responsibilities of the coordinating centre, steering committee, endpoint adjudication committee, data management team, and other individuals or groups overseeing the trial, if applicable (see Item 21a for data monitoring committee)	20
**Introduction**			
Background and rationale	6a	Description of research question and justification for undertaking the trial, including summary of relevant studies (published and unpublished) examining benefits and harms for each intervention	5-7
	6b	Explanation for choice of comparators	5-7

For peer review only

Objectives	7	Specific objectives or hypotheses	7
Trial design	8	Description of trial design including type of trial (eg, parallel group, crossover, factorial, single group), allocation ratio, and framework (eg, superiority, equivalence, noninferiority, exploratory)	9
**Methods: Participants, interventions, and outcomes**			
Study setting	9	Description of study settings (eg, community clinic, academic hospital) and list of countries where data will be collected. Reference to where list of study sites can be obtained	7
Eligibility criteria	10	Inclusion and exclusion criteria for participants. If applicable, eligibility criteria for study centres and individuals who will perform the interventions (eg, surgeons, psychotherapists)	8
Interventions	11a	Interventions for each group with sufficient detail to allow replication, including how and when they will be administered	15-16
	11b	Criteria for discontinuing or modifying allocated interventions for a given trial participant (eg, drug dose change in response to harms, participant request, or improving/worsening disease)	20
	11c	Strategies to improve adherence to intervention protocols, and any procedures for monitoring adherence (eg, drug tablet return, laboratory tests)	18
	11d	Relevant concomitant care and interventions that are permitted or prohibited during the trial	9
Outcomes	12	Primary, secondary, and other outcomes, including the specific measurement variable (eg, systolic blood pressure), analysis metric (eg, change from baseline, final value, time to event), method of aggregation (eg, median, proportion), and time point for each outcome. Explanation of the clinical relevance of chosen efficacy and harm outcomes is strongly recommended	10-15
Participant timeline	13	Time schedule of enrolment, interventions (including any run-ins and washouts), assessments, and visits for participants. A schematic diagram is highly recommended (see Figure)	Figures 1, 2. Table 1

For peer review only - http://bmjopen.bmj.com/site/about/guidelines.xhtml

Sample size	14	Estimated number of participants needed to achieve study objectives and how it was determined, including clinical and statistical assumptions supporting any sample size calculations	16-18
Recruitment	15	Strategies for achieving adequate participant enrolment to reach target sample size	7-8
**Methods: Assignment of interventions (for controlled trials)**			
Allocation:			
Sequence generation	16a	Method of generating the allocation sequence (eg, computer-generated random numbers), and list of any factors for stratification. To reduce predictability of a random sequence, details of any planned restriction (eg, blocking) should be provided in a separate document that is unavailable to those who enrol participants or assign interventions	10
Allocation concealment mechanism	16b	Mechanism of implementing the allocation sequence (eg, central telephone; sequentially numbered, opaque, sealed envelopes), describing any steps to conceal the sequence until interventions are assigned	10
Implementation	16c	Who will generate the allocation sequence, who will enrol participants, and who will assign participants to interventions	10
Blinding (masking)	17a	Who will be blinded after assignment to interventions (eg, trial participants, care providers, outcome assessors, data analysts), and how	10, 16
	17b	If blinded, circumstances under which unblinding is permissible, and procedure for revealing a participant's allocated intervention during the trial	Can be obtained from authors
**Methods: Data collection, management, and analysis**			
Data collection methods	18a	Plans for assessment and collection of outcome, baseline, and other trial data, including any related processes to promote data quality (eg, duplicate measurements, training of assessors) and a description of study instruments (eg, questionnaires, laboratory tests) along with their reliability and validity, if known. Reference to where data collection forms can be found, if not in the protocol	9-15

For peer review only - http://bmjopen.bmj.com/site/about/guidelines.xhtml

	18b	Plans to promote participant retention and complete follow-up, including list of any outcome data to be collected for participants who discontinue or deviate from intervention protocols	n/a
Data management	19	Plans for data entry, coding, security, and storage, including any related processes to promote data quality (eg, double data entry; range checks for data values). Reference to where details of data management procedures can be found, if not in the protocol	20-21
Statistical methods	20a	Statistical methods for analysing primary and secondary outcomes. Reference to where other details of the statistical analysis plan can be found, if not in the protocol	16-17
	20b	Methods for any additional analyses (eg, subgroup and adjusted analyses)	16-17
	20c	Definition of analysis population relating to protocol non-adherence (eg, as randomised analysis), and any statistical methods to handle missing data (eg, multiple imputation)	Can be obtained from authors
**Methods: Monitoring**			
Data monitoring	21a	Composition of data monitoring committee (DMC); summary of its role and reporting structure; statement of whether it is independent from the sponsor and competing interests; and reference to where further details about its charter can be found, if not in the protocol. Alternatively, an explanation of why a DMC is not needed	20-21
	21b	Description of any interim analyses and stopping guidelines, including who will have access to these interim results and make the final decision to terminate the trial	n/a
Harms	22	Plans for collecting, assessing, reporting, and managing solicited and spontaneously reported adverse events and other unintended effects of trial interventions or trial conduct	20-21
Auditing	23	Frequency and procedures for auditing trial conduct, if any, and whether the process will be independent from investigators and the sponsor	Can be obtained from authors
**Ethics and dissemination**			

For peer review only - http://bmjopen.bmj.com/site/about/guidelines.xhtml

Research ethics approval	24	Plans for seeking research ethics committee/institutional review board (REC/IRB) approval	10
Protocol amendments	25	Plans for communicating important protocol modifications (eg, changes to eligibility criteria, outcomes, analyses) to relevant parties (eg, investigators, REC/IRBs, trial participants, trial registries, journals, regulators)	Can be obtained from authors
Consent or assent	26a	Who will obtain informed consent or assent from potential trial participants or authorised surrogates, and how (see Item 32)	7-8
	26b	Additional consent provisions for collection and use of participant data and biological specimens in ancillary studies, if applicable	n/a
Confidentiality	27	How personal information about potential and enrolled participants will be collected, shared, and maintained in order to protect confidentiality before, during, and after the trial	20-21
Declaration of interests	28	Financial and other competing interests for principal investigators for the overall trial and each study site	22
Access to data	29	Statement of who will have access to the final trial dataset, and disclosure of contractual agreements that limit such access for investigators	Can be obtained from authors
Ancillary and post-trial care	30	Provisions, if any, for ancillary and post-trial care, and for compensation to those who suffer harm from trial participation	n/a
Dissemination policy	31a	Plans for investigators and sponsor to communicate trial results to participants, healthcare professionals, the public, and other relevant groups (eg, via publication, reporting in results databases, or other data sharing arrangements), including any publication restrictions	22
	31b	Authorship eligibility guidelines and any intended use of professional writers	n/a
	31c	Plans, if any, for granting public access to the full protocol, participant-level dataset, and statistical code	n/a
**Appendices**			

For peer review only - http://bmjopen.bmj.com/site/about/guidelines.xhtml

Informed consent materials	32	Model consent form and other related documentation given to participants and authorised surrogates	Can be obtained from authors
Biological specimens	33	Plans for collection, laboratory evaluation, and storage of biological specimens for genetic or molecular analysis in the current trial and for future use in ancillary studies, if applicable	n/a

*It is strongly recommended that this checklist be read in conjunction with the SPIRIT 2013 Explanation & Elaboration for important clarification on the items. Amendments to the protocol should be tracked and dated. The SPIRIT checklist is copyrighted by the SPIRIT Group under the Creative Commons "Attribution-NonCommercial-NoDerivs 3.0 Unported" license.

For peer review only

BMJ Open

Evaluation of a personalised adherence intervention to improve photoprotection in adults with Xeroderma Pigmentosum (XP): Protocol for the trial of XPAND

Journal:	*BMJ Open*
Manuscript ID	bmjopen-2018-028577.R2
Article Type:	Protocol
Date Submitted by the Author:	30-May-2019

Complete List of Authors:	Walburn, Jessica; King's College London, Institute of Pharmaceutical Science<br>Norton, Sam; King's College London, Psychology Department, Insitute of Psychiatry<br>Sarkany, Robert ; Guy's and St Thomas' NHS Foundation Trust, St John's Institute of Dermatology<br>Sainsbury, Kirby; Newcastle University, Institute of Health and Society<br>Araújo-Soares, Vera; Newcastle University, Institute of Health and Society<br>Morgan, Myfanwy; King's College London, Institute of Pharmaceutical Science<br>Canfield, Martha; King's College London, Psychology Department, Insitute of Psychiatry<br>Foster, Lesley; Guy's and St Thomas' NHS Foundation Trust, St John's Institute of Dermatology<br>Heydenreich, Jakob; Bispebjerg Hospital, Department of Dermatology<br>McCrone, Paul<br>Mander, Adrian; University of Cambridge, MRC Biostatistics Unit, Institute of Public Health<br>Sniehotta, Falko<br>Wulf, Hans Christian; Bispebjerg Hospital, Department of Dermatology<br>Weinman, John; Kings College London, Institute of Pharmaceutical Sciences & Institute of Psychiatry

**Primary Subject Heading**:	Dermatology
Secondary Subject Heading:	Evidence based practice
Keywords:	Adherence, RCT, Photoprotection, Behaviour change, Xeroderma Pigmentosum, Photodermatology < DERMATOLOGY

SCHOLARONE™ Manuscripts

Evaluation of a personalised adherence intervention to improve photoprotection in adults with Xeroderma Pigmentosum (XP): Protocol for the trial of XPAND

Authors:

Jessica Walburn *[1], Sam Norton [2], Robert Sarkany [3], Kirby Sainsbury [4],Vera Araújo-Soares [4], Myfanwy Morgan[1], Martha Canfield[2], Lesley Foster [3], Jakob Heydenreich[5], Paul McCrone [6], Adrian Mander [7], Falko F Sniehotta [4], Hans Christian Wulf [5], John Weinman [1]

Affiliations:

1. School of Cancer and Pharmaceutical Sciences, Faculty of Life Sciences and Medicine King's College London, UK
2. Health Psychology Section, Institute of Psychiatry, Psychology & Neuroscience, King's College London, UK; Department of Inflammation Biology, Faculty of Life Sciences and Medicine, King's College London, UK
3. National Xeroderma Pigmentosum Service, Guy's and St. Thomas' NHS Foundation Trust, London, UK
4. Institute of Health & Society, Faculty of Medical Sciences, Newcastle University, UK
5. Department of Dermatology D92, Bispebjerg Hospital, Copenhagen, Denmark.
6. Health Service & Population Research, Institute of Psychiatry, Psychology & Neuroscience, King's College London, UK
7. Cambridge Institute of Public Health, Cambridge University, Cambridge, UK

*Corresponding author: Dr Jessica Walburn, Clinical Practice & Medication Use Group, School of Cancer and Pharmaceutical Sciences, King's College London, 150 Stamford Street, London SE1 9NH, UK; email: jessica.2.walburn@kcl.ac.uk;

For peer review only - http://bmjopen.bmj.com/site/about/guidelines.xhtml

ABSTRACT

Introduction: Poor adherence to photoprotection for people with Xeroderma Pigmentosum (XP) can be life-threatening. A randomised controlled trial (RCT) is being conducted to test the efficacy of a personalised adherence intervention (XPAND) to reduce the level of ultraviolet radiation (UVR) reaching the face, by improving photoprotection activities in adults with XP. Methods and analysis: A two-armed parallel groups RCT where we will randomise 24 patients with sub-optimal adherence to either an intervention group receiving XPAND in 2018 or a delayed intervention group who receive XPAND in 2019. XPAND involves seven sessions, one-to-one with a facilitator, using behaviour change techniques and specially designed materials to target barriers to photoprotection. Following baseline assessment in April 2018 (t0) and intervention, the primary outcome will be measured across 21 consecutive days in June and July 2018 (t1). The primary outcome is the average daily UVR dose to the face (D-to-F), calculated by combining objective UVR exposure at the wrist, (measured by a dosimeter) with face photoprotection activities recorded on a daily diary. Secondary outcomes include average daily UVR D-to-F across 21 days in August (t2); psychosocial process variables measured by daily questions (t0, t1, t2) and self-report questionnaires [t0, t1, t2, December 2018 (t3)]. Intervention cost-utility is assessed by service use and personal cost questionnaires (t0, t3). The delayed intervention control arm participants will complete three further assessments in April 2019 (t4) and June-July 2019 (t5), and December 2019 (t6) with dosimetry and daily UVR protection diary completed for 21 days at t4 and t5. A process evaluation will be conducted using mixed-methods. Ethics and dissemination: Ethical approval has been received from West London & GTAC REC

For peer review only - http://bmjopen.bmj.com/site/about/guidelines.xhtml

17/LO/2110. Results will be disseminated in peer reviewed journals and at conferences. This study tests a novel intervention, which, if successful will be integrated into routine care.

Trial registration number: ClinicalTrials.gov Identifier: NCT03445052

Key words: Adherence; RCT; Photoprotection; Behaviour change; Xeroderma Pigmentosum; Photodermatology

Strengths and limitations of this study

- To the best of our knowledge, this is the first RCT to evaluate an adherence intervention designed to improve photoprotection in people diagnosed with XP.
- We have a primary outcome which is clinically relevant.
- We control for seasonal differences in environmental UVR exposure by comparing between groups across the same weeks.
- We include a process evaluation to understand participant views of XPAND which will be important when integrating the intervention into current clinical care.
- Recruitment and retention of participants may be difficult in this extremely rare disease.

For peer review only

For peer review only - http://bmjopen.bmj.com/site/about/guidelines.xhtml

INTRODUCTION

Xeroderma Pigmentosum (XP) is a very rare genetic condition, where the body is unable to repair ultraviolet radiation (UVR)-induced damage to DNA caused by daylight [1]. Any UVR exposure dramatically increases the chances of developing skin and eye cancers (e.g., rates of non-melanoma skin cancer increase 10,000 fold)[2]. Photoprotection is *recommended* for the healthy population and those taking medications which may cause photosensitivity. However, it is *essential* for people with XP. There is currently no cure for this disease and the only way to minimise and delay the cancers is meticulous and absolute photoprotection every day. Photoprotection involves a complex set of behaviours (i.e., application of broad-spectrum SPF50 sunscreen, wearing long sleeves and long trousers or skirts, wide brimmed hat, glasses, face scarf or buff and avoiding UVR exposure by staying indoors). The best way of protecting the face is by wearing a face visor (a legionnaire style hat with a UVR-protective transparent film at the front). Analysis of self-reported photoprotection used by 21 adults with XP in the UK revealed that over half were using 'very poor' or no photoprotection for at least 20% of all outdoor time [3]. This poor photoprotection has life-threatening consequences.

In common with other rare conditions [4], little is known about the psychological and behavioural patient-related factors that have the potential to improve health outcomes in patients with XP. In recognition of this deficit, the National Institute of Health Research (NIHR) agreed to fund a novel programme of research to investigate the psychosocial factors associated with non-adherence to photoprotection in XP (RP-PG-1212-20009: Developing a psychological intervention to improve ultraviolet protection and clinical outcomes in XP). The aims of the grant were two-fold: to identify the drivers of non-

For peer review only - http://bmjopen.bmj.com/site/about/guidelines.xhtml

adherence using complex mixed-methods research (qualitative interviews; cross-sectional survey; n-of-1; diary-dosimeter measures)[5] (known as "Phase One") and to use this knowledge to design and test an intervention to improve adherence ("Phase Two"). We identified 17 modifiable psychosocial drivers of non-adherence to photoprotection activities from Phase One. These drivers or barriers to optimal photoprotection relate to factors influencing the motivation to protect (e.g., doubts about perceived necessity of photoprotection) and volitional factors limiting the enactment of photoprotection activities, even if motivation is high (e.g., lack of habit). Findings from the mixed-methods research studies are published separately [3 6 7].

We aimed to systematically develop an adherence intervention to improve photoprotection in XP. There is growing support for interventions focused on changing patient beliefs about their illness and treatment, translating into improvements in adherence across a range of chronic conditions [8-10]. This suggests that addressing unhelpful illness and treatment beliefs may be an important treatment target for photoprotection in XP. Furthermore, a recent systematic review found evidence in support of the effectiveness of psychosocial interventions to improve adherence to photoprotective activities among non-XP individuals at elevated risk for melanoma (due to personal or family history [11]).

We designed a personalised intervention titled, '*XPAND: Enhancing XP Photoprotection Activities – New Directions*', using Intervention Mapping [12]. Intervention Mapping starts by breaking the desired behaviour and determinants of behaviour into constituent parts, which are mapped to theory and translated to intervention components. Relevant theories to guide the intervention design included the Necessity and Concerns Framework [13], Common Sense Model of Illness Regulation [14], and the Theoretical Domains Framework (not a theory itself but it combines the components of social cognition models

For peer review only - http://bmjopen.bmj.com/site/about/guidelines.xhtml

used to explain behaviour)[15]. A full description of the development of the intervention, the process of personalisation, and the final intervention product will be reported separately.

This paper describes the protocol for a randomised controlled trial (RCT) to test the efficacy of XPAND to lower the dose of UVR reaching the face, by improving adherence to photoprotection. This can be achieved by minimising overall UVR exposure (i.e., time spent outside) and by increasing level of photoprotection worn or applied when outside. The primary objective is to reduce the average daily UVR dose to the face (D-to-F) across 21 days in June to July (2018), immediately after the delivery of the main intervention. Secondary objectives are: to maintain reductions in average daily UVR D-to-F across 21 days in August 2018 (after a booster session has been delivered to the intervention group); to increase and then maintain daily ratings of mood, self-efficacy, goal priority, automaticity and photoprotection activities across the two 21-day periods. Tertiary objectives are to explore intervention-related changes from baseline. A qualitative process evaluation will investigate the acceptability, feasibility, and change mechanisms from the perspective of the participant. We will conduct a cost-utility analysis, which will indicate whether implementation into clinical care is economically viable.

METHODS AND ANALYSIS

Participants and recruitment

People diagnosed with XP and registered at the National XP Service at Guy's and St Thomas' NHS Foundation Trust will be recruited into the trial. Eligible patients will be sent an invitation letter, followed by a telephone call from the research nurse, and those interested will be sent the study information leaflet. Following a second call, if they wish to participate, they will give written consent at a home visit. Patients will be reminded that

For peer review only - http://bmjopen.bmj.com/site/about/guidelines.xhtml

participation in the study is voluntary and that they may withdraw at any time. No payment will be given for participation. Screening and recruitment will take place between February-March 2018.

Inclusion criteria

1. A confirmed diagnosis of Xeroderma Pigmentosum (reduced DNA repair activity in DNA fibroblast assay, in a clinical context compatible with a diagnosis of XP, and with confirmation by finding pathogenic mutations in an XP gene on gene sequencing)
2. Aged ≥ 16 years
3. Sub-optimal adherence to photoprotection when outdoors, as identified by the XP clinical team from data held in medical notes, or by the research team from data collected during the Phase One studies:
    a. Score <20 on our Adherence to Photoprotection scale (where a total score of 25 indicates optimal photoprotection) [16]
    b. Using photoprotective clothing combinations for the face that have been assessed by the clinical team as anything other than 'excellent' or 'very good' anytime outdoors, recorded on the daily UVR protection diary [3]
    c. Having a 'resistant' or 'integrated' mode of adjustment to photoprotection, as identified in the qualitative analysis [7]

Exclusion criteria

1. Diagnosed with cognitive impairment (XP or non-XP related) due to potential impact on the efficacy of the intervention and on the participants' experiences of taking part.

For peer review only - http://bmjopen.bmj.com/site/about/guidelines.xhtml

2. Non-fluent in English (to enable in-depth discussion with the intervention facilitators)
3. Diagnosed with current clinical depression or anxiety, as detailed in medical notes, or confirmed after completion of Hospital Anxiety and Depression Scale (HADS)[17] at the baseline home visit

Study design and flowchart

The XPAND trial is a phase-II two-armed parallel group RCT, with a delayed intervention control arm. Participants are randomised to either the intervention group who receive the XPAND intervention in addition to routine care between April and June 2018 or to the control group who receive routine care in 2018 and then XPAND between April and June 2019. The delayed intervention control group is included to maximise information collection about the intervention due to the extremely rare nature of XP. For example, achievable sample sizes limit the ability to test between-group differences for the psychosocial outcomes with acceptable power and the delayed intervention group allows for intervention-related changes from baseline testing, which is less robust but still useful.

Patient flow through the study is described in Figure 1. The baseline assessment takes place in April 2018 (t0). Post-randomisation assessments take place after completion of the intervention in June to July 2018 (t1), after a booster session in August 2018 (t2), and a long-term follow-up in December 2018 (t3). All participants wear a wrist-worn UVR dosimeter continuously from t0-t2 and complete daily UVR protection diaries for 21 consecutive days at each assessment time point. At each assessment, participants additionally complete patient reported outcomes. The t3 follow-up does not involve dosimetry or daily UVR protection diary. In addition, the delayed intervention control arm only complete three

For peer review only - http://bmjopen.bmj.com/site/about/guidelines.xhtml

further assessments in April 2019 (t4) and June-July 2019 (t5), and December 2019 (t6) with dosimetry and daily UVR protection diary completed for 21 days at t4 and t5.

A nested qualitative study examining fidelity, acceptability, and mechanisms of change will be conducted in the intervention group after the August assessment period is completed.

[please insert Figure 1 here]

The study is funded by the NIHR Programme Grants for Applied Research scheme (RP-PG-1212-20009) and received ethical approval from the West London & GTAC Research Ethics Committee 17/LO/2110.

Randomisation

Participants will be randomised, in blocks using an equal allocation ratio, to receive XPAND immediately (2018) or to the delayed group stratified by burning type to attempt to balance those with an extreme (i.e., scoring between 1-3 on the sunburn severity score [18]) versus normal burning response. Participants who are in the same family will be randomised as a cluster to the same group to avoid contamination. Since all participants will be recruited at the point of randomisation, the trial statistician (SN) will generate a random allocation sequence for all participants together, using a computer programme with fixed block sizes of 4, to ensure equal allocation to both groups. To protect the integrity of the randomisation, participants in the immediate intervention group will be asked not to reveal their allocation to those outside their immediate family. Group allocation will be concealed from the XP clinical team who are not part of the research team (excluding the PI) to avoid inadvertent changes to the standard care of these participants during the trial (e.g.,

For peer review only - http://bmjopen.bmj.com/site/about/guidelines.xhtml

greater/lesser discussion of adherence during routine clinical appointments). Moreover, as both the control and intervention groups might receive opportunistic encouragement from the clinical team to protect, this would not adversely affect the trial outcome.

Primary outcome

The primary outcome is the average daily UVR D-to-F (standard erythemal dose; SED), across 21 consecutive days between June and July 2018 (t1), following completion of six out of seven intervention sessions by the intervention group. UVR D-to-F is estimated by combining data from a UVR dosimeter worn on the wrist (SunSaver 3, Bispebjerg Hospital, Copenhagen, Denmark) [19] and the patient's photoprotective activities, self-reported using the daily UVR protection diary. The dosimeter was set to measure UVR levels every five seconds and record the average every 5 minutes, which has been validated as providing reliable measurements of total UVR exposure over the course of a day [20]. The combination of the daily UVR protection diary and dosimeter to estimate UVR D-to-F has been used in our earlier research with this population (Phase One) and demonstrated to function sufficiently well to be the primary outcome in this trial. The paper-based daily UVR protection diary allows participants to record periods of time spent outside (in 15-minute intervals) and the timing of any specific photoprotection activities while outside (i.e., wearing a face visor, hat, hoodie worn-up, glasses, scarf or face buff, or applying sunscreen and lip-block) by drawing a line on a grid (see Supplementary file 1.). To avoid errors in recording each participant will receive training on how to complete the diary. UVR D-to-F will be calculated using the dose recorded at the wrist weighted by the level of protection provided by the combination facial photoprotection behaviours used [5].

For peer review only - http://bmjopen.bmj.com/site/about/guidelines.xhtml

Secondary outcomes

The following outcomes relate to between-group comparisons in 2018 and are recorded on the daily UVR protection diary. UVR-related outcomes also use data from the dosimeter.

a. Average daily UVR D-to-F across 21 consecutive days in August 2018 (t2). This is to investigate the stability of any improvement identified in the primary outcome period (June-to-July 2018), as it will be 4-6 weeks after the main intervention and follows session 7 (the booster session).
b. Average daily total UVR exposure during each of the 21-day periods (t1, t2) and the average daily time outside across all daylight hours and restricted to 11-3pm, when UVR levels are at their highest. This data will be gathered from the dosimeter and the UVR protection diary.
c. Average daily proportion of time outside across daylight hours and between 11-3pm with "very good" or "excellent" face photoprotection during each of the 21-day periods (t1, t2). The categorisations relate to the Daily Photoprotection Scale (DPS) [3] developed for the Phase One n-of-1 study in consultation with the UK XP clinical team. Facial photoprotection activities (excluding sunscreen) are ranked according to their relative level of photoprotection. The following combinations are classed as "very good photoprotection": hat, glasses and scarf/buff; hat, glasses, scarf/buff and hoodie. "Excellent photoprotection" is wearing a face visor, which completely covers the face.
d. Average daily proportion of time outside across daylight hours with sunscreen protection, defined as when sunscreen was applied within the previous 3 hours, during each of the 21-day periods (t1, t2).

For peer review only - http://bmjopen.bmj.com/site/about/guidelines.xhtml

e. Average daily number of times sunscreen is applied irrespective of time outside during each of the 21-day periods (t1, t2).

f. Average daily measures of mood, automaticity of photoprotection activities, prioritisation of photoprotection compared to other priorities, and level of self-efficacy to manage barriers during the two follow-up periods (t1, t2) will be assessed. The single items have been adapted from ecological momentary assessment questions used in our n-of-1 study [3] *(e.g., How much do you agree that UVR protection of your face today was something you did automatically without thinking* 0=strongly disagree – 10=strongly agree) and are included on the UVR protection diary (see Supplementary file 1.).

Tertiary outcomes

The following outcomes are assessed once, at the start of each 21-day period (t0, t1, t2, t3). Health-related quality of life (HRQoL), psychological well-being, automaticity of photoprotection activities, self-efficacy in the context of barriers, and self-rated photoprotection adherence will be measured using self-report questionnaires. Data will be combined across t1 and t2 to assess proximal impact of the intervention and t3 will be used to assess stability of impact.

See Table 1 for the schedule of self-reported measures.

a. HRQoL will be assessed using the EQ-5D-5L[21]

b. Emotional well-being will be measured by the Short-form Warwick Edinburgh Mental Well-Being scale (SWEMWBS)[22]

c. Automaticity of photoprotection activities will be assessed using the 4-item Self-Report Behavioural Automaticity Index (SRBAI)[23], adapted to photoprotection. It is a validated

For peer review only - http://bmjopen.bmj.com/site/about/guidelines.xhtml

subscale of the Self-Report Habit Index [24] which focuses on the automaticity of behaviour. It asks respondents to rate the extent to which they agree with each statement on a seven-point Likert-type scale [e.g., *UVR photoprotection is something I do without thinking* (1= strongly disagree-7= strongly agree)]. The average of responses gives a score between 1 and 7, with higher scores indicating greater automaticity.

d. Self-efficacy to photoprotect will be measured using a 21-item scale [Photoprotection Self-Efficacy Questionnaire (PhotoSEQ)] developed for this study, as no validated questionnaire specific to photoprotection activities in the presence of barriers, as recommended by Bandura (2006) [25], was identified (see Supplementary file 2.). Three items ask the respondent to rate their level of confidence that they can carry out a type of photoprotection activity (e.g., shifting timing and/or duration of outdoor activity, photoprotection using clothing, correctly apply sunscreen) on a ten-point scale (0=Not at all – 10=very confident); two items ask separately about confidence to wear photoprotective clothing and apply sunscreen in the presence of nine different barriers in the following 4 weeks *[e.g., How confident are you that you can photoprotect even if/when: unexpected things get in the way*]. Two subscales are calculated, an average self-efficacy score for sunscreen and similarly for photoprotective clothing and shifting time/duration of activity. Higher scores indicate greater self-efficacy.

e. A secondary brief measure of photoprotection activities was developed to assess photoprotection to indicate whether improvements in photoprotection are maintained, when participants will not be completing the daily UVR protection diary at t3 (see Supplementary file 3.). To allow comparison with the diary, it will be completed at all follow-up points. The Brief Photoprotection Adherence Questionnaire (BPAQ) has 5 items and assesses duration of time outdoors, and photoprotection used when outdoors

For peer review only - http://bmjopen.bmj.com/site/about/guidelines.xhtml

during the previous 7 days. Three items assess different ways of protecting outdoors: how often respondents wore protective clothing (e.g., *When you went outside, how often did you protect your face against UVR using protective clothing?* 0=never – 10=all the time); the number of days sunscreen was applied in the morning (0-7 days); and how often sunscreen was re-applied when outdoors for longer periods (0=not at all – 10=always). Two items ask respondents to estimate average duration of time outdoors each day (Never, <30mins, 31mins-1 hour, 2 hours, upwards in hourly intervals to >8 hours) and average duration of time outdoors each day between 11am-3pm when environmental UVR levels are highest. Each item will be analysed separately.

Further exploratory analyses will examine change from baseline incorporating the delayed intervention group allowing for higher power to assess intervention-related changes. These will be undertaken unblinded to group allocation after main efficacy analyses are complete.

Table 1. Summary of self-reported measures schedule

	**Intervention and control groups 2018**				**Delayed intervention group only 2019**		
	**Baseline (t0) (April)**	**Follow-up (t1) (June-July)**	**Follow-up(t2) (August)**	**Follow up (t3) (December)**	**Follow-up (t4) (April)**	**Follow-up (t5) (June-July)**	**Follow-up (t6) (December)**
UVR Protection Diary	✓	✓	✓		✓	✓	
EuroQol five dimensions questionnaire (EQ-5D)	✓	✓	✓	✓	✓	✓	✓
Short-form Warwick-Edinburgh Mental Well-Being Scale (SWEMWBS)	✓	✓	✓	✓	✓	✓	✓
Self-Report Behavioural	✓	✓	✓	✓	✓	✓	✓

For peer review only - http://bmjopen.bmj.com/site/about/guidelines.xhtml

Automaticity Index' (SRBAI)							
Photoprotection self-efficacy Questionnaire (PhotoSEQ)	✓	✓	✓	✓	✓	✓	✓
Brief Photoprotection Adherence questionnaire (BPAQ)	✓	✓	✓	✓	✓	✓	✓
Service Use Questionnaire (SUQ)	✓			✓	✓		✓
Feedback questionnaire (intervention grp only)		✓				✓	

The XPAND intervention

XPAND is composed of a combination of one-to-one sessions and materials purpose-designed to target barriers to photoprotection. It was designed to be delivered by a healthcare professional (HCP) without specialist psychological training (e.g., clinical nurse specialist). In the trial context, facilitators will be two psychologists and a research nurse.

One-to-one sessions

Facilitators will deliver 7x one-to-one sessions to each participant. Further information on the structure of the intervention is available in Figure 2. Each session will include a combination of personalised and generic content. The intervention content and techniques were systematically developed and selected using Intervention Mapping [12]. Core strategies delivered to all participants will include those to increase self-efficacy, self-regulatory skills, and the automaticity of photoprotection. In addition, personalised modules will be delivered, as needed, selected initially based on data collected in Phase One studies and a profiling questionnaire, and iteratively as additional barriers emerge during the sessions. The facilitators will use a communication style consistent with Motivational Interviewing [26]. They will be guided by a manual, including theory-based behaviour change techniques

For peer review only

For peer review only - http://bmjopen.bmj.com/site/about/guidelines.xhtml

(BCTs) mapped to a recognised taxonomy [27] and specific techniques from other therapeutic approaches (e.g., Acceptance and Commitment Therapy) [28].

Intervention materials

Patient-facing materials have been developed and will be provided to participants in session 1 and used throughout the intervention to facilitate discussion, provide real-life examples, and reinforce the concepts and skills discussed during the sessions. These are summarised in Figure 2.

[Please insert Figure 2 here]

Sample size calculations

The target sample size for the study is a total of 24 patients (i.e. 12 per group), which was based on the ability to detect an average daily UVR D-to-F difference of 0.10 SED between the groups during the June to July 2018 assessment period. This difference was considered clinically meaningful and potentially achievable due to the personalised nature of the intervention. From our Phase One study of UVR exposure, the average daily UVR D-to-F for people diagnosed with XP was 0.27 SED (SD=0.14), with a within-person correlation of r_{ICC}=0.31. Thus the anticipated difference relates to an effect size of d=0.73, or just over a one-third reduction in average daily UVR D-to-F. Adjusting for the design effect due to the dependence of assessments within individuals, a sample size of 20 patients with 21 daily observations has 80% power to detect a reduction of 0.10 SED in average daily UVR D-to-F at the 5% significance level. Accounting for attrition of 20%, based on our previous research, the target sample size was set at 24.

Statistical analyses

For peer review only - http://bmjopen.bmj.com/site/about/guidelines.xhtml

The main efficacy analyses will be conducted by the trial statistician (SN) following a pre-specified analysis plan and blind to group allocation. The analysis will follow the intention to treat principle with individuals analysed within the groups to which they were randomised irrespective of whether they received or persisted with the intervention.

The treatment effect on the primary outcome of daily UVR D-to-F over 21 days between June and July 2018 and secondary outcome of daily UVR D-to-F over 21 days in August will be estimated simultaneously using a linear mixed model. Given the skewed distribution of UVR D-to-F, a logarithmic transformation will be applied and robust standard errors will be estimated. A random intercept will account for the repeated assessments of UVR D-to-F within individuals across all 42 days, with an autoregressive error structure to account for correlation between assessments across successive days. In addition to the dummy coded group variable, other covariates in the model include a dummy coded variable for the assessment period (June-July vs August), an assessment period by group interaction, the patients baseline average daily UVR, an indictor variable for the patients propensity to burn, and the background level of UVR recorded by the observatory closest to the patients' house on the day. Data will be summarised as the empirical Bayes estimate of the average daily UVR D-to-F in SEDs for each assessment period for each group.

This approach allows for missing daily data within each individual under the assumption that data are missing at random. We will run sensitivity analyses to explore the influence of missing data on the effect estimates, so as to allow for a consideration of the likely plausible range for the treatment effect under a number of missing not at random assumptions. For average daily assessments, where these are not available for the full 21 days, this will involve imputing missing values under a range of conservative assumptions using the

For peer review only - http://bmjopen.bmj.com/site/about/guidelines.xhtml

average daily values of the group to which the individual is assigned using a pattern mixture model approach (e.g., average daily level + 0.0SED to 0.5SED).

Treatment effects for average daily levels of mood, self-efficacy, goal priority, automaticity and time outside will be analysed for both assessment periods simultaneously using mixed-effects as described above. Treatment effects for adherence, self-efficacy, automaticity, HRQoL, and psychological well-being taken at the start of the June and August assessment periods will also be estimated using linear mixed models but will specify an unconstrained error structure. It is not anticipated that transformation will be required to account for skew in any of these outcomes; however, robust standard errors will be estimated. Covariates included in the analysis will be the same as above except that baseline levels of the outcomes will be included in place of baseline average daily UVR D-to-F. Scale scores will be calculated using proration to account for missing items within the scale where there is at most one-third of items missing. For example, where scale includes 6 items, at least 4 items must have been completed otherwise the scale score will be set to missing for that individual.

Further exploratory analysis using dynamic regression models will evaluate changes in both level and variability in daily UVR exposure over time between March and September, accounting for treatment using a regression discontinuity approach. This is possible as the dosimeter will be worn for the entire period. However, due to patient burden and since the UVR protection diary is only completed for three-week intervals, it will also be necessary to calculate UVR D-to-F using average daily photoprotection during the pre and post diary intervals. This differs from the main analysis where UVR D-to-F is calculated directly based on photoprotection activities recorded on the diary.

For peer review only - http://bmjopen.bmj.com/site/about/guidelines.xhtml

Trial of XPAND: Protocol 28.05.19 Version 9

Process evaluation

A mixed-methods process evaluation, using qualitative interviews and self-report measures, will explore the acceptability of the intervention, changes in photoprotection activities, and psychosocial mechanisms of change. A brief intervention feedback questionnaire, completed immediately after session 6, will be used to record more proximal perceptions of the main content of intervention. Responses to this questionnaire and the profiling questionnaire (see Figure 2) will be used to guide prompts during these interviews and give insight into psychosocial mechanisms of change.

The feedback questionnaire is adapted from one used to assess a fat reduction intervention [29]. It has 5-items assessing overall perceptions of the programme and its components, the impact on photoprotection activities, and whether psychosocial variables have changed as a result of the intervention. The questions assess the extent to which respondents agree with statements about the intervention (e.g., *"Overall the programme was interesting*" 1=strongly disagree – 5=completely agree) (see Supplementary file 4.).

In-depth qualitative interviews will be conducted by a research nurse and health psychologist who were involved in the design and delivery of the intervention. They will not interview participants to whom they delivered the intervention. Interviews will be based on a topic guide and will explore participants' views of and experiences with XPAND, including more practical delivery-related aspects, such as the optimal number of sessions, preference for telephone or skype sessions, and the value of any further booster sessions. All interviews will be audio-recorded, transcribed, and coded using thematic analysis [30 31] in NVivo10.

Fidelity Assurance

For peer review only - http://bmjopen.bmj.com/site/about/guidelines.xhtml

A fidelity assessment will be undertaken to examine the extent to which facilitators delivered the key components of the intervention, specified in the intervention manual, during the face-to-face sessions. Independent researchers will apply a fidelity checklist to audio recordings of all session 1s and session 6s, as well as and a random selection of subsequent sessions.

Evaluation of the cost-utility of XPAND

A decision model will be used to assess the cost-effectiveness of the intervention. Decision models allow outcomes and costs associated with alternative care process to be investigated via simulations. Model structures simplify the care process such that specific aspects can be focussed on. Advantages of these models are that they can be adapted to reflect the outcomes and costs that occur in a variety of settings (and therefore aid generalisability); they allow evidence to be generated in a time and cost-efficient way; and they enable interventions to be evaluated that may be precluded using trial methods. The structure of the model to be used here will be developed by the health economists in collaboration with the clinical researchers, and will consist of health states that patients may be in over time. Transition between the states will be informed by literature on the progression of the condition, expert opinion, and data collected through the trial. The impact of the intervention on these transitions will be determined through the clinical trial. A health service perspective will be used followed by a societal perspective, which will incorporate broader costs.

Service use will be measured via completion of a Client Service Receipt Inventory (CSRI) [32]. This retrospectively assesses use of primary and secondary healthcare services (including surgical interventions), social care, tests/investigations, and aids and adaptations

For peer review only - http://bmjopen.bmj.com/site/about/guidelines.xhtml

(see Supplementary file 5.). The cost of these inputs will be calculated by combining the service use data with appropriate unit cost information. Other impacts of XP include additional financial costs for the patient (e.g., sunscreen); and time lost from work/education by patients (e.g., to attend appointments, receive treatment, manage symptoms). Costs of these effects will be calculated using average wage rates and information on returns to education. Costs will be combined with quality-adjusted life years (QALYs) derived from the EQ-5D-5L. The estimated cost of the intervention will be based on staff time required to deliver it, plus additional training and materials.

Trial management

An independent Trial Steering Committee (TSC) attended by the research team, two independent researchers, and one PPI panel member will meet every 3 months to provide oversight of the trial. Trial data is collected after each measurement period by a member of the research team not delivering the intervention to that individual. Data are managed and entered by the research team, not the statisticians analysing the data. Adverse events are defined as events that occur during participation in the trial. All will be recorded [death; life threatening; hospitalisation; self-harm; attendance at A&E; distress (intervention group)] and reported to the TSC, who will make a judgement on the link to the trial and recommend modifications or discontinuing trial as appropriate.

Patient and Public Involvement

Patient and Public Involvement (PPI) has been integral to every step in the development of this protocol. The PPI panel advised on the study design, particularly related to participant burden of completion of the daily UVR protection diary, which informed the decision to limit the follow-up of the delayed intervention control group to a single period of

For peer review only - http://bmjopen.bmj.com/site/about/guidelines.xhtml

21 days (June-July 2019, t5). In addition, the panel reviewed all the XPAND intervention participant-facing materials which helped ensure they were appropriate and acceptable. The PPI panel were not involved in the recruitment process. A summary of research findings will be sent to all participants and published papers will be made available.

DISCUSSION

To the best of our knowledge this is the first RCT to test a behaviour change intervention to improve adherence to photoprotection in adults with XP; an ambitious undertaking in a rare disease. It uses a robust methodology, both in the systematic development of the intervention and in the use of a clinically-relevant novel measurement approach to estimate dose of UVR reaching the face. The inclusion of a process evaluation will provide insight into the workings of the intervention and the mechanism underlying our trial outcomes, as well as information about the acceptability of XPAND, a key consideration for the implementation of the intervention into routine clinical care. We will also have estimates of the cost-effectiveness of the intervention. Furthermore, over and above the obvious clinical advantages of decreasing the risk of morbidity and mortality associated with skin cancer for the UK XP population, if the intervention is efficacious it will be generalizable to international populations of adults with XP, significant for other conditions requiring photoprotection (e.g., Systemic Lupus Erythematosus) and relevant for healthy populations.

ETHICS AND DISSEMINATION

For peer review only - http://bmjopen.bmj.com/site/about/guidelines.xhtml

This research has been approved by London – West London & GTAC Research Ethics Committee 17/LO/2110. The findings will be published in peer reviewed journals and presented at national and international scientific conferences.

Trial registration number: ClinicalTrials.gov Identifier: NCT03445052

Trial status

Recruitment completed. Patient involvement in the study will conclude in December 2019.

Competing interests

None declared

Acknowledgements

We would like to acknowledge the valuable role of the XP national clinical team (Hiva Fassihi, Tanya Henshaw, Alan Lehmann, Sally Turner) and the PPI panel (Cathy Coleman, Ben Fowler, Ros Tobin, Sandra Webb) in assisting with development of this protocol. In addition, we would like to acknowledge the support of the NIHR team (Hothan Esmael, Jonathan Gabriel, Katherine Horner, Thomas Hutchinson) and Robert Pleass (Dermatology Research Manager Guy's and St. Thomas' NHS Foundation Trust). Many thanks to Federica Picariello for commenting on the final draft.

Contributors

JWa drafted the manuscript with assistance from KS and LF. VAS, MC, JH, PM, AM, MM, SN, KS, RS, FFS, JW, HCW reviewed and commented on the manuscript. All authors were involved in study design. LF, JW and KS are involved in intervention delivery and data collection.

Funding

For peer review only - http://bmjopen.bmj.com/site/about/guidelines.xhtml

For peer review only

This study/project is funded by the National Institute for Health Research (NIHR) [Programme Grant for Applied Research Scheme (RP-PG-1212 20009)]. The views expressed are those of the author(s) and not necessarily those of the NIHR or the Department of Health and Social Care. The trial sponsor is Guy's and St. Thomas' NHS Foundation Trust.

Figure legends

Figure 1. Progression of participants through the study

Figure 2. Structure of the XPAND intervention

References

1. Fassihi H, Sethi M, Fawcett H, et al. Deep phenotyping of 89 xeroderma pigmentosum patients reveals unexpected heterogeneity dependent on the precise molecular defect. *Proceedings of the National Academy of Sciences* 2016;113(9):E1236-E45.
2. Bradford PT, Goldstein AM, Tamura D, et al. Cancer and neurologic degeneration in xeroderma pigmentosum: long term follow-up characterises the role of DNA repair. *J Med Genet* 2011;48(3):168-76. doi: 10.1136/jmg.2010.083022
3. Sainsbury K, Vieira R, Walburn J, et al. Understanding and predicting a complex behaviour using n-of-1 methods: Photoprotection in xeroderma pigmentosum. *Health Psychol* 2018; *37*(12): 1145-1158. doi.org/10.1037/hea0000673
4. Cooper V, Clatworthy J. Adherence research summarised: Unique patient challenges and support solutions in rare diseases. 2016 [Retrieved from: http://www.altantishealthcare.com/].
5. Walburn J, Sarkany R, Norton S, et al. An investigation of the predictors of photoprotection and UVR dose to the face in patients with XP: a protocol using observational mixed methods. *BMJ open* 2017;7(8):e018364; DOI: 10.1136/bmjopen-2017-018364
6. Anderson R, Walburn J, Morgan M. Experiences of stigma over the lifetime of people with xeroderma pigmentosum: A qualitative interview study in the United Kingdom. *J Health Psychol* 2017:1359105317714643.
7. Morgan M, Anderson R, Walburn J, et al. The influence of perceived medical risks and psychosocial concerns on photoprotection behaviours among adults with Xeroderma Pigmentosum: A qualitative interview study in the UK. *BMJ Open* 2019; 9 (2) e024445; DOI: 10.1136/bmjopen-2018-024445
8. Broadbent E, Ellis C, Thomas J, et al. Further development of an illness perception intervention for myocardial infarction patients: a randomized controlled trial. *J Psychosom Res* 2009;67(1):17-23. doi.org/10.1016/j.jpsychores.2008.12.001
9. O'Carroll RE, Chambers JA, Dennis M, et al. Improving adherence to medication in stroke survivors: a pilot randomised controlled trial. *Ann Behav Med* 2013;46(3):358-68. doi.org/10.1007/s12160-013-9515-5

For peer review only - http://bmjopen.bmj.com/site/about/guidelines.xhtml

10. Petrie KJ, Perry K, Broadbent E, et al. A text message programme designed to modify patients' illness and treatment beliefs improves self-reported adherence to asthma preventer medication. *Br J Health Psychol* 2012;17(1):74-84. doi.org/10.1111/j.2044-8287.2011.02033.x
11. Wu YP, Aspinwall LG, Conn BM, et al. A systematic review of interventions to improve adherence to melanoma preventive behaviors for individuals at elevated risk. *Prev Med* 2016;88:153-67. doi.org/10.1016/j.ypmed.2016.04.010
12. Eldredge LKB, Markham CM, Kok G, et al. Planning health promotion programs: an intervention mapping approach: John Wiley & Sons 2016.
13. Horne R, Chapman SC, Parham R, et al. Understanding patients' adherence-related beliefs about medicines prescribed for long-term conditions: a meta-analytic review of the Necessity-Concerns Framework. *PLoS One* 2013;8(12):e80633. doi.org/10.1371/journal.pone.0080633
14. Leventhal H, Meyer D, Nerenz D. The common sense representation of illness danger. In: Rachman S, ed. Contributions to medical psychology. New York: Pergamon Press 1980:7-30.
15. Francis JJ, O'Connor D, Curran J. Theories of behaviour change synthesised into a set of theoretical groupings: introducing a thematic series on the theoretical domains framework. *Implementation Science* 2012;7(1):35. doi.org/10.1186/1748-5908-7-35
16. Canfield M, Norton S, Walburn J, et al. Adherence to face photoprotection behaviours in xererderma pigmentosum patients: initial validation of a new measure. *PsyArXiv* 2018, November 2018. doi.org/10.31234/osf.io/6g7ba
17. Zigmond AS, Snaith RP. The hospital anxiety and depression scale. *Acta Psychiatr Scand* 1983;67(6):361-70. doi/pdf/10.1111/j.1600-0447.1983.tb09716.x
18. Sethi M, Lehmann A, Fawcett H, et al. Patients with xeroderma pigmentosum complementation groups C, E and V do not have abnormal sunburn reactions. *Br J Dermatol* 2013;169(6):1279-87. doi.org/10.1111/bjd.12523
19. Thieden E, Ågren M, Wulf H. The wrist is a reliable body site for personal dosimetry of ultraviolet radiation. *Photodermatol Photoimmunol Photomed* 2000;16(2):57-61. doi.org/10.1034/j.1600-0781.2000.d01-4.x
20. Thieden E, Ågren M, Wulf H. Solar UVR exposures of indoor workers in a Working and a Holiday Period assessed by personal dosimeters and sun exposure diaries. *Photodermatol Photoimmunol Photomed* 2001;17(6):249-55. doi.org/10.1111/j.1600-0781.2001.170601.x
21. Herdman M, Gudex C, Lloyd A, et al. Development and preliminary testing of the new five-level version of EQ-5D (EQ-5D-5L). *Qual Life Res* 2011;20(10):1727-36. doi.org/10.1007/s11136-011-9903-x
22. Stewart-Brown S, Tennant A, Tennant R, et al. Internal construct validity of the Warwick-Edinburgh mental well-being scale (WEMWBS): a Rasch analysis using data from the Scottish health education population survey. *Health and quality of life outcomes* 2009;7(1):15. doi.org/10.1186/1477-7525-7-15
23. Gardner B, Abraham C, Lally P, et al. Towards parsimony in habit measurement: Testing the convergent and predictive validity of an automaticity subscale of the Self-Report Habit Index. *International Journal of Behavioral Nutrition and Physical Activity* 2012;9(1):102. doi.org/10.1186/1479-5868-9-102
24. Verplanken B, Orbell S. Reflections on Past Behavior: A Self-Report Index of Habit Strength. *J Appl Soc Psychol* 2003;33(6):1313-30. doi.org/10.1111/j.1559-1816.2003.tb01951.x

For peer review only - http://bmjopen.bmj.com/site/about/guidelines.xhtml

25. Bandura A. Guide for constructing self-efficacy scales. *Self-efficacy beliefs of adolescents* 2006;5(307-337)
26. Miller WR, Rollnick S. Motivational interviewing: Helping people change: Guilford press 2012.
27. Michie S, Richardson M, Johnston M, et al. The behavior change technique taxonomy (v1) of 93 hierarchically clustered techniques: building an international consensus for the reporting of behavior change interventions. *Ann Behav Med* 2013;46(1):81-95. doi.org/10.1007/s12160-013-9486-6
28. Hayes SC, Strosahl KD, Wilson KG. Acceptance and commitment therapy: Guilford Press New York 1999.
29. Vandelanotte C, De Bourdeaudhuij I, Brug J. Acceptability and feasibility of an interactive computer-tailored fat intake intervention in Belgium. *Health Promot Int* 2004;19(4):463-70. doi.org/10.1093/heapro/dah408
30. Braun V, Clarke V. Using thematic analysis in psychology. *Qualitative Research in Psychology* 2006;3(2):77-101. doi.org/10.1191/1478088706qp063oa
31. Braun V, Clarke V, Terry G. Thematic analysis. In: Rohleder P, Lyons AC, eds. Qual Res Clin Health Psychol. Basingstoke: Palgrave MacMillan 2014:95-114.
32. Beecham J, Knapp M. Costing psychiatric interventions: Gaskell London 2001.

For peer review only

For peer review only - http://bmjopen.bmj.com/site/about/guidelines.xhtml

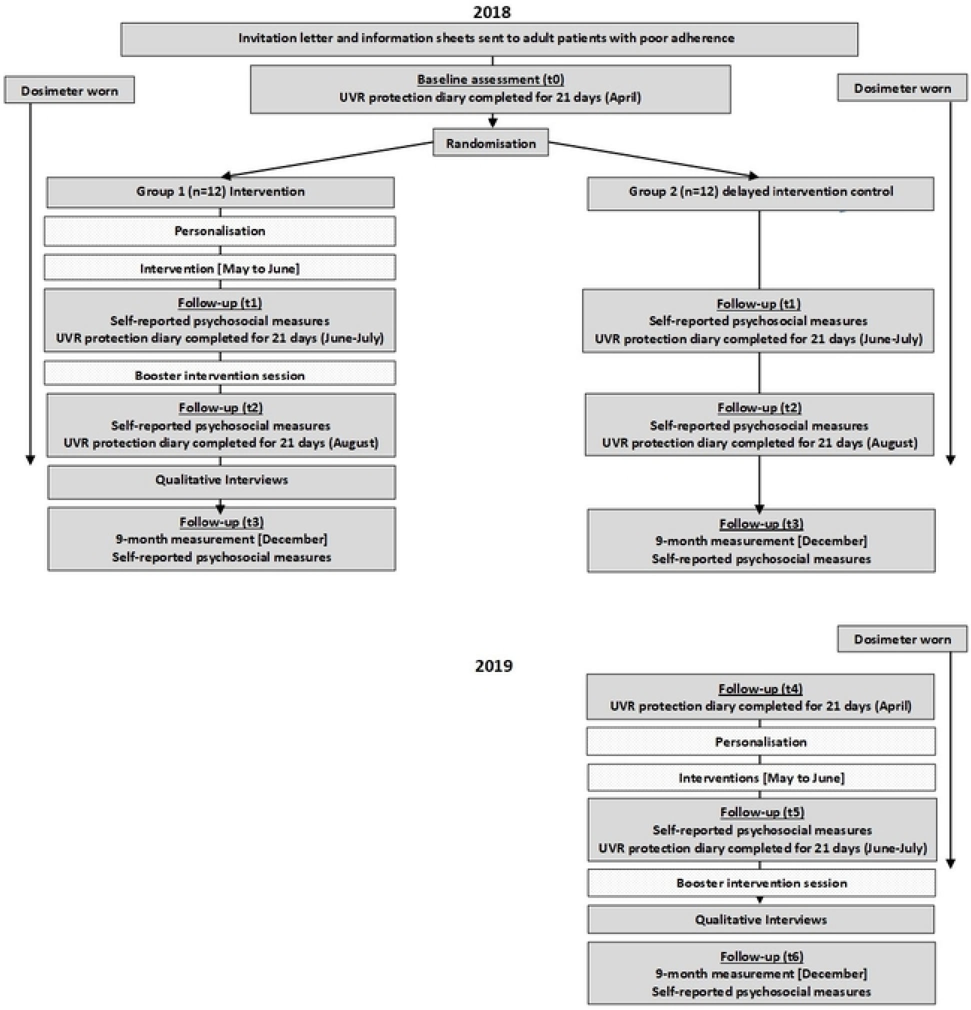

Figure 1. Progression of participants through the study

56x57mm (300 x 300 DPI)

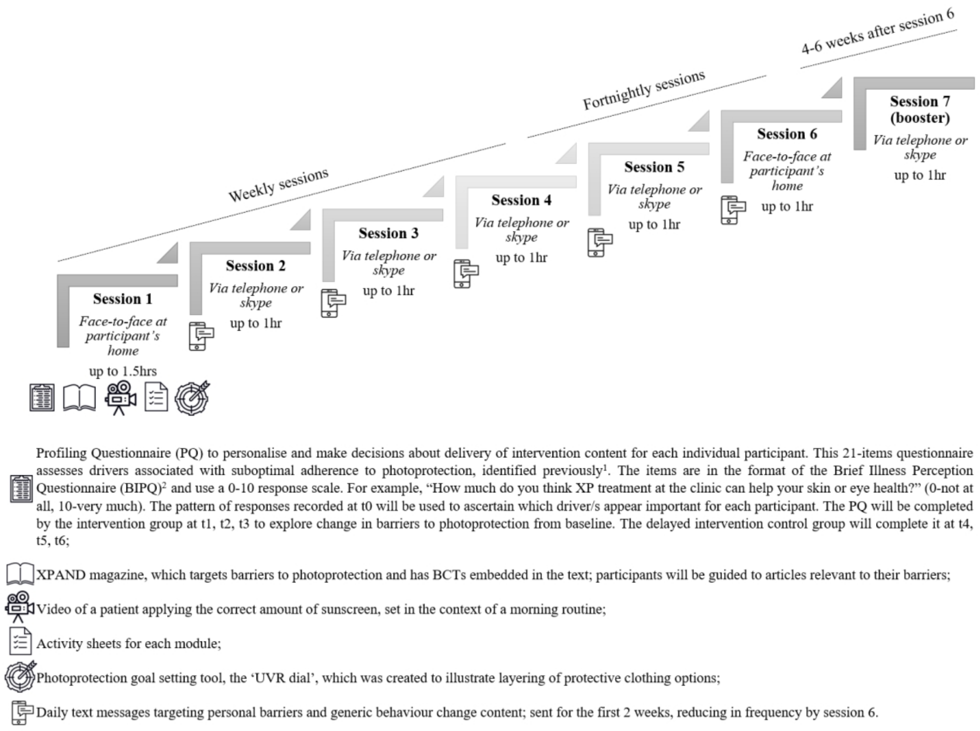

Figure 2. Structure of the XPAND intervention

Supplementary file 1: UVR Protection Diary

Your daily UVR diary

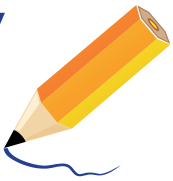

Study ID

For peer review only

« 21 DAYS

« 14 DAYS

« 7 DAYS

Your daily UVR diary

People with XP tell us that it can be hard to protect against UVR all the time and we are interested in what works for you.

This booklet has your daily diary sheets to record when you were outside and whether you did anything to protect against UVR each day.

- ★ You only need to record the time you spend outside if it was for **10 minutes or longer**
- ★ “OUTSIDE” is any amount of time longer than 10 minutes you **did not spend in a building**
  “OUTSIDE” includes time spent travelling in cars, buses or trains
- ★ Our grid is split into 15 minute blocks, start your line at the nearest block

We are interested in your views about your UVR protection each day and your general mood. There are four short questions to complete.
Don’t take too long thinking about your responses. Your immediate reaction to each question will probably be more accurate than a long thought out response. Please circle the number that best corresponds to your views on that day.

- ★ **If you can’t remember exactly or you are unsure give us your best guess!**
- ★ Try to fill out the diary at the end of each day. It will be easier to do when it is fresh in your mind. If you do forget, don’t worry! Fill it out as soon as you remember.

? **If you are unsure or have any questions contact Lesley on 07775111823 or email her at Lesley.Foster@gstt.nhs.uk**

Thank you very much for taking the time to complete the daily diary.

KING'S College LONDON

Guy’s and St Thomas’ NHS
NHS Foundation Trust

Daily diary example overleaf

IRAS 236877, Version 1, 21/11/2017 - UVR Protection Diary

For peer review only - http://bmjopen.bmj.com/site/about/guidelines.xhtml

For peer review only

Your daily **UVR diary**

Example:

1. **Date (day of week)** 14 May (Saturday)
2. **Did you go outside today?** (Please tick) **YES** ✓ **NO** ☐ If no, skip to question 4
3. **Let us know when you were outside by marking a line on the diary**

	6am	7am	8am	9am	10am	11am	12pm	1pm	2pm	3pm	4pm	5pm	6pm	7pm	8pm	9pm
I was outside																

4. **Let us know if you wore any of these things today by marking a line on the diary**

	6am	7am	8am	9am	10am	11am	12pm	1pm	2pm	3pm	4pm	5pm	6pm	7pm	8pm	9pm
Hat																
Glasses																
Scarf or facebuff																
Hoodie worn up																
Face visor																

5. **If you put sunscreen on your face or used lip sun block tell us each time you did this by ticking the diary**

	6am	7am	8am	9am	10am	11am	12pm	1pm	2pm	3pm	4pm	5pm	6pm	7pm	8pm	9pm
Sun screen	✓				✓				✓							
Lip sunblock	✓															

How would you describe your mood today?

0 1 2 3 4 5 6 7 8 9 (10)

Very negative … Very positive

How important was UVR protection of your face **today** compared to other things you wanted/needed to do?

0 1 2 3 (4) 5 6 7 8 9 10

Not at all … Very important

How much do you agree that UVR protection of your face **today** was something you did automatically without thinking?

0 1 2 3 4 5 6 (7) 8 9 10

Strongly disagree … Strongly agree

How confident are you that you can protect your face well **tomorrow**, even if other things get in the way?

0 1 2 3 4 (5) 6 7 8 9 10

Not at all … Very much so

« 7 DAYS « 14 DAYS « 21 DAYS

For peer review only - http://bmjopen.bmj.com/site/about/guidelines.xhtml

Supplementary File 2. Photoprotection self-efficacy questionnaire (PhotoSEQ)

Participant study code________________

XPAND

Enhancing Photoprotection Activities - New Directions

Xeroderma Pigmentosum National Service
2nd Floor, South Wing
St Thomas' Hospital
Westminster Bridge Road
London SE1 7EH

We are interested in your own personal views about XP and ultraviolet (UVR) protection. There are no right or wrong answers.

Please circle the number that best corresponds to your views:

1. How confident are you that you can lower your overall exposure to daylight by shifting when or how long you do outdoor daily activities?

   0 1 2 3 4 5 6 7 8 9 10
   not at all ... *very confident*

2. How confident are you that you can wear all the protective clothing you want, to protect yourself when outdoors?

   0 1 2 3 4 5 6 7 8 9 10
   not at all ... *very confident*

3. How confident are you that you can correctly apply sunscreen so that you have best protection from UVR?

   0 1 2 3 4 5 6 7 8 9 10
   not at all ... *very confident*

4. Thinking about using clothing (e.g., hat, glasses, scarf or buff, hoodie) to achieve a high level of photoprotection of your face from UVR over the next 4 weeks, how confident are you that you can photoprotect even if/when………

A. unexpected things get in the way

0 1 2 3 4 5 6 7 8 9 10
not at all ... *very confident*

B. you are somewhere new

0 1 2 3 4 5 6 7 8 9 10
not at all ... *very confident*

For peer review only

Supplementary File 2. Photoprotection self-efficacy questionnaire (PhotoSEQ)

C. you are in a rush

0 1 2 3 4 5 6 7 8 9 10

not at all *very confident*

D. you are with new people

0 1 2 3 4 5 6 7 8 9 10

not at all *very confident*

E. you don't want to look different

0 1 2 3 4 5 6 7 8 9 10

not at all *very confident*

F. you are feeling low

0 1 2 3 4 5 6 7 8 9 10

not at all *very confident*

G. you are feeling happy

0 1 2 3 4 5 6 7 8 9 10

not at all *very confident*

H. you are pressured not to protect by family or friends

0 1 2 3 4 5 6 7 8 9 10

not at all *very confident*

I. It might be physically uncomfortable (e.g., too hot)

0 1 2 3 4 5 6 7 8 9 10

not at all *very confident*

5. How confident you are that you will correctly apply sunscreen to your face over the next 4 weeks even if/when:

A. unexpected things get in the way

0 1 2 3 4 5 6 7 8 9 10

not at all *very confident*

For peer review only

Supplementary File 2. Photoprotection self-efficacy questionnaire (PhotoSEQ)

B. you are somewhere new

0 1 2 3 4 5 6 7 8 9 10

not at all *very confident*

C. you are in a rush

0 1 2 3 4 5 6 7 8 9 10

not at all *very confident*

D. you are with new people

0 1 2 3 4 5 6 7 8 9 10

not at all *very confident*

E. you don't want to look different

0 1 2 3 4 5 6 7 8 9 10

not at all *very confident*

F. you are feeling low

0 1 2 3 4 5 6 7 8 9 10

not at all *very confident*

G. you are feeling happy

0 1 2 3 4 5 6 7 8 9 10

not at all *very confident*

H. you are pressured not to protect by family or friends

0 1 2 3 4 5 6 7 8 9 10

not at all *very confident*

I. It might be physically uncomfortable (e.g., sticky)

0 1 2 3 4 5 6 7 8 9 10

not at all *very confident*

For peer review only

For peer review only - http://bmjopen.bmj.com/site/about/guidelines.xhtml

Supplementary file 3. Brief Photoprotection Adherence Questionnaire (BPAQ)

XPAND
Enhancing Photoprotection Activities - New Directions

Xeroderma Pigmentosum National Service
2nd Floor, South Wing
St Thomas' Hospital
Westminster Bridge Road
London SE1 7EH

We know people adjust ultraviolet radiation (UVR) protection to fit in with their everyday life. We are interested in how *<u>you</u>* protected against UVR in the last week.

Please circle the number that best corresponds to your views:

Thinking about your UVR protection over the last week

1. When you went outside, how often did you protect your face against UVR using protective clothing?

   0 *never* 1 2 3 4 5 6 7 8 9 10 *all the time*

2. How many days did you apply sunscreen on your face when getting ready in the morning?

   0 *none* 1 2 3 4 5 6 7 *every day of the week*

3. When you went outside for longer periods, how often did you reapply sunscreen on your face?

   0 *not at all* 1 2 3 4 5 6 7 8 9 10 *always*

4. On average, how many hours did you spend outside per day between 7am and 7pm?

   a) *Never*
   b) *30min or less*
   c) *31 min to 1 hour*
   d) *2 hours*
   e) *3 hours*
   f) *4 hours*
   g) *5 hours*
   h) *6 hours*
   i) *7 hours*
   j) *8 hour or more*

For peer review only

For peer review only - http://bmjopen.bmj.com/site/about/guidelines.xhtml

Supplementary file 3. Brief Photoprotection Adherence Questionnaire (BPAQ)

5. On average, how many hours did you spend outside per day between 11am and 3pm?

a) *Never*

b) *30min or less*

c) *31 min to 1 hour*

d) *2 hours*

e) *3 hours*

f) *4 hours*

THANK YOU VERY MUCH

For peer review only

For peer review only - http://bmjopen.bmj.com/site/about/guidelines.xhtml

Supplementary file 4. Intervention Feedback Questionnaire

IRAS 236877

XPAND

Enhancing Photoprotection Activities - New Directions

Xeroderma Pigmentosum National Service
2nd Floor, South Wing
St Thomas' Hospital
Westminster Bridge Road
London SE1 7EH

Feedback Questionnaire

We are interested in your opinion of the XPAND programme. Your views will contribute to development of the programme for people newly diagnosed with XP. We are interested in your views, there are no right or wrong answers.

Please tick the box to show how much you agree or disagree with the following statements about the XPAND programme.

1. Overall the programme was........

	1 Strongly disagree	2	3	4	5 Completely agree
Relevant to me					
Interesting					
Helpful					
Easy to understand					
Too long					
Too short					

Comments

For peer review only - http://bmjopen.bmj.com/site/about/guidelines.xhtml

Supplementary file 4. Intervention Feedback Questionnaire

Please give us reasons for your answers and say if there is anything you'd like us to change….

2. How helpful did you find the different parts of the programme?

	1<br>Not at all helpful	2	3	4	5<br>Very helpful
Face to face meeting with XPAND team member					
Telephone or skype calls with XPAND team member					
Printed materials					
Text messages					

Comments<br>Please explain why you found parts helpful or not helpful

For peer review only

Supplementary file 4. Intervention Feedback Questionnaire

3. Please tick the box to show how much you agree or disagree with the following statements about the XPAND programme?

The programme helped me to improve my UV protection

Strongly disagree	1	2	3	4	5	Completely agree

Comments
Tell us what you are doing differently? If nothing has changed please write “no change”.

4. The programme has made living with XP easier in other ways (e.g. feeling generally more confident or closer to family and friends)

Strongly disagree	1	2	3	4	5	Completely agree

Comments
Could you tell us how? If nothing has changed please write “no change”.

For peer review only - http://bmjopen.bmj.com/site/about/guidelines.xhtml

Supplementary file 4. Intervention Feedback Questionnaire

5. Overall the programme has changed……..

	1 Strongly disagree	2	3	4	5 Completely agree
My motivation to protect					
My confidence in managing protection					
My knowledge					
My routines in managing protection					
My ways of coping when life gets in the way of protection					
How my friends and family support me					

Comments<br>Can you tell us how these things have changed? If nothing has changed please write “no change”.

For peer review only - http://bmjopen.bmj.com/site/about/guidelines.xhtml

Supplementary file 4. Intervention Feedback Questionnaire

6. Would you recommend taking part in XPAND to other people with XP?

Yes ☐ No ☐

Please add any other comments here

Comments

For peer review only

THANK YOU VERY MUCH

For peer review only - http://bmjopen.bmj.com/site/about/guidelines.xhtml

Supplementary file 5. Service Use Questionnaire

XPAND
Enhancing Photoprotection Activities - New Directions

Service use and Financial costs of XP to you and

your family in the last 6 months

Xeroderma Pigmentosum National Service
2nd Floor, South Wing
St Thomas' Hospital
Westminster Bridge Road
London SE1 7EH

For peer review only

These questions are about the services that you have accessed and the financial cost that the XP causes for you and your family. It is fine if you make your best guess for questions where you are not sure of the answer.

1. **In the last 6 months, have you attended the XP clinic at Guy's and St Thomas' hospital? Please complete the following sentence:**

   a) **I have attended the clinic ____ times in the last 6 months**

If you did not attend the XP clinic in the last 6 months, go to question 2.

If you have attended the clinic in the last 6 months:

   b) **How long did the journey from home to the clinic take? _____ hours _____ minutes**

   c) **Did you have to spend the night away from home in order to visit the clinic?**
   Yes, I stayed in hospital
   Yes, but not in hospital
   No

   d) **Which specialists do you recall seeing on your visit(s) to the XP clinic? Please tick yes or no for each specialist**

**Specialist**	**Yes, I saw this person**	**No, I did not see this person**
Dermatologist		
Ophthalmologist		
Neurologist		
Geneticist		
Psychologist		

Supplementary file 5. Service Use Questionnaire

Specialist Nurse		
Plastic Surgeon		

2. Please complete the following sentence:
In the last 6 months, I have been to Accident and Emergency because of my XP ____ times.

3. Please give details of any hospital outpatient appointments you have had in the last 6 months <u>because of your XP. Do not include trips to the XP clinic at Guy's and St Thomas' here.</u>

**Speciality**	**Number of XP related visits in the last 6 months**	**If you paid for this service, roughly how much did it cost per visit?**	**Roughly how long did it take to travel to the appointment?**
Dermatology			___ hours __ minutes
Ophthalmology			___ hours __ minutes
Neurology			___ hours __ minutes
Paediatrics			___ hours __ minutes
Plastic Surgery			___ hours __ minutes
Psychologist			___ hours __ minutes

4. What surgery you have had in the <u>LAST 6 MONTHS</u>? (day or clinic surgery - where you did <u>not</u> stay in hospital overnight)

**Type of surgery**	**Approximate number of surgeries**	**If you paid for this service, approximately how much did it cost per episode of surgery?**	**Roughly how long did it take to travel for the surgery?**
Skin biopsy			___ hours __ minutes
Surgery to the skin			___ hours __ minutes
Surgery to the eyes			___ hours __ minutes
Other XP related surgery<br>What sort of surgery was it?<br>____________________			___ hours __ minutes

For peer review only

Supplementary file 5. Service Use Questionnaire

5. Please complete the following sentence.
In the last 6 months, I have been admitted to hospital and stayed overnight ___ times because of my XP. In this time, I spent a total of ___ nights in hospital.

For peer review only

Supplementary file 5. Service Use Questionnaire

6. In the last 6 months, which of these services have you visited or been visited at home by, because of your XP?

	**Number of appointments at surgery or clinic**	**Number of home visits**	**If you paid, approximately how much did it cost per visit?**	**How long did it take to travel for the appointment each time if it was not at home?**
General practitioner				___ hours __ minutes
Nurse				___ hours __ minutes
Physiotherapist				___ hours __ minutes
Psychologist , Counsellor or Psychotherapist				___ hours __ minutes
'Alternative' medicine or 'complementary' therapist				___ hours __ minutes
Occupational therapist				___ hours __ minutes
	**Number of appointments at surgery or clinic**	**Number of home visits**	**If you paid, approximately how much did it cost per visit?**	**How long did it take to travel for the appointment each time if it was not at home?**

For peer review only

Supplementary file 5. Service Use Questionnaire

Social worker				___ hours __ minutes
Home help or home care worker				___ hours __ minutes
Self-help or support group				___ hours __ minutes

7. Please list any tests you remember that you have had done in the LAST 6 MONTHS because of your XP

**Service**	**How many times?**	**If you paid, approximately how much did it cost?**
MRI (Magnetic Resonance Image) brain scan		
Audiometry		
Nerve testing (nerve conduction or EMG test)		
Blood test		
Other investigations / tests<br><br>What was it?<br><br>…………………………………………………………		

For peer review only

Supplementary file 5. Service Use Questionnaire

8. In the last 6 months, have you received help from friends or relatives with any of the following tasks, as a result of your XP?

**Type of help**	**Please Circle**	**Approximate number of hours they help you in a week**
Child Care<br>(circle 'No' if you have no children)	**No Yes**	
Personal care<br>*(for example washing, dressing)*	**No Yes**	
Help in the house<br>(for example cooking or cleaning)	**No Yes**	
Help outside the house<br>(for example shopping or transport)	**No Yes**	
Going with you to medical appointment	**No Yes**	

9. If you answered yes to any help in the previous question, have your helper(s) taken any time out of work or education to help you as a result of your XP?
If there are part-days to consider, assume that 1 day = 8 hours.
______________ days taken off from work in the last 6 months
______________ days taken off from education in the last 6 months

10. What is your employment status today? Please tick the one that best describes your situation

Paid employment - full-time	
Paid employment – part-time (working 30 or fewer hours per week on average)	
Unemployed – registered unemployed, available and looking for work	
Unemployed – unable to work due to illness	
Unpaid voluntary work	

For peer review only

Supplementary file 5. Service Use Questionnaire

Student	
Retired	
Housewife/husband	

11. If you have been in education in the last 6 months, approximately how many days have you been off due to health problems? _____________ days

12. If you have been in work in the last 6 months, approximately how many days have you been off due to health problems? _____________ days

13. Please circle the appropriate answer:
The impact of XP on the household finances (for example on what you can spend on food, clothes, and holidays) has been
Very small Small Moderate Large Very large

14. Approximately how much would you estimate that you / your household has paid in the last 6 months for these items? If you paid for items that were a one-off cost longer than 6 months ago, please record these in the "one-off cost" column *(take a best and approximate guess, and do not spend too long on this – we realise it is not possible to be completely accurate for this!)* :

	**Approximate cost in last 6 months to you**	**A one-off cost**
Sunscreen creams		
UV protective window films		
Other house or car adaptations to protect against UV		
UV visor		
UV protective clothes		
Glasses		
Vitamin D tablets		
UV meter		

For peer review only

Supplementary file 5. Service Use Questionnaire

15. Please list any medication that you have taken in the last 6 months, because of your XP

Medication name and strength	Length of time that you took / have taken the medication for out of the last 6 months
	___ months ___ weeks ___ days
	___ months ___ weeks ___ days
	___ months ___ weeks ___ days
	___ months ___ weeks ___ days

**16. What is the date today? (day/month/year)**	

THANK YOU VERY MUCH FOR COMPLETING OUR QUESTIONNAIRE.

For peer review only

SPIRIT 2013 Checklist: Recommended items to address in a clinical trial protocol and related documents*

Section/item	Item No	Description	Page No
**Administrative information**			
Title	1	Descriptive title identifying the study design, population, interventions, and, if applicable, trial acronym	1
Trial registration	2a	Trial identifier and registry name. If not yet registered, name of intended registry	4
	2b	All items from the World Health Organization Trial Registration Data Set	n/a
Protocol version	3	Date and version identifier	Header
Funding	4	Sources and types of financial, material, and other support	22
Roles and responsibilities	5a	Names, affiliations, and roles of protocol contributors	1, 22
	5b	Name and contact information for the trial sponsor	22
	5c	Role of study sponsor and funders, if any, in study design; collection, management, analysis, and interpretation of data; writing of the report; and the decision to submit the report for publication, including whether they will have ultimate authority over any of these activities	22
	5d	Composition, roles, and responsibilities of the coordinating centre, steering committee, endpoint adjudication committee, data management team, and other individuals or groups overseeing the trial, if applicable (see Item 21a for data monitoring committee)	20
**Introduction**			
Background and rationale	6a	Description of research question and justification for undertaking the trial, including summary of relevant studies (published and unpublished) examining benefits and harms for each intervention	5-7
	6b	Explanation for choice of comparators	5-7

For peer review only - http://bmjopen.bmj.com/site/about/guidelines.xhtml

Objectives	7	Specific objectives or hypotheses	7
Trial design	8	Description of trial design including type of trial (eg, parallel group, crossover, factorial, single group), allocation ratio, and framework (eg, superiority, equivalence, noninferiority, exploratory)	9
**Methods: Participants, interventions, and outcomes**			
Study setting	9	Description of study settings (eg, community clinic, academic hospital) and list of countries where data will be collected. Reference to where list of study sites can be obtained	7
Eligibility criteria	10	Inclusion and exclusion criteria for participants. If applicable, eligibility criteria for study centres and individuals who will perform the interventions (eg, surgeons, psychotherapists)	8
Interventions	11a	Interventions for each group with sufficient detail to allow replication, including how and when they will be administered	15-16
	11b	Criteria for discontinuing or modifying allocated interventions for a given trial participant (eg, drug dose change in response to harms, participant request, or improving/worsening disease)	20
	11c	Strategies to improve adherence to intervention protocols, and any procedures for monitoring adherence (eg, drug tablet return, laboratory tests)	18
	11d	Relevant concomitant care and interventions that are permitted or prohibited during the trial	9
Outcomes	12	Primary, secondary, and other outcomes, including the specific measurement variable (eg, systolic blood pressure), analysis metric (eg, change from baseline, final value, time to event), method of aggregation (eg, median, proportion), and time point for each outcome. Explanation of the clinical relevance of chosen efficacy and harm outcomes is strongly recommended	10-15
Participant timeline	13	Time schedule of enrolment, interventions (including any run-ins and washouts), assessments, and visits for participants. A schematic diagram is highly recommended (see Figure)	Figures 1, 2. Table 1

For peer review only - http://bmjopen.bmj.com/site/about/guidelines.xhtml

Sample size	14	Estimated number of participants needed to achieve study objectives and how it was determined, including clinical and statistical assumptions supporting any sample size calculations	16-18
Recruitment	15	Strategies for achieving adequate participant enrolment to reach target sample size	7-8
**Methods: Assignment of interventions (for controlled trials)**			
Allocation:			
Sequence generation	16a	Method of generating the allocation sequence (eg, computer-generated random numbers), and list of any factors for stratification. To reduce predictability of a random sequence, details of any planned restriction (eg, blocking) should be provided in a separate document that is unavailable to those who enrol participants or assign interventions	10
Allocation concealmen t mechanism	16b	Mechanism of implementing the allocation sequence (eg, central telephone; sequentially numbered, opaque, sealed envelopes), describing any steps to conceal the sequence until interventions are assigned	10
Implementat ion	16c	Who will generate the allocation sequence, who will enrol participants, and who will assign participants to interventions	10
Blinding (masking)	17a	Who will be blinded after assignment to interventions (eg, trial participants, care providers, outcome assessors, data analysts), and how	10, 16
	17b	If blinded, circumstances under which unblinding is permissible, and procedure for revealing a participant's allocated intervention during the trial	Can be obtained from authors
**Methods: Data collection, management, and analysis**			
Data collection methods	18a	Plans for assessment and collection of outcome, baseline, and other trial data, including any related processes to promote data quality (eg, duplicate measurements, training of assessors) and a description of study instruments (eg, questionnaires, laboratory tests) along with their reliability and validity, if known. Reference to where data collection forms can be found, if not in the protocol	9-15

	18b	Plans to promote participant retention and complete follow-up, including list of any outcome data to be collected for participants who discontinue or deviate from intervention protocols	n/a
Data management	19	Plans for data entry, coding, security, and storage, including any related processes to promote data quality (eg, double data entry; range checks for data values). Reference to where details of data management procedures can be found, if not in the protocol	20-21
Statistical methods	20a	Statistical methods for analysing primary and secondary outcomes. Reference to where other details of the statistical analysis plan can be found, if not in the protocol	16-17
	20b	Methods for any additional analyses (eg, subgroup and adjusted analyses)	16-17
	20c	Definition of analysis population relating to protocol non-adherence (eg, as randomised analysis), and any statistical methods to handle missing data (eg, multiple imputation)	Can be obtained from authors
**Methods: Monitoring**			
Data monitoring	21a	Composition of data monitoring committee (DMC); summary of its role and reporting structure; statement of whether it is independent from the sponsor and competing interests; and reference to where further details about its charter can be found, if not in the protocol. Alternatively, an explanation of why a DMC is not needed	20-21
	21b	Description of any interim analyses and stopping guidelines, including who will have access to these interim results and make the final decision to terminate the trial	n/a
Harms	22	Plans for collecting, assessing, reporting, and managing solicited and spontaneously reported adverse events and other unintended effects of trial interventions or trial conduct	20-21
Auditing	23	Frequency and procedures for auditing trial conduct, if any, and whether the process will be independent from investigators and the sponsor	Can be obtained from authors
**Ethics and dissemination**			

For peer review only - http://bmjopen.bmj.com/site/about/guidelines.xhtml

Research ethics approval	24	Plans for seeking research ethics committee/institutional review board (REC/IRB) approval	10
Protocol amendments	25	Plans for communicating important protocol modifications (eg, changes to eligibility criteria, outcomes, analyses) to relevant parties (eg, investigators, REC/IRBs, trial participants, trial registries, journals, regulators)	Can be obtained from authors
Consent or assent	26a	Who will obtain informed consent or assent from potential trial participants or authorised surrogates, and how (see Item 32)	7-8
	26b	Additional consent provisions for collection and use of participant data and biological specimens in ancillary studies, if applicable	n/a
Confidentiality	27	How personal information about potential and enrolled participants will be collected, shared, and maintained in order to protect confidentiality before, during, and after the trial	20-21
Declaration of interests	28	Financial and other competing interests for principal investigators for the overall trial and each study site	22
Access to data	29	Statement of who will have access to the final trial dataset, and disclosure of contractual agreements that limit such access for investigators	Can be obtained from authors
Ancillary and post-trial care	30	Provisions, if any, for ancillary and post-trial care, and for compensation to those who suffer harm from trial participation	n/a
Dissemination policy	31a	Plans for investigators and sponsor to communicate trial results to participants, healthcare professionals, the public, and other relevant groups (eg, via publication, reporting in results databases, or other data sharing arrangements), including any publication restrictions	22
	31b	Authorship eligibility guidelines and any intended use of professional writers	n/a
	31c	Plans, if any, for granting public access to the full protocol, participant-level dataset, and statistical code	n/a
**Appendices**			

For peer review only - http://bmjopen.bmj.com/site/about/guidelines.xhtml

Informed consent materials	32	Model consent form and other related documentation given to participants and authorised surrogates	Can be obtained from authors
Biological specimens	33	Plans for collection, laboratory evaluation, and storage of biological specimens for genetic or molecular analysis in the current trial and for future use in ancillary studies, if applicable	n/a

*It is strongly recommended that this checklist be read in conjunction with the SPIRIT 2013 Explanation & Elaboration for important clarification on the items. Amendments to the protocol should be tracked and dated. The SPIRIT checklist is copyrighted by the SPIRIT Group under the Creative Commons "Attribution-NonCommercial-NoDerivs 3.0 Unported" license.

For peer review only
